# Supplementary material for: The NoHoW protocol: a multicentre 2×2 factorial randomised controlled trial investigating an evidence-based digital toolkit for weight loss maintenance in European adults
Source: BMJ Open. 2019 Sep 30;9(9):e029425. doi: 10.1136/bmjopen-2019-029425 (PMC6773359; doi:10.1136/bmjopen-2019-029425)
Supplement: Supplementary data [file bmjopen-2019-029425supp001.pdf]

Supplementary file 1. Example consent form.

CONSENT FORM

NoHoW: Evidence-based ICT tools for weight loss maintenance

Please read the following statements and initial each box, as in order to continue in the study you must agree to the following statements

☐ I confirm that I have read and understood the participant information sheet (version 1, 27/02/2017), for the study named above and I have had the opportunity to ask questions and have had these answered satisfactorily.

☐ I consent for the research team to use the anonymised information obtained for research purposes (e.g., disseminating the results at conferences, in publications or for training purposes) and understand that the anonymised data will be kept for 20 years.

☐ I understand that information specific to this project may be looked at by responsible individuals from the research teams and will not be shared to anyone outside of the research teams.

☐ I understand that my participation is voluntary and that I am free to withdraw at any time, without giving any reason, without my medical care, or legal rights being affected.

☐ I agree to be contacted to be asked to fill in questionnaires and for measures to be taken initially at baseline, 6 months, 12 months and 18 months after the study has begun.

The following sections are optional- if you agree with a statement, please initial the box:

☐ I agree to take part in the study.

☐ I agree to provide a sample of a small section of hair, for the assessment of cortisol content, as a biomarker of longer term stress, initially at baseline and 12 months after.

Donation of blood for research is voluntary and you should not be placed under any pressures to do so. You do not have to agree to give a blood sample nor need to explain why you should choose not to donate. Any personal information provided by you in connection with the donation will be held in confidence. For reasons of safety, you should not donate if:

- You know, or think that you might be infected with Hepatitis B or Hepatitis C.
- You know, or think that you might be infected with HIV.
- You have a sexual partner who is infected with Hepatitis or HIV.
- You are unwell at the moment.
- You are anaemic or receiving treatment for anaemia or iron deficiency.

☐ I agree to provide a small blood sample by finger prick initially at baseline, and 12 months after.

☐ I wish to receive written feedback of my blood results which will include results of my full lipid profile (e.g., cholesterol), and HbA1c levels.

☐ Occasionally, results may fall out of the usual range which may be due to conditions such as high blood pressure, pre-diabetes, diabetes and high cholesterol. In these cases, we may wish to share the results of your measurements with yourself and your GP. I agree for the researchers to contact me and my GP

Name of participant: ………………………………………

Signature of participant: …………………………………… Date: …………….

I confirm that the volunteer named above has been instructed of the nature and purpose of the study to be undertaken.

Signature of researcher:……………………………………………….. Date: ………………

The NoHoW Project has been approved by the School of Psychology Research Ethics Committee at the University of Leeds (17-0082; 27-Feb-2017). If you have any questions, please contact the principal Investigator, Professor James Stubbs, at r.j.stubbs@leeds.ac.uk.
