# Supplementary material for: The NoHoW protocol: a multicentre 2×2 factorial randomised controlled trial investigating an evidence-based digital toolkit for weight loss maintenance in European adults
Source: BMJ Open. 2019 Sep 30;9(9):e029425. doi: 10.1136/bmjopen-2019-029425 (PMC6773359; doi:10.1136/bmjopen-2019-029425)
Supplement: Supplementary data [file bmjopen-2019-029425supp002.pdf]

Supplementary File 2. Study assessment schedule (SPIRIT 2013 guidelines).

		Study Duration				
		Recruitment	Baseline	6 months	12 months	18 months
**MEASURES**	**DESCRIPTION**	t_0	t_1	t_2	t_3	t_4
**DEMOGRAPHICS.**	Age, gender, occupation, education level, income, height, marital status and employment, smoking and alcohol history.	X	X			
**WEIGHT HISTORY.**	Self-report of current weight, weight 12 months ago, highest weight in last 12 months, highest weight, pregnancy, weight loss attempts, weight maintenance, relapse, weight loss methods, and parent's weight history.		X			
**MEDICAL HISTORY**	Name and dosage of medications, medical conditions, stability of conditions.	X	X	X	X	X
**DIGITAL HEALTH LITERACY.**	The eHEALS scale[84] assesses digital health literacy (i.e. knowledge of different health information on the internet and perceived confidence in one's ability to find or use such information to make health decisions). The scale consists of 8 items measured on a 5-point Likert scale ('1', Strongly disagree, to '5' Strongly agree).		X			
**ANTHROPOMETRICS**						
Weight.	Body weight (Seca 704s, SECA, Germany) in light clothing at 0,6, 12 and 18 months and at home throughout the duration of the trial via the Fitbit Aria smart scales ≥ twice weekly.		◆	—	—	◆

Height	Height (±0.1cm) is measured with participants barefoot, using a Seca 704s instrument (SECA, Germany).		X			
Body mass index	Body mass index (BMI) is calculated from weight (kg) and height (m) using the following equation: kg/m^2.	X	X	X	X	X
Waist hip ratio.	The waist measurement is taken against bare skin or light clothing at the top of the iliac crest and the hip measurement is taken at the maximum circumference over the buttocks using a tape measure. Average waist hip ratio measured by taking 2 fasted hip and waist circumference measures, or 3 if there is >1cm difference. The waist-hip ratio is calculated by dividing hip and waist circumference.		X	X	X	X
**BODY COMPOSITION**	Fasting bioimpedance (ImpediMed™ SFB7, Queensland, Australia) estimates total body water, extra- and intracellular fluid (l) and hence fat free mass (kg) and by subtraction fat mass (kg). It includes applying an electrical current at 256 frequencies ranging from 4 kHz to 1000 kHz, via a tetrapolar electrode configuration, following manufacturer's instructions. Assuming a constant hydration factor of 0.73 for fat free mass and that fat mass is hydrophobic, body water and weight can be used to estimate fat and fat free mass using a 2 compartment model.		X	X	X	X
**BIO-MARKERS**						
HbA1c.	Fasting capillary blood is analysed for HbA1c (mmol/mol, eAG and %) using the Afinion™ AS100 Analyzer (Alere, Stockport, UK). Sample cartridges are disposed of immediately after results are obtained from the machine.		X		X	
Full lipid profile.	Fasting capillary blood is analysed for full lipid profiles including total cholesterol (mmol/L), low density lipoprotein (LDL), high density lipoprotein (HDL), triglycerides, non-HDL and cholesterol/HDL (mmol/L) using the AfinionTM AS100 Analyzer (Alere). Sample cartridges are disposed of immediately after results are obtained from the machine.					
Hair samples.	Cortisol levels are measured by analysing 2cm of hair cut from close to the scalp. The sample is stored in		X		X	

	foil inside a sealed plastic bag. User ID and hair information (e.g., the use of hair dye, hair product use and hair wash frequency per week) is stored with the sample. Between 10 and 20 mg of hair from the 2 cm of the tuft closest to the scalp is accurately weighed, minced finely 1ml of methanol is added and the suspension incubated overnight at 50°C with a gentle shaking. The following day, the methanol is transferred into a clean tube and evaporated to dryness under nitrogen. The residue is reconstituted in 250-µl PBS buffer (pH 8.0). The cortisol concentration (pg/mg) in the resulting buffer solution is determined in duplicate using Enzyme-linked immunosorbent assay (ELISA).[48]					
Blood pressure.	An average of three fasting pulse, diastolic and systolic blood pressure measures are calculated and measured using the Microlife BP A2 (Gentle Technology, Microlife, Clearwater, FL, USA, Inc).		X	X	X	X
Heart rate.	Daily average resting heart rate is measured from minute-by-minute data collected throughout the duration of the trial via the Fitbit Charge 2.		♦	—	—	♦
**PHYSICAL ACTIVITY**						
Daily steps	Minute-by-minute steps are monitored by Fitbit Charge 2 for trial duration. The Charge 2 TM uses a wrist-worn tri-axial accelerometer, altimeter and vibration motor, which collects longitudinal movement data and estimates activity intensity, duration, number of steps, and distance travelled using proprietary algorithms (https://www.fitbit.com/charge2).		♦	—	—	♦
Intensity of physical activity (low, moderate and vigorous).	Time (minutes/day) spent in each intensity of activity is measured via self-report using the International Physical Activity Questionnaire (IPAQ) long-version[56] and is objectively measured using minute-by-minute activity monitored by Fitbit Charge 2 for trial duration.		X	X	X	X
Sitting behaviour.	Time (minutes/day) spent sitting is measured using the IPAQ.[56]		X	X	X	X
Activity choice.	The Activity Choice Index (ACI)[57] measures the ability to choose physical activity over less active or sedentary behaviours, such as using stairs or escalators. The scale consists of a 5-point Likert scale ('1',					

	never, to '5' always).					
**SLEEP QUALITY AND QUANTITY**	Sleep quantity (hours/day) and quality are monitored by the Fitbit Charge 2 for the trial duration.		♦	—	—	♦
**DIETARY INTAKE AND EATING BEHAVIOUR**						
Dietary intake.	Four consecutive days of 24-hour dietary recalls, including one weekend day, using the web-based tool INTAKE24[58], which measures food intake and estimates daily energy (kcal) and nutrient intake from standardised food tables. Country specific food tables were inserted into the platform.		X	X	X	X
Cognitive restraint, disinhibition and hunger.	The Three Factor Eating Questionnaire (TFEQ)[59] is a 51-item, 3 factor measure that assesses cognitive restraint, disinhibition and hunger. The scale uses a true or false scale (items 1-36), a 4-point scale for items 37-49 and 51 ('1', rarely, to '4', always) and a 6-point scale for item 50 ('0', I eat whenever I want, to '5', constantly limiting food intake).		X	X	X	X
Controllability and automaticity.	Controllability and Automaticity of Eating Behaviour Questionnaire (CAEBQ) (unpublished scale) is a 24-item, 2 factor measure assessing eating control, grazing, binging and eating in the absence of hunger. Each subscale consists of 2-items assessed on a 5-point scale ('1', never, to '5', very often) and 4 items consisting of a 5-point Likert scale ('1' strongly disagree, to '5', strongly agree).		X	X	X	X
Eating in the absence of hunger.	Eating in The Absence of Hunger Scale (EAHS)[60] is a 28-item, single factor scale assessing the conditions when one is likely to eat in the absence of hunger. The 28 items assesses eating in response to emotional, physical, and environmental cues, using a 5-point scale ('1', never, to '5', always).		X	X	X	X
Reliance on hunger and satiety.	The reliance on hunger and satiety cues is a 6-item subscale of the Intuitive Eating Scale-2 (IES-2)[61] that measures the ability to guide eating behaviour based on one's internal hunger and satiety signals. The subscale uses a 5-point Likert scale ('1', strongly disagree, to '5', strongly agree).		X	X	X	X

Binge Eating.	The Binge Eating Scale (BES)[62] is a 16-item one-dimensional measure assessing the severity of binge eating symptoms. Each item consists of three to four statements from which respondents select one. Each option reflects a rating of severity ranging from 0 (no difficulties with binge eating) to 3 (severe binge eating symptoms).		X	X	X	X
Intuitive eating – 1.	Intuitive Eating Scale – 1 (IES-1)[53] is a 21-item trait measure to assess the ability to be aware and follow physical hunger and satiety cues to determine when, what and how much to eat. It includes three subscales: eating for physical rather than emotional reasons, unconditional permission to eat and reliance on hunger and satiety cues. IES-1 uses a 5-point Likert scale ('1' strongly disagree, to '5' strongly agree). The full scale is used at baseline only.		X			
Power of food.	Power of Food Scale[52] is a 10-item measure adapted from the original 15 item version which assesses the sensitivity to palatable foods within food-abundant environments. It includes three subscales: food availability, food present and food tasted. PFS uses a 5-point Likert scale ('1' do not agree at all, to '5', strongly agree).		X			
**WELL-BEING AND QUALITY OF LIFE**						
Quality of Life.	The EQ5D-5L[63] measures generic health related quality of life (HRQoL). It includes five dimensions (mobility, self-care, usual activities, pain/discomfort, and anxiety/depression) and has five levels of severity within each dimension: no problems (Level 1); slight; moderate; severe; and extreme problems (Level 5).		X	X	X	X
Wellbeing.	The 14-item single factor Warwick and Edinburgh Well-being Scale[64] measures mental wellbeing using 14 positively worded statements and uses a 5-point scale ('1', none of the time, to '5', all of the time).		X	X	X	X
**SELF-REGULATION, SELF-EFFICACY AND**						

**MOTIVATION**						
Action planning and coping planning	The Action Planning and Coping Planning Scales (APCP)[65] measures coping planning as a barrier-focused self-regulation strategy. The Action planning scale consists of 4 items and coping planning consists of 15 items both using a 5-point Likert scale ('1', Strongly disagree, to '5', Strongly agree').		X	X	X	X
Action control	The Action Control Scale[66] consists of 8 items and uses a 5 point Likert scale ('1', Strongly disagree, to '5', Strongly agree') that measures three distinct self-regulatory actions: self-monitoring, awareness of standards, and effort.		X	X	X	X
Basic psychological needs and frustration	The Basic Psychological Needs and Frustration Scale[67] has 16 items that uses a 7-point scale ('1', Strongly disagree to, '7', Strongly agree') to assess basic need satisfaction with 4 subscales: autonomy, competence, social support and relatedness. Items 2, 4, 6, 8, 12, and 16 were adapted to focus on weight management (e.g. 'I feel capable of managing my weight').		X	X	X	X
Goal content	The Goal Content for Weight Loss Maintenance Scale (GCWLMS)[68,69] was modified to focus on weight management rather than exercise behaviours (e.g. 'I manage my weight to…'). It consists of 16 items that uses a 7-point scale ('1', Strongly disagree, to '7', Strongly agree') to assess intrinsic and extrinsic goals.		X	X	X	X
Behavioural regulation in exercise	The Behavioural Regulation in Exercise Scale (BREQ-3)[71] includes 4 subscales that measure external, introjected, identified and intrinsic forms of regulation of exercise behaviour. It consists of 24 items and uses a 5-point scale ('0', Not true for me, to '4', Very true for me).		X	X	X	X
Regulation for eating behaviour	The Regulation for Eating Behaviour Scale (REBS)[71] is the shortened scale and consists of 18 items that assess autonomous and controlled motivation, and associated types of regulation (e.g. introjected regulation) for eating behaviour, using a 7-point scale ('1', Does not correspond at all, to '7', Corresponds exactly). One item from each sub-scale (items 3, 6, 10, 11, 18, 20) displayed unsatisfactory psychometric properties and were removed to create a shortened scale.		X	X	X	X
Self-efficacy	The Self-Efficacy for Weight Management Scale (SEWM)[72] has 12 items and uses a 100 point sliding		X	X	X	X

	scale ('0', not confident at all, to '100', extremely confident) to asses self-efficacy beliefs for exercise and eating.					
Autonomy	The Index of Autonomous Functioning (IAF)[50] is a 15-item scale and uses a 5-point scale ('1', Not at all true, to '5', Completely true) to assess trait autonomy across three theoretically derived subscales: authorship/self-congruence, interest-taking, and low susceptibility to control.		X			
Relapse Prevention	The Relapse Prevention Scale (RPS)[54] is a newly developed scale for trait-level weight management and consists of 14 items that measures three sub-domains: approach-based plans/goals, avoidance-based plans/goals, and cognitive factors. The scales uses 5-point scale ('1', Very unlikely, to '5', Very likely).		X			
Life goals and aspirations	The Aspirations Index (AI)[51] is an 11-item scale that measures the degree to which people value seven broad goal contents: wealth, fame, image, personal growth, relationships, community contribution, and health. The scale uses a 9-point scale ('1', Not at all important, to '9', Extremely important).		X			
Regulation for weight management.	The Regulation for Weight Management Scale (RWMS)[71] consists of 10 items and measures the reasons that underpin individual's decisions to manage their weight (e.g. "Managing my weight is the part of the way I have chosen to live my life"). The scales uses a 7-point Likert scale ('1', Does not correspond at all, to '7', Corresponds exactly) and was adapted from the Regulation of Eating Behaviour scale (REBS) to focus specifically on weight management.		X	X	X	X
**EMOTION REGULATION**						
Self-criticism and reassurance	The Weight Focused Self-Criticism/Self-Reassuring Scale (WFSCRS)[73] is a 22-item scale that assesses one's thoughts and feelings about themselves during a perceived failure. Three subscales consist of self-criticising, (inadequate self and hated self) and tendencies to reassure the self (reassured self). The scale uses a 4-point scale ('0', Not at all like me, '4', Extremely like me) and was adapted to focus on weight, body shape, and eating.		X	X	X	X

External shame	The Weight-Focused External Shame Scale (WFESS)[74] is a one-dimensional measure with 18 items that measure external shame (e.g., thinking that others negatively evaluate the self) and uses a 4-point scale ('0', Never, to '4', Almost always).		X	X	X	X
Compassion	The Compassionate Attributes and Actions Scales (CAAS)[76] consist of three scales that measure self-compassion, compassion for others, and compassion from others. In this study, we omit the compassion for others scale as it is unrelated to the intervention logic models. Each scale consists of 6 items and uses a 10-point scale ('1', Never; to, '10', Always).		X	X	X	X
Body Image Acceptance	The Body Image Acceptance and Action Questionnaire (BIAAQ)[77] is a 12-item one-dimensional scale that measures body image-related psychological flexibility and the extent to which an individual accepts their beliefs and feelings about their body without attempting to change them while pursuing chosen values. The scale uses a 7-point scale ('1' Never true, to '7' Always true).		X	X	X	X
Engaged response (valued living and life-fulfilment)	The Engaged Living Scale (ELS)[78] consists of 16 items and measures two factors associated with psychological flexibility in line with Acceptance and Commitment Therapy: values living and life fulfilment. The scale uses a 5-point Likert Scale ('1', Completely disagree, to '5', Completely agree).		X	X	X	X
De-centering	The Experiences Questionnaire (EQ_DC)[79] has 11 items that assess three facets of the de-centering construct, including: (1) the ability to view the self as not synonymous with thoughts and feelings; (2) the ability to not habitually react to negative experiences ; and, (3) the capacity for self-compassion. The scale provides a total score of decentering and uses a 5-item scale ('1', Never, to '5', Always).		X	X	X	X
Mindful attention and awareness.	The Mindful Attention Awareness Scale (MAAS)[79] is a 15-item one-dimensional scale designed to assess dispositional mindfulness, that is, a receptive state of mind informed by a sensitive awareness of what is occurring in the present moment. The scales uses a 6-point scale ('1' Almost always, to '6' Almost never).		X	X	X	X
Emotion regulation.	The Difficulties in Emotion Regulation Scale (DERS)[80] is a one-dimensional scale that assesses the difficulty to regulate emotional responses by using adaptive emotion regulation strategies in the presence of negative emotions so that it does not lead to loss of control over behaviour or goal attainment. The scale consists of 16 items and uses a 5-point scale ('1', Almost always, to '5', Almost never) and is adapted for		X	X	X	X

	weight loss maintenance.					
**STRESS MANAGEMENT**						
Perceived stress.	The Perceived Stress Scale (PSS)[81] measures the degree to which people perceive their lives as stressful (unpredictable, uncontrollable and overloaded) in the last month. The scale is one-dimensional and consists of 4 items and uses a 5-point scale (‘0’, Never, to ‘4’, Very often).		X	X	X	X
Depression and anxiety.	The Depression and Anxiety Scale (DASS)[82] short version consists of 21 items and measures three related negative emotional states individuals experienced over the last week, through 3 subscales with 7 items each: depression, anxiety and stress. The scales uses a 4 point scale (‘0’, Did not apply to me at all, to ‘3’, Applied to me very much).		X	X	X	X
**PROCESS EVALUATION AND USER EXPERIENCE**						
Initial toolkit impressions.	Initial impressions of the NoHoW Toolkit are captured at 1 month post-baseline and is adapted from the Technology Acceptance Model for Mobile Services (TAMM).[83] The survey consists of 14 items and uses a 5-point Likert scale (‘1’, Strongly disagree, to ‘5’, Strongly agree) to measure ease of adoption, ease of use, values and trust. Two additional items using a 11-point scale (‘0’, Not at all likely, to ‘10’, Extremely likely) assess how likely one would consider using the service in the future or recommend to a friend. Users are asked to rate the toolkit overall out of 10 and an open text box is provided for additional comments.		X			
User experience.	User experience is assessed at 3, 6 and 12 months using the TAMM[83] that assesses ease of adoption, ease of use, value, trust, and general usability using 25 items on a 5-point Likert scale (‘1’, Strongly disagree, to ‘5’, Strongly agree). Users are asked to rate the degree of importance, convenience, enjoyment,			X	X	

	satisfaction, and motivation for 14 components of the Toolkit using a 5 point Likert scale ('1' Not at all, to '5', Very). Two additional items using a 11-point scale ('0', Not at all likely, to '10', Extremely likely) assess how likely one would consider using the service in the future or recommend to a friend. Users are asked to rate the toolkit overall out of 10 and an open text box is provided for additional comments. Three items also assess toolkit usage: (1) 'On average, how frequently do you use the toolkit'; (2) 'What option describes your toolkit use behaviour best?'; and, (3) 'What motivated you to continue/why did you quit using the toolkit?'.					
Meta-data	The data-hub will collect meta-data for individual-level toolkit interactions including engagement and dose, such as the pattern of Toolkit and module use, intensity of use (e.g., timing, frequency of logins and duration of module/sub-module use), usage and content patterns (e.g., percentage of tasks performed and pages visited). Data are analysed using Matlab (MathWorks, Inc., Natick, Massachusetts, USA).		X	X	X	X
