# Supplementary material for: The NoHoW protocol: a multicentre 2×2 factorial randomised controlled trial investigating an evidence-based digital toolkit for weight loss maintenance in European adults
Source: BMJ Open. 2019 Sep 30;9(9):e029425. doi: 10.1136/bmjopen-2019-029425 (PMC6773359; doi:10.1136/bmjopen-2019-029425)
Supplement: Author's manuscript [file bmjopen-2019-029425.draft_revisions.pdf]

BMJ Open is committed to open peer review. As part of this commitment we make the peer review history of every article we publish publicly available.

When an article is published we post the peer reviewers' comments and the authors' responses online. We also post the versions of the paper that were used during peer review. These are the versions that the peer review comments apply to.

The versions of the paper that follow are the versions that were submitted during the peer review process. They are not the versions of record or the final published versions. They should not be cited or distributed as the published version of this manuscript.

BMJ Open is an open access journal and the full, final, typeset and author-corrected version of record of the manuscript is available on our site with no access controls, subscription charges or pay-per-view fees (http://bmjopen.bmj.com).

If you have any questions on BMJ Open's open peer review process please email info.bmjopen@bmj.com

BMJ Open

The NoHoW protocol: A multicenter 2x2 factorial randomized controlled trial investigating an evidence-based digital toolkit for weight loss maintenance in European adults.

Journal:	*BMJ Open*
Manuscript ID	bmjopen-2019-029425
Article Type:	Protocol
Date Submitted by the Author:	25-Jan-2019

Complete List of Authors:	Scott, Sarah; University of Leeds, School of Psychology<br>Duarte, Cristiana; University of Leeds, School of Psychology<br>Encantado, Jorge; Universidade de Lisboa, Faculdade de Motricidade Humana<br>Evans, Elizabeth; Newcastle University, Institute of Health & Society<br>Harjumaa, Marja; VTT Technical Research Centre of Finland<br>Heitmann, Berit; Bispebjerg and Frederiksberg Hospital, The Parker Institut, Research Unit for Dietary Studies; University of Copenhagen, Department of Public Health<br>Horgan, Graham; Biomathematics and Statistics Scotland<br>Larsen, Sofus; Bispebjerg and Frederiksberg Hospital, The Parker Institute, Research Unit for Dietary Studies<br>Marques, Marta; Universidade de Lisboa, Faculdade de Motricidade Humana; University of Dublin Trinity College, Trinity Centre for Practice and Healthcare Innovation & ADAPT Centre<br>Mattila, Elina; VTT Technical Research Centre of Finland<br>Matos, Marcela; Universidade de Coimbra, CINEICC<br>Mikkelsen, Marie-Louise ; Bispebjerg and Frederiksberg Hospital, The Parker Institute, Research Unit for Dietary Studies<br>Palmeira, António; Universidade de Lisboa, Faculdade de Motricidade Humana<br>Pearson, Beth; University of Leeds, School of Psychology<br>Ramsey, Lauren; University of Leeds, School of Psychology<br>Sainsbury, Kirby; Newcastle University, Institute of Health and Society<br>Santos, Inês; Universidade de Lisboa, Faculdade de Motricidade Humana<br>Sniehotta, Falko; Newcastle University, Institute of Health & Society<br>Stalker, Carol; University of Derby, College of Life and Natural Sciences<br>Teixeira, PJ; Universidade de Lisboa, Faculdade de Motricidade Humana<br>Stubbs, James; University of Leeds, School of Psychology

Keywords:	Weight loss maintenance, Digital health, Behaviour change, Self-regulation, Emotion regulation, Motivation

For peer review only - http://bmjopen.bmj.com/site/about/guidelines.xhtml

ABSTRACT

Introduction: Obesity and associated diseases place a severe burden on healthcare systems. Behavioral interventions for weight loss (WL) are successful in the short-term but often result in weight regain over time. Self-regulation of eating and activity behaviors significantly enhance weight loss maintenance (WLM) and may be effectively augmented by contextual-behavioral approaches to emotion regulation. The NoHoW trial tests the efficacy of a theoretically informed, evidence-based digital toolkit using a mobile enabled website, activity trackers and Wi-Fi scales for WLM aiming to target: (i) self-regulation and motivation, and (ii) emotion regulation in adults who achieved clinically significant (≥5%) WL in the previous 12 months (initial BMI ≥25 kg/m^2).

Methods and analysis: The study is an 18 month, 3-center, 2 x 2 factorial single-blind, randomized controlled trial, which recruited 1627 participants achieving ≥5% WL between March 2017 to March 2018. Participants are randomly allocated to one of four arms: (i) self-monitoring only (self-weighing and activity tracker), (ii) self-regulation and motivation, (iii) emotion regulation, or (iv) combined self-regulation, motivation and emotion regulation. Participants attend four clinical investigation days at 0, 6, 12 and 18 months and are instructed to use the digital toolkit for 18 weeks during the first 6 months and at their discretion for the remaining 12 months. The primary outcome is change in weight (kg) at 12 months from baseline. Secondary outcomes are: body composition (e.g., bio-impedance analysis), health biomarkers (HbA1c, lipids, blood pressure, hair cortisol), dietary intake, physical activity, sleep, motivational, self-regulatory, emotion-regulatory moderators/mediators of WLM, engagement, user experience, acceptability and cost-effectiveness of the interventions.

For peer review only

Ethics and dissemination: Ethical approval was granted by institutional ethics committees at the Universities of Leeds (17-0082; 27-Feb-2017), Lisbon (17/2016; 20-Feb-2017) and Capital Region of Denmark (H-16030495, 8-Mar-2017). Results will be published in scientific journals.

Trial registration number: ISRCTN88405328

ARTICLE SUMMARY

Strengths and limitations of this study

- Physical activity and weight are tracked for 18-months using the Fitbit Charge 2™ and Aria™ scales.
- The digital WLM intervention (18 months) is conducted at three European centers in 1627 participants with individualized feedback based on individual tracking measures.
- The 2x2 factorial design is a step forward from a conventional two-armed trial to determine the effect of self-regulation/motivation, emotion regulation interventions and their interactions on weight and health outcomes.
- Participants are blinded to study allocation, but they can access similar interventions from other real-world sources.
- The active control arm includes self-monitoring of physical activity and weight but the inclusion of the Fitbit® devices across all arms potentially contaminates or diminishes the effect of self-regulatory components.

For peer review only - http://bmjopen.bmj.com/site/about/guidelines.xhtml

INTRODUCTION

Overweight, obesity and associated diseases are key societal challenges. Obesity prevalence accounts for 3–8% of health costs and 10–13% of deaths in Europe.[1] This translates into €59 billion a year in direct, and an estimated €118 to €236 billion in indirect, health care costs.[2] These costs are set to rise in parallel with obesity prevalence.[3] Approximately 40% of the adult population in Europe report engaging in at least one weight control attempt in the previous 12 months.[4] Existing community-based programs support initial WL but are subject to high attrition or weight regain, limiting longer-term effectiveness.[5 6] The obesogenic environment and asymmetry of human energy balance (EB) regulation facilitates weight gain, while society criticizes people suffering from overweight and obesity, leading to stress and negative emotions, which can undermine WLM attempts.[7 8] Effective support for WLM or the prevention of weight regain needs to address sustained changes in EB behaviors (dietary, physical activity and weight control) and additional emotional/psychosocial challenges that may undermine planned healthy behaviors.

Self-regulation and motivation

Core features of effective WLM interventions include behavior change techniques in line with self-regulation theories such as: 1) *self-monitoring* of weight and behavior; 2) *goal-setting*: agreement of clear weight targets/trigger points for weight control efforts; 3) feedback on behavior and weight; 4) *action plans* for weight control through dietary and physical activity behaviors and, 5) *plans* to cope with *risk factors* for weight regain and relapse prevention (e.g., problem solving).[9-15] Further, self-determined autonomous motivation has been consistently associated with change in EB behaviors for obesity management.[16 17] Interventions rooted in

For peer review only

Self-Determination Theory may offer a promising approach to sustained EB and weight control.[12 17] A recent systematic review exploring psychological mediators of sustained effects in diet and lifestyle obesity interventions found that higher levels of autonomous motivation, self-efficacy, self-regulation skills, flexible eating restraint, and positive body image were mediators of medium to long-term weight control.[18] The combination of motivational and volitional processes of EB behaviors can contribute to effective WLM; that is, it appears those who are adequately motivated and who implement intentions tend to both initiate and maintain such changes.[19-21] However, physical activity and dietary interventions based on current behavior change theories characteristically achieve significant, but small and heterogeneous effects.[22] Additional psychological processes, such as emotion regulation, can potentially have incremental beneficial effects on the behavioral changes that promote WLM.[23]

Emotion regulation

Individuals with obesity or those trying to lose weight can experience increased stress, depression, anxiety and weight-related stigma[24 25] which may undermine weight control attempts. Repeated attempts at WL followed by weight regain can have a negative emotional impact, leading to self-critical thoughts and negative emotions. For some, ‘comfort eating’ may be a means of coping with these negative experiences, potentially derailing strategies of planned behavior.[26-28] Relapse prevention models include contextual behavioral (CB) based emotion regulation strategies such as compassion, acceptance and mindfulness.[29-31] Evidence suggests that mindfulness and acceptance of undesired internal experiences (e.g., negative emotions or self-criticism) and compassion for the self and others may help some individuals

For peer review only

For peer review only - http://bmjopen.bmj.com/site/about/guidelines.xhtml

adopt adaptive behaviors that are congruent with their weight goals or values.[32-36] Mindfulness-based interventions show promise in changing obesity-related eating behaviors (e.g., binge eating and emotional eating), lowering depression levels and improving WLM.[37] Augmenting current self-regulatory approaches with CB emotion regulatory approaches could lead to more effective WLM.[32-36]

For peer review only

Digital behavior change interventions

Recently, there has been interest in digital interventions to promote and maintain health.[38] Digital solutions, such as smartphone applications and wearables, can be effective in supporting WLM if they are evidence-based, that apply a choice of behavior change techniques with individualized feedback,[22 39 40] encourage the use of self-regulatory techniques (e.g., self-monitoring) and develop intrinsic motivation.[41] Digital solutions are potentially cost-effective and scalable to large populations, which could engage citizens in healthcare innovations that are convenient and effective for weight management in the face of limited public budgets.[42] To date, most commonly available weight management applications miss key evidence-based approaches for longer-term behavior change.[43 44]

Study objectives and hypotheses

The effectiveness of long-term evidence-based digital interventions and strategies to support WLM is limited.[45] The study's primary objectives are to evaluate whether using a new digital toolkit is effective for WLM by improving: (i) self-regulation and motivation, (ii) emotion regulation, or (iii) these factors in combination, compared to control (generic toolkit content,

For peer review only - http://bmjopen.bmj.com/site/about/guidelines.xhtml

109 regular self-weighing and Fitbit® use) in 1627 participants at three European centers. We hypothesize that the combined intervention will be more effective for WLM compared to the self-regulatory or emotion regulatory interventions alone, which will also be more effective than the control group.

113 Secondary objectives are to determine how the intervention affects health markers (e.g., levels of glycated haemoglobin [HbA1c]) and body composition. The study will examine: (i) the intervention impact on physical activity, sleep, dietary intake, depression, anxiety, stress, quality of life and wellbeing; (ii) mediators of WLM, such as self-regulation (e.g., planning capacity), motivation (e.g., autonomous motivation) and emotion regulation processes (e.g., self-compassion) (iii) baseline moderators (e.g., gender, BMI) to identify participants who are more responsive to motivational and behavioral self-regulatory or emotion regulatory approaches for WLM; (iv) quantitative and qualitative assessment of user-experience, acceptability, engagement and drop-out; (v) intervention cost-effectiveness; and (vi) to determine the efficacy of individualized feedback from data on physical activity self-tracking.

METHODS

Trial design and setting

126 The NoHoW trial is a 3-center (University of Leeds [UK], The Parker Institute [Denmark], and University of Lisbon [Portugal]) 2x2 factorial, randomized, single-blind, controlled trial testing the proof-of-concept of a digital toolkit for WLM. The study duration is 18-months with a follow-up at 6, 12 and 18-months post-baseline (Figure 1). The study is conducted between

For peer review only - http://bmjopen.bmj.com/site/about/guidelines.xhtml

March 2017 and September 2019 in academic research institutes. In total, 1627 participants (~542 per center) were enrolled and randomized into one of four intervention arms: (1) active control arm (consisting of generic toolkit content, self-weighing and activity trackers, only); (2) self-regulation and motivation arm; (3) emotion regulation arm; and, (4) combined motivation/self-regulation and emotion regulation arm (Figure 1). The protocol is harmonized across trial centers using Good Clinical Practice (GCP) guidance, research-grade translation/back-translation of trial materials, two training workshops and weekly Trial Management meetings. The trial registration data set is displayed in Table 1. The final protocol (V2.1 20/09/2017) was approved by the Trial Steering Committee (TSC) and adheres to the SPIRIT guidelines.[46]

FIGURE 1 NEAR HERE Legend: **Figure 1.** Trial design

INSERT TABLE 1 HERE

Participants

Table 1 describes inclusion/exclusion criteria for the trial. Prior to randomization, potential participants are asked to provide documented verification (by a health professional, WL counsellor/friend, WL program record booklet, diary or smartphone app or before/after photographs[47]) that they have achieved a clinically significant WL of >5% during the previous 12 months.

Study recruitment

For peer review only

For peer review only - http://bmjopen.bmj.com/site/about/guidelines.xhtml

The study recruitment and intervention schedule is outlined in Table 2. Center-specific recruitment strategies were adopted for 12 months (March 2017- March 2018) and included: commercial WL program (UK, Slimming World); the Copenhagen Municipality weight management services, Dieticians from the Danish Association for Dieticians and commercial slimming companies (e.g., Sense, Henrik Duer and Per Nielsen); registered clinical dieticians/nutritionists who provide weight management services in Lisbon; leisure centers; and local/national media coverage and advertisements. All participants were directed to country-specific recruitment websites (http://uk.nohow.eu; http://dk.nohow.eu; http://nohow.fmh.ulisboa.pt) and completed an online eligibility screener using Qualtrics™. The eligibility screener categorized respondents as eligible, potentially eligible (wait-list) and ineligible. Eligible individuals were contacted for a telephone screening interview, provided with study information, medical history questions and the Physical Activity Readiness Questionnaire.[48] Eligible participants were invited to a Clinical Investigation Day (CID) where informed consent was obtained by research staff before randomization (Supplementary file 1). Participants are re-consented before each subsequent visit and excluded if they become ineligible.

Public involvement

Members of the public that matched the study's inclusion criteria were consulted to inform the recruitment strategies, trial conduct, questionnaires, assessment of burden and reviewing or testing the intervention content at all trial centers.

***INSERT TABLE 2 HERE ***

Randomization and blinding

For peer review only - http://bmjopen.bmj.com/site/about/guidelines.xhtml

Participants were randomly allocated to treatment arms by researchers using an online trial administration portal. Adaptive stratified sampling using minimisation[49] was embedded in the portal using the R-program (R Foundation for Statistical Computing, Vienna, Austria) by the study statistician GH. This minimizes differences in age, weight lost in the previous 12 months and current BMI between treatment arms and stratified by gender and center. Participants were blinded to study allocation. The research team were not blinded due to the need to train participants in arm-specific toolkit versions. The statistician (GH) is independent from data collection but not blinded during analyses due to inclusion of the arm allocation number. Independent researchers analyzing hair cortisol will be blinded to group allocation. No preliminary or ancillary analysis of outcome data between intervention arms will be conducted prior to data-lock (12 months).

Interventions

The toolkit is a mobile-enabled website, delivered as a portfolio of embedded tracking technologies, mini-apps, and web resources (Figure 2) accessible via computer, tablet and mobile phone. The tracking technologies consist of an activity and sleep tracker (Fitbit Charge 2™) and smart wireless body weight scale (Fitbit Aria™). Participants were asked to wear the Fitbit Charge 2 ™ at all times and to weigh themselves ≥ twice weekly by research staff. The logic models for each arm will be presented in a subsequent publication outlining the toolkit development and theoretical content. All arms include instructions for self-weighing and use of activity trackers. Arms 2 and 4 includes individualized weekly feedback about weight trajectories. Guidance for complex interventions was followed during the toolkit development specifying the intervention logic models and theory-driven behavior change techniques,[50] which will be detailed in a separate publication.

For peer review only

For peer review only - http://bmjopen.bmj.com/site/about/guidelines.xhtml

FIGURE 2 HERE Legend: **Figure 2**. Toolkit example (arm 4).

Arm 1 (Active control, self-monitoring, only)

The active control arm includes a toolkit dashboard containing weight, steps and sleep graphs using data from the Fitbit® devices, healthy eating and mood data (self-scored on a 5-point scale), a diary to populate with notes from these five tiles, and a help tile. The content is generic and does not relate to any theoretically informed, evidence-based behavior change approaches.

Arms 2-4.

After randomization, participants received standardized training from researchers during the first CID, specific to their arm allocation. Trial arms 2-4 include the same components as arm 1 but participants also receive a personal virtual road map (the ‘NoHoW Journey’) with intermediate destinations represented in that map by circles, each representing evidence-based theoretically-informed thematic modules targeting self-regulation and motivation (arm 2), emotion regulation (arm 3), and combined self-regulation and emotion regulation (arm 4). Arm 4 includes the same number of thematic modules as arms 2 and 3. Each weekly module opens into sub-modules. The active intervention involves 18 weekly sessions delivered throughout a six-month period. Users are encouraged to complete ≥1 sub-module per week following the sequence in the road map, consisting of videos/audios, interactive graphs, text, images, questionnaires and mini-apps. Weekly arm-specific emails are sent during the active six-month intervention phase, which provide WLM information, suggestions, and reminders to visit specific content. The toolkit automatically sends an email notifying participants to slow down if they complete three sessions ahead of schedule. After six months, they are encouraged to re-visit the toolkit for a further 12 months at their discretion.

For peer review only

Individualised feedback is a component of the self-regulation and motivation arms (arms 2 and 4) automatically provided by the toolkit in response to the individual's weight trajectory. Feedback is generated from the data extracted from three sources: (1) weight from the Fitbit Aria™ scales; (2) activity and sleep from the Fitbit Charge 2™; and, (3) user engagement with the toolkit, including day of the week. After daily usage patterns are analysed, feedback is displayed in the toolkit consisting of one of a set of short statements (e.g., "your weight management appears better when you are more active").

Adherence

The toolkit sends automated weekly emails to participants to encourage engagement, prompt log-in, and recommends sessions to complete (arms 2-4 only). The control group receives the same number of emails but with links to generic content (e.g., topical diet/health information). Intervention adherence is continuously monitored by collecting information on the number of days since users last logged into the toolkit and the number of days since the last synchronization between the Fitbit® devices and data-hub. If no activity is detected after 21, 28 and 35 days, three attempts are made to contact participants by email. After 42 days of no activity, researchers telephone participants to resolve issues. Reasons for no activity are recorded.

Assessments

The primary outcome is change in weight (kg) between baseline and 12 months (primary end-point). Follow-up measures for WLM are completed at month 18 for comparability with other longitudinal studies.[22] All physical measurements are taken after a 10-12 hour overnight fast by trained research staff. Measures are taken at all four time points (baseline, 6, 12 and 18

For peer review only - http://bmjopen.bmj.com/site/about/guidelines.xhtml

For peer review only

months) except HbA1c, lipids and hair cortisol (baseline and 12 months only) and process evaluation measures (1, 3, 6 and 12 months only). The study schedule and information for all measures is in supplementary file 2.

Participant retention

Randomized participants are contacted at least two weeks before each visit and sent an email/text reminder. If participants deviate from planned assessments, three attempts are made to contact them by phone, email and a final written letter across one month. At least weight (primary outcome) is obtained if possible.

Primary outcome

Weight

Body weight (±0.1kg) is measured using a Seca 704s instrument (SECA, Germany) in participants wearing light clothing. Participants are asked to weigh themselves at least twice weekly, in the morning after voiding and before eating using the Fitbit Aria™ scales for 18 months.

Secondary outcome measures

Body composition and height

For peer review only

Multi-frequency whole Bioimpedance spectroscopy (BIS) is measured by ImpediMed™ SFB7, Queensland, Australia), which measure impedance over a spectrum of frequencies for the estimation of body composition. Hanai mixture theory equations and standard resistivity constants[51] are used to calculate fluid volumes to estimate total body water. Height (±0.1cm) is measured with participants barefoot, using a Seca 704s instrument (SECA, Germany). See supplementary file 2.

Waist Hip Ratio

Waist and hip measurements are taken according to the World Health Organization's (WHO) guidance. A tape measure is used to record the hip and waist circumference to the nearest centimeter.[52] The waist-hip ratio is calculated by dividing hip and waist circumference.

Biomarkers

HbA1c and Cholesterol

Fasted capillary blood samples are collected to determine HbA1c (mmol/mol, %), estimated average glucose (eAG, mol/L) and full lipid profiles, including total cholesterol, low density lipoprotein (LDL), high density lipoprotein (HDL), triglycerides, non-HDL and cholesterol/HDL (mmol/L) assayed using a bench-top analyzer (Alere Afinion™ AS100 Analyzer, Alere, Stockport, UK).[53]

For peer review only - http://bmjopen.bmj.com/site/about/guidelines.xhtml

278 *Blood Pressure heart rate*

279 Systolic and diastolic blood pressure (BP) and resting heart rate (HR) (Microlife BP A2 Basic,
280 Gentle Technology, Microlife, Clearwater, FL, USA, Inc.) are measured three times with the
281 participant at rest in the sitting position. The mean value is recorded.

282

283 *Hair Cortisol*

284 Hair samples are collected by cutting a small tuft of hair close to the scalp to measure the stress
285 hormone, cortisol and analyzed following a previously described protocol[54] (supplementary
286 file 2).

287

288 *Physical activity*

289 Physical activity is measured by the self-reported International Physical Activity Questionnaire
290 (IPAQ)[55] and Activity Choice Index (ACI).[56] Minute-by-minute physical activity data and
291 heart rate are measured by the Fitbit Charge 2™ for the study duration.

292

293 *Sleep quality and quantity*

294 The Fitbit Charge 2™ estimates sleep quantity (hours/minutes) and quality (stages of sleep).
295 Data are collected throughout the trial.

For peer review only - http://bmjopen.bmj.com/site/about/guidelines.xhtml

Dietary intake and eating behavior

Four consecutive 24-hour-web-based dietary recalls, including at least one weekend day, are collected within seven days of each visit using INTAKE 24.[57] Psychometric measures of eating behavior are assessed at each visit using the Three Factor Eating Questionnaire-51,[58] the Problematic Eating Patterns Scale (unpublished scale), Eating in the Absence of Hunger Scale,[59] Intuitive Eating Scale-2[60] and the Binge Eating Scale.[61]

Well-being and Quality of Life

Quality of life and wellbeing are assessed using the EQ5D-5L[62] and the Warwick-Edinburgh Well-Being Scale[63] respectively.

Self-regulation

Self-regulation is measured using the Action Planning and Coping Planning Scales,[64] and Action Control Scale.[65]

Motivation

Satisfaction of psychological needs are assessed using the Basic Psychological Needs and Frustrations Scale.[66] Quality of goal contents is assessed using the Goal Content for Weight Loss Maintenance Scale.[67 68] Autonomous and controlled motivation are assessed using the

For peer review only

For peer review only - http://bmjopen.bmj.com/site/about/guidelines.xhtml

315 Behavioral Regulation in Exercise Questionnaire (BREQ-3),[69] Regulation of Eating Behavior Scale (REBS),[70] and Regulations for Weight Management Scale (adapted from the REBS).[70]

317 *Self-efficacy*

318 Self-efficacy is assessed using the Self-Efficacy for Weight Maintenance Scale.[71]

319

320 *Emotion regulation and stress management*

321 CB-emotion regulation and stress management constructs are measured using the Weight Focused Self-Criticism/Self-Reassurance Scale,[72] Weight Focused External Shame Scale,[73 74] the Compassion Attributes and Actions Scales,[75] Body Image Acceptance and Action Questionnaire,[76] Engaged Living Scale,[77] Mindful Attention Awareness Scale,[78] adapted Difficulties in Emotion Regulation Scale,[79] and Experiences Scales (decentering sub-scale only).[80] Perceived stress is measured by the Perceived Stress Scale.[81] Anxiety and depression are measured using the Depression Anxiety Stress Scale.[82]

328

329 Demographics, weight history and participant traits.

330 A profiling questionnaire, adapted from the DiOGenes study,[83] is administered at baseline only to measure trait-specific moderators of WLM including demographics: age, gender, country, education, marital status, employment, and income. Also, smoking and alcohol use and number of times giving birth are recorded. Weight history includes: height, current and highest weight in the last 12 months, successful and unsuccessful WL attempts and strategies, and

For peer review only - http://bmjopen.bmj.com/site/about/guidelines.xhtml

psychological impact of living in a food abundant environment (the Power of Food Scale[84]). Self-determination traits are assessed using the Index of Autonomy Functioning[85] and Aspirations Index,[86] and self-regulation of appetite, hunger and satiety cues by the Intuitive Eating Scale-1.[87] Relapse prevention capability is measured using an adapted Relapse Prevention Scale.[88]

Process evaluation and user experience

Questionnaires adapted from the Technology Acceptance Model for Mobile Services (TAMM)[89] are administered by email to assess reasons for engagement/drop-out and user-experience (e.g., perceived ease-of-use and adoption, perceived value, trust, acceptability, and usability). Digital literacy is assessed at one month using the eHealth Literacy Scale (eHEALS).[90] Twelve focus groups are conducted (four in each country) at six months, by independent researchers, to thematically explore contextual factors (e.g., influence of wider social networks), user experience, and the toolkit use context in the three countries.[91] Meta-data on reach (participant characteristics), dose, fidelity and engagement with the toolkit is extracted from log files (Supplementary file 2). Methods and analyses with be detailed in separate publications.

Data management

A NoHoW data-hub was developed by the James Hutton Institute (Edinburgh) and led by GH, which collates, monitors and stores anonymized data securely from each center. Data from

For peer review only

each center is entered into trial management software (Easy Trial: www.easytrial.net). Quality checks are performed by all centers and reviewed during weekly Trial Management meetings. Separate publications will detail: (i) the technologies to host the toolkit, and (ii) the ICT architecture linking the toolkit, trial administration portal, trial management software and NoHoW data-hub.

Data Analysis

The 2 × 2 factorial design will be used in a model to investigate links between outcomes, and the extent of mediation, or moderation by covariates. For continuous outcomes, linear regression models will be used. The effects of different treatment arms will be presented with 95% confidence intervals of coefficients and p-values. For analyses that do not meet model assumptions, outcomes will be transformed prior to inferential comparisons. For categorical outcomes, logistic regression or categorical response models such as ordinal or multinomial regression will be used. Treatment effects will be presented as odd ratios, 95% confidence intervals, and p-values. Missing values will be imputed using multiple as part of an intention to treat (ITT) analysis.

To determine the mechanisms that underlie intervention efficacy, linear models are used to test whether the treatment (independent variable) causes a change in the potential mechanisms of change (mediators), which in turn, cause a change in the primary outcomes (dependent variables). Models also investigate the effects of covariates that may moderate the effect of the interventions. Mediation is assessed where change in weight, as represented by a categorical end-point (maintenance vs. weight regain) is the dependent variable, intervention

For peer review only

For peer review only - http://bmjopen.bmj.com/site/about/guidelines.xhtml

group is the predictor variable, and self-regulatory, emotion regulatory and other psychosocial variables are mediators. Hybrid models, combining latent and observed variables, may be specified to ensure sufficient power to conduct the analysis and to stay within the limits of sample size requirements. Model fit will be assessed using the Chi-square goodness-of-fit test, Bollen's incremental fit index (IFI 0.95), and the standardized root mean residual (SRMR G 0.08) values. Tests of mediation will be conducted using a resampling method. To test the significance of specific indirect effects of multiple mediators (total and specific for each mediator, using both normal theory and bootstrap procedures), a tool developed by Selig and Preacher[92] will be used.

For peer review only

Subgroup Analysis

Interactions between condition factors and baseline participant groupings (center, gender, age, BMI at entry, % WL at entry) will be prioritized as *a priori* sub group analyses in the primary and secondary outcomes. These pre-planned analyses will be conducted by the inclusion of appropriate interaction terms in the regression models. Where interactions are significant, results for each subgroup will be reported. This will mitigate the problems of multiple testing due to many subgroup comparisons. We recognize that statistical power in subgroups will vary with sample size and provide a plot of detectable effect size versus sample size for reference (Figure 3).

FIGURE 3 NEAR HERE Legend: **Figure 3**. Plot of detectable effect size (Cohen's D)

Cost-effectiveness analyses

399 Cost-effectiveness analysis will be conducted to model costs and intervention effects for a
400 lifetime horizon. An existing decision-analytic model will be used to estimate BMI category
401 transitions, incidence of secondary diseases (e.g., Type II diabetes), quality of life and
402 healthcare costs.[93] Quality-adjusted life-years (QALYs) and costs will allow calculation of
403 Incremental Cost-effectiveness Ratios (ICERs) and estimates of cost-utility. Extensive
404 sensitivity analyses will test the modelling assumptions. Exploration of scenarios relating *inter*
405 *alia* to the cost and effects (including sustainability) of the intervention. Secondary cost-
406 effectiveness analysis will explore WLM outcomes (e.g., cost per Kg maintained per year).
407 The model will be adapted separately for each country.

408

409 Power calculations and sample size estimation

410 Power calculations were based on weight and health markers (HbA1c). To detect a difference
411 between treatment arms of >1.5kg body weight gives a (Cohen's) d value of 0.25.[22] For 80%
412 power, comparing >two groups requires a sample size of 250 per trial arm. To detect an effect
413 size of 0.25 SD units for HbA1c, 245 participants in each trial arm gives 80% power, at 5%
414 significance. Assuming 38% drop out,[83] a sample size of 1600 (533 per center) is needed to
415 achieve a sample of 1002 (334 per center, ~250 per trial arm) participants at 12 months.

416

417 **Ethical considerations and dissemination plans**

418 The trial was registered with the ISRCTN registry (ISRCTN88405328). Ethical approval was
419 granted by each institutional ethics committee before study commencement at each center. The

For peer review only - http://bmjopen.bmj.com/site/about/guidelines.xhtml

protocol complies with relevant EC legislation, international conventions and declarations relating to ethical research practices.[94] Interventions will be discontinued if they are reported detrimental. Protocol deviations, violations, and serious adverse events or adverse events are recorded by trial staff and monitored by the Principal Investigator (JS) and TSC.[95] Any protocol amendments that impact the study conduct are approved by each institutional ethics committee and communicated to participants. Trial results will be published in peer-reviewed scientific journals. Baseline data will be analyzed after the end of recruitment (March 2018) and outcome analyses after one-year data is collected (March 2019). Authorship will be determined by the guidelines set out by the International Committee of Medical Journal Editors.

Acknowledgements

The authors thank David Meads, Marlene Silva, Cláudia Ferreira, Heather Patrick, Ian Caterson, John Blundell, all members of the NoHoW Consortium and the independent members of the NoHoW trial steering committee (Sharon Simpson, Paul Aveyard, Lucy Yardley and Annette Kjær Ersbøll).

Author Contributions: RJS, BLH, PT, FFS, ALP and GH conceived the study; RJS is the principle investigator. BLH is the grant coordinator and SES is the trial manager. SCL, ALP and SES are site coordinators. GH is the trial statistician and leads data management. SES and RJS drafted the manuscript and all authors revised and approved the final version. All authors made substantial contributions to the conceptualization of the study design and conduct of the protocol

Disclosure of funding: This project has received funding from the European Union's Horizon 2020 research and innovation programme under grant agreement No 643309. The material

For peer review only - http://bmjopen.bmj.com/site/about/guidelines.xhtml

presented and views expressed here are the responsibility of the author(s) only. The EU Commission takes no responsibility for any use made of the information set out. MMM is funded by the Marie-Sklodowska-Curie (EDGE) Fellowship programme [grant agreement No. 713567]

Competing interests: RJS consults for Slimming World through the University of Leeds. MMM and GH also conducted consultancy work with Slimming World. Slimming World were a partner of the project to aid recruitment within the UK but will not be involved in the analysis and interpretation of the trial.

Provenance and peer review: Not commissioned; externally peer-reviewed.

Data sharing statement: Subject to intellectual property (IP) negotiations, data will be made available in an open access data repository.

REFERENCES

1. Hunt A, Ferguson J. Health costs in the European Union: how much is related to EDCS. Brussels, The Health and Environmental Alliance, 2014. https://www.env-health.org/IMG/pdf/18062014_final_health_costs_in_the_european_union_how_much_is_realted_to_edcs.pdf (accessed 04 Jan 2019).

2. World Health Organization. The challenge of obesity in the WHO European region and the strategies for response. Copenhagen, Denmark Europe, 2007.

For peer review only

http://www.euro.who.int/__data/assets/pdf_file/0008/98243/E89858.pdf (accessed 04 Jan 2019)

3. Wang YC, McPherson K, Marsh T, et al. Health and economic burden of the projected obesity trends in the USA and the UK. Lancet 2011;378(9793):815-25.

4. Santos I, Sniehotta F, Marques M, et al. Prevalence of personal weight control attempts in adults: a systematic review and meta-analysis. Obes Rev 2017;18(1):32-50.

5. Franz MJ, VanWormer JJ, Crain AL, et al. Weight-loss outcomes: a systematic review and meta-analysis of weight-loss clinical trials with a minimum 1-year follow-up. J Am Diet Assoc 2007;107(10):1755-67.

6. Brownell KD, Marlatt GA, Lichtenstein E, et al. Understanding and preventing relapse. Am Psychol 1986;41:765-82.

7. Stubbs RJ, Gail C, Whybrow S, et al. The evolutionary inevitability of obesity in modern society: implications for behavioral solutions to weight control in the general population. In: Martinez MP, Robinson H, eds. Obesity and Weight Management: Challenges, Practices and Health Implications. New York: Novo Publishers 2012.

8. Sainsbury K, Evans EH, Pedersen S, et al. Attribution of weight regain to emotional reasons amongst European adults with overweight and obesity who regained weight following a weight loss attempt. Eat Weight Disord 2018;1-11 doi: 10.1007/s40519-018-0487-0 [published Online First: 16 February 2018]

For peer review only

For peer review only - http://bmjopen.bmj.com/site/about/guidelines.xhtml

9. Stubbs RJ, Lavin JH. The challenges of implementing behaviour changes that lead to sustained weight management. Nutr Bull 2013;38:5-22.

10. Dombrowski SU, Avenell A, Sniehott FF. Behavioural interventions for obese adults with additional risk factors for morbidity: systematic review of effects on behaviour, weight and disease risk factors. Obes Facts 2010;3(6):377-96.

11. Michie S, Abraham C, Whittington C, et al. Effective techniques in healthy eating and physical activity interventions: a meta-regression. Health Psychol 2009;28(6):690-701.

12. Teixeira PJ, Going SB, Sardinha LB, et al. A review of psychosocial pre-treatmant predictors of weight control. Obes Rev 2005;6:43-65.

13. Silva MN, Markland D, Carraca EV, et al. Exercise autonomous motivation predicts 3-yr weight loss in women. Med Sci Sports Exerc 2011;43(4):728-37.

14. Kwasnicka D., Presseau J., White M., et al. Does planning how to cope with anticipated barriers facilitate health-related behavior change? A systematic review. Health Psychol Rev 2013;7:129-45.

15. Dombrowski SU, Sniehotta FF, Avenell A, et al. Identifying active ingredients in complex behavioural interventions for obese adults with obesity-related co-morbidities or additional risk factors for co-morbidities: a systematic review. Health Psychol Rev 2012;6(1):7-32.

16. Silva MN, Markland D, Carraça EV, et al. Using self-determination theory to promote physical activity and weight control: A randomized controlled trial in women. J Behav Med 2011;33:110-22.

For peer review only

For peer review only - http://bmjopen.bmj.com/site/about/guidelines.xhtml

17. Teixeira PJ, Mata J, Williams GC, et al. Self-regulation, motivation, and psychosocial factors in weight management. J Obes 2012;2012: 582348.

18. Teixeira PJ, Carraça EV, Marques MM, et al. Successful behavior change in obesity interventions in adults: a systematic review of self-regulation mediators. BMC Med 2015;13(1):84.

19. Hagger MS, Chatzisarantis NL. An integrated behavior change model for physical activity. Exerc Sport Sci Rev 2014;42(2):62-69.

20. Teixeira PJ, Silva MN, Mata J, et al. Motivation, self-determination, and long-term weight control. Int J Behav Nutr Phys Act 2012;9:22.

21. Verstuyf J, Patrick H, Vansteenkiste M, et al. Motivational dynamics of eating regulation: a self-determination theory perspective. Int J Behav Nutr Phys Act 2012;9(1):21.

22. Dombrowski SU, Knittle K, Avenell A, et al. Long term maintenance of weight loss with non-surgical interventions in obese adults: systematic review and meta-analyses of randomised controlled trials. BMJ 2014;348:g2646.

23. O'Reilly GA, Cook L, Spruijt-Metz D, et al. Mindfulness-based interventions for obesity-related eating behaviours: a literature review. Obes Rev 2014;15(6):453-61.

24. Dallman MF, Pecoraro N, Akana SF, et al. Chronic stress and obesity: a new view of "comfort food". Proc Natl Acad Sci U S A 2003;100(20):11696-701.

25. Block JP, He Y, Zaslavsky AM, et al. Psychosocial stress and change in weight among US adults. Am J Epidemiol 2009;170(2):181-92.

For peer review only - http://bmjopen.bmj.com/site/about/guidelines.xhtml

26. Gibson EL. The psychobiology of comfort eating: implications for neuropharmacological interventions. Behav Pharmacol 2012;23(5-6):442-60.

27. Lebel JL, Lu J, Dube L. Weakened biological signals: highly-developed eating schemas amongst women are associated with maladaptive patterns of comfort food consumption. Physiol Behav 2008;94(3):384-92.

28. Pedersen S, Sniehotta FF, Sainsbury K, et al. The complexity of self-regulating food intake in weight loss maintenance. A qualitative study among short-and long-term weight loss maintainers. Soc Sci Med 2018;208:18-24.

29. Hendershot CS, Witkiewitz K, George WH, et al. Relapse prevention for addictive behaviors. Subst Abuse Treat Prev Policy 2011;6:17.

30. Bowen S, Chawla N, Witkiewitz K. Mindfulness-based relapse prevention for addictive behaviors. In: Baer RA, ed. Mindfulness-Based Treatment Approaches: A Clinician's Guide. 2nd ed. San Diego, CA: Elsevier Academic Press. 2014:141-57.

31. Duarte C, Stubbs J, Pinto-Gouveia J, et al. The impact of self-criticism and self-reassurance on weight-related affect and well-being in participants of a commercial weight management programme. Obes Facts 2017;10(2):65-75.

32. Lillis J, Hayes SC, Bunting K, et al. Teaching acceptance and mindfulness to improve the lives of the obese: A preliminary test of a theoretical model. Ann Behav 2009;37:58-69.

33. Forman EM, Butryn ML, Hoffman KL, et al. An open trial of an acceptance-based behavioral intervention for weight loss. Cogn Behav Pract 2008;16:223-35.

For peer review only

For peer review only - http://bmjopen.bmj.com/site/about/guidelines.xhtml

34. Forman EM, Butryn ML, Juarascio AS, et al. The mind your health project: a randomized controlled trial of an innovative behavioral treatment for obesity. Obesity 2013;21(6):1119-26.

35. Niemeier HM, Leahey T, Palm Reed K, et al. An acceptance-based behavioral intervention for weight loss: a pilot study. Behav Ther 2012;43(2):427-35.

36. Tapper K, Shaw C, Ilsley J, et al. Exploratory randomised controlled trial of a mindfulness-based weight loss intervention for women. Appetite 2009;52(2):396-404.

37. Ohsiek S, Williams M. Psychological factors influencing weight loss maintenance: an integrative literature review. J Am Assoc Nurse Pract 2011;23(11):592-601.

38. Yardley L, Choudhury T, Patrick K, et al. Current issues and future directions for research into digital behavior change interventions. Am Journal Prev Med 2016;51(5):814-15.

39. MacLean PS, Wing RR, Davidson T, et al. NIH working group report: innovative research to improve maintenance of weight loss. Obesity 2015;23(1):7-15.

40. Evans EH, Araújo-Soares V, Adamson A, et al. The NULevel trial of a scalable, technology-assisted weight loss maintenance intervention for obese adults after clinically significant weight loss: study protocol for a randomised controlled trial. Trials 2015;16(1):421.

41. Lentferink AJ, Oldenhuis HK, de Groot M, et al. Key Components in eHealth Interventions Combining Self-Tracking and Persuasive eCoaching to Promote a Healthier Lifestyle: A Scoping Review. J Med Internet Res 2017;19(8).

For peer review only

For peer review only - http://bmjopen.bmj.com/site/about/guidelines.xhtml

42. European Commission. eHealth Action Plan 2012-2020. 2012. http://ec.europa.eu/digital-agenda/en/news/ehealth-action-plan-2012-2020-innovative-healthcare-21st-century. (accessed 4 Jan 2019).

43. Pagoto S, Schneider K, Jojic M, et al. Evidence-based strategies in weight-loss mobile apps. Am Journal Prev Med 2013;45(5):576-82.

44. Breton ER, Fuemmeler BF, Abroms LC. Weight loss—there is an app for that! But does it adhere to evidence-informed practices? Transl Behav Med 2011;1(4):523-29.

45. Hutchesson M, Rollo M, Krukowski R, et al. eHealth interventions for the prevention and treatment of overweight and obesity in adults: a systematic review with meta-analysis. Obes Rev 2015;16(5):376-92.

46. Chan A-W, Tetzlaff JM, Altman DG, et al. SPIRIT 2013 statement: defining standard protocol items for clinical trials. Ann Intern Med 2013;158(3):200-07.

47. Klem ML, Wing RR, McGuire MT, et al. A descriptive study of individuals successful at long-term maintenance of substantial weight loss. Am J Clin Nutr 1997;66(2):239-46.

48. Thomas S, Reading J, Shephard RJ. Revision of the physical activity readiness questionnaire (PAR-Q). Can J Sport Sci 1992;17(4):338-45.

49. Altman DG, Bland JM. Treatment allocation by minimisation. BMJ 2005;330(7495):843.

50. Craig P, Dieppe P, Macintyre S, et al. Developing and evaluating complex interventions: the new Medical Research Council guidance. Int J Nurs Stud 2013;50(5):587-92.

For peer review only

577 51. De Lorenzo A, Andreoli A, Matthie J, et al. Predicting body cell mass with bioimpedance
578 by using theoretical methods: a technological review. J Appl Physiol 1997;82(5):1542-58.

579 52. World Health Organisation. Guide to physical measurements (Step 2) 2008.
580 https://www.who.int/ncds/surveillance/steps/Section%204%20Step%202%20Physical%20M
581 easurements.pdf (accessed 4 Jan 2019).

582 53. Jain A, Rao N, Sharifi M, et al. Evaluation of the point of care Afinion AS100 analyser in
583 a community setting. Ann Clin Biochem 2017;54(3):331-41.

584 54. Van Uum S, Sauve B, Fraser L, et al. Elevated content of cortisol in hair of patients with
585 severe chronic pain: a novel biomarker for stress. Stress 2008;11(6):483-88.

586 55. Craig CL, Marshall AL, Sjorstrom M, et al. International physical activity questionnaire:
587 12-country reliability and validity. Med Sci Sports Exerc 2003;35(8):1381-95.

588 56. Mullen SP, Silva MN, Sardinha LB, et al. Initial validation of the Activity Choice Index
589 among overweight women. Res Q Exer Sport 2016;87(2):174-81.

590 57. Simpson E, Bradley J, Poliakov I, et al. Iterative Development of an online dietary recall
591 tool: INTAKE24. Nutrients 2017;9(2):118.

592 58. Stunkard AJ, Messick S. The three-factor eating questionnaire to measure dietary restraint,
593 disinhibition and hunger. J Psychosom Res1985;29(1):71-83.

594 59. Arnold TA, Johnston CS, Lee CD, et al. Eating in the absence of hunger in college students.
595 Appetite 2015;92:51-56.

For peer review only - http://bmjopen.bmj.com/site/about/guidelines.xhtml

60. Tylka TL, Kroon Van Diest AM. The Intuitive Eating Scale–2: Item refinement and psychometric evaluation with college women and men. J Couns Psychol 2013;60(1):137.

61. Gormally J, Black S, Daston S, et al. The assessment of binge eating severity among obese persons. Addict Behav 1982;7(1):47-55.

62. Herdman M, Gudex C, Lloyd A, et al. Development and preliminary testing of the new five-level version of EQ-5D (EQ-5D-5L). Qual Life Res 2011;20(10):1727-36.

63. Tennant R, Hiller L, Fishwick R, et al. The Warwick-Edinburgh mental well-being scale (WEMWBS): development and UK validation. Health Qual Life Outcomes 2007;5(1):63.

64. Sniehotta FF, Schwarzer R, Scholz U, et al. Action planning and coping planning for long-term lifestyle change: theory and assessment. Eur J Soc Psychol 2005;35(4):565-76.

65. Sniehotta FF, Scholz U, Schwarzer R. Bridging the intention–behaviour gap: Planning, self-efficacy, and action control in the adoption and maintenance of physical exercise. Psychol Health 2005;20(2):143-60.

66. Ng JY, Ntoumanis N, Thøgersen-Ntoumani C, et al. Predicting psychological needs and well-being of individuals engaging in weight management: The role of important others. Appl Psychol Health Well Being 2013;5(3):291-310.

67. Sebire SJ, Standage M, Vansteenkiste M. Development and validation of the goal content for exercise questionnaire. J Sport Exer Psychol 2008;30(4):353-77.

For peer review only

For peer review only - http://bmjopen.bmj.com/site/about/guidelines.xhtml

614 68. Markland D, Ingledew DK. The measurement of exercise motives: Factorial validity and invariance across gender of a revised Exercise Motivations Inventory. Br J Health Psychol 1997;2(4):361-76.

617 69. Markland D, Tobin V. A modification to the behavioural regulation in exercise questionnaire to include an assessment of amotivation. J Sport Exer Psychol 2004;26(2):191-96.

620 70. Pelletier LG, Dion SC, Slovinec-D'Angelo M, et al. Why do you regulate what you eat? Relationships between forms of regulation, eating behaviors, sustained dietary behavior change, and psychological adjustment. Motiv Emot 2004;28(3):245-77.

623 71. Palmeira AL, Teixeira PJ, Branco TL, et al. Predicting short-term weight loss using four leading health behavior change theories. Int J Behav Nutr Phys Act 2007;4(1):14.

625 72. Gilbert P, Clarke M, Hempel S, et al. Criticizing and reassuring oneself: An exploration of forms, styles and reasons in female students. Br J ClinPsychol 2004;43(1):31-50.

627 73. Goss K, Gilbert P, Allan S. An exploration of shame measures—I: The other as Shamer scale. Pers Indiv Differ 1994;17(5):713-17.

629 74. Allan S, Gilbert P, Goss K. An exploration of shame measures—II: Psychopathology. Pers Indiv Differ 1994;17(5):719-22.

631 75. Gilbert P, Catarino F, Duarte C, et al. The development of compassionate engagement and action scales for self and others. J Compassionate Health Care 2017;4(1):4.

For peer review only

For peer review only - http://bmjopen.bmj.com/site/about/guidelines.xhtml

633 76. Sandoz EK, Wilson KG, Merwin RM, et al. Assessment of body image flexibility: the body
634 image-acceptance and action questionnaire. J Contextual Behav Sci 2013;2(1-2):39-48.

635 77. Trompetter HR, Ten Klooster PM, Schreurs KM, et al. Measuring values and committed
636 action with the Engaged Living Scale (ELS): Psychometric evaluation in a nonclinical sample
637 and a chronic pain sample. Psychol Assess 2013;25(4):1235.

638 78. Carlson LE, Brown KW. Validation of the Mindful Attention Awareness Scale in a cancer
639 population. J Psychosom Res 2005;58(1):29-33.

640 79. Gratz KL, Roemer L. Multidimensional assessment of emotion regulation and
641 dysregulation: Development, factor structure, and initial validation of the difficulties in
642 emotion regulation scale. J Psychopathol Behav Assess 2004;26(1):41-54.

643 80. Fresco DM, Moore MT, van Dulmen MH, et al. Initial psychometric properties of the
644 experiences questionnaire: validation of a self-report measure of decentering. Behav Ther
645 2007;38(3):234-46.

646 81. Cohen S, Kamarck T, Mermelstein R. A global measure of perceived stress. J Health Soc
647 Behav 1983;24(4):385-96.

648 82. Antony MM, Bieling PJ, Cox BJ, et al. Psychometric properties of the 42-item and 21-item
649 versions of the Depression Anxiety Stress Scales in clinical groups and a community sample.
650 Psychol Assess 1998;10(2):176.

For peer review only

For peer review only - http://bmjopen.bmj.com/site/about/guidelines.xhtml

83. Larsen TM, Dalskov S, Van Baak M, et al. The Diet, Obesity and Genes (Diogenes) Dietary Study in eight European countries–a comprehensive design for long-term intervention. Obes Rev 2010;11(1):76-91.

84. Cappelleri JC, Bushmakin AG, Gerber RA, et al. Evaluating the Power of Food Scale in obese subjects and a general sample of individuals: development and measurement properties. Int J Obes 2009;33(8):913.

85. Weinstein N, Przybylski AK, Ryan RM. The index of autonomous functioning: Development of a scale of human autonomy. J Res Pers 2012;46(4):397-413.

86. Grouzet FM, Kasser T, Ahuvia A, et al. The structure of goal contents across 15 cultures. J Pers Soc Psychol 2005;89(5):800.

87. Tylka TL. Development and psychometric evaluation of a measure of intuitive eating. J Couns Psychol 2006;53(2):226.

88. Fuglestad PT, Rothman AJ, Jeffery RW, et al. Regulatory focus, proximity to goal weight, and weight loss maintenance. Am J Health Behav 2015;39(5):709-20.

89. Kaasinen E, Mattila E, Lammi H, et al. Technology acceptance model for mobile services as a design framework. In:J. Lumsden ed. Human-Computer Interaction and Innovation in Handheld, Mobile and Wearable Technologies. Hershey: IGI Global 2011:80-107.

90. Nguyen J, Moorhouse M, Curbow B, et al. Construct validity of the eHealth literacy scale (eHEALS) among two adult populations: a Rasch analysis. JMIR Public Health Surveill 2016;2(1).

For peer review only - http://bmjopen.bmj.com/site/about/guidelines.xhtml

91. Ritchie J, Spencer L, O'Connor W. Carrying out qualitative analysis. In: Richie J, Lewis J, eds. Qualitative Research Practice. London: Sage Publications 2004:219-62.

92. Preacher KJ, & Selig, J. P. Monte Carlo method for assessing multilevel Mediation: An interactive tool for creating confidence intervals for indirect effects in 1-1-1 multilevel models [Computer software]. Available from http://quantpsy.org/.

93. Meads DM, Hulme CT, Hall P, et al. The cost-effectiveness of primary care referral to a UK commercial weight loss programme. Clin Obes 2014;4(6):324-32.

94. General Assembly of the World Medical Association. World Medical Association Declaration of Helsinki: ethical principles for medical research involving human subjects. J Am Coll Dent 2014;81(3):14.

95. Medical Research Council. MRC guidelines for management of global health trials - Involving Clinical or Public Health Interventions. MRC 2017. https://mrc.ukri.org/documents/pdf/guidelines-for-management-of-global-health-trials/ (accessed 4 Jan 2019).

For peer review only

For peer review only - http://bmjopen.bmj.com/site/about/guidelines.xhtml

Table 1 WHO Trial Registration Data Set

Data category	Information
Primary registry and trial identifying number	ISRCTN: 88405328
Date of registration in primary registry	16/12/2016
Secondary identifying numbers	H2020-EU.3.1 Grant agreement ID: 643309
Source(s) of monetary or material support	European Commission, Horizon 2020 - EU Framework Programme for Research and Innovation.
Primary sponsor	University of Leeds, Leeds, UK.
Secondary sponsor(s)	The Parker Institute, Copenhagen, Denmark.<br>University of Lisbon, Lisbon, Portugal.
Contact for public queries	Prof. James Stubbs (r.j.stubbs@leeds.ac.uk)<br>School of Psychology<br>Faculty of Medicine and Health<br>University of Leeds<br>Leeds<br>LS2 9JT<br>United Kingdom<br>+44 (0)113 343 3476
Contact for scientific queries	Prof. James Stubbs (r.j.stubbs@leeds.ac.uk)
Public title	NoHoW: Evidence-based ICT tools for weight loss maintenance.
Scientific title	A 2 x 2 randomised controlled trial to evaluate the effectiveness of evidence-based information and communications technology behaviour change tools for weight loss maintenance in overweight/obese adults after clinically significant weight loss.
Countries of recruitment	1. University of Leeds (UK),<br>2. The Parker Institute (Denmark),<br>3. University of Lisbon (Portugal).

For peer review only - http://bmjopen.bmj.com/site/about/guidelines.xhtml

Health condition(s) or problem(s) studied	Adults aged 18 or older who have intentionally lost at least 5% of their body weight in the last 12 months, and initially had a BMI of 25 or more.
Intervention(s)	Participants are assigned to one of four interventions:<br>1. Self-monitoring only (self-weighing and activity tracker),<br>2. Self-regulation and motivation,<br>3. Contextual-behavioural Science-based Emotion Regulation (CBS-emotion regulation),<br>4. Combined self-regulation, motivation and CBS-emotion regulation.
Key inclusion and exclusion criteria	Inclusion criteria:<br>1. Aged 18 years or older.<br>2. BMI (prior to weight loss) of ≥25 kg/m2.<br>3. Verification of ≥5% of weight loss in the last 12 months and remain 5% below their highest weight.<br>4. Ability to use a smartphone and have access to smart phone, tablet or computer with internet access and Wi-Fi.<br>5. Ability to use standing scales for weight measurements and must not be over 150kg (the scale limit 158kg)<br><br>Exclusion Criteria:<br>1. Inability to give informed consent.<br>2. Lost weight due to illness or surgical procedures, including bariatric procedures.<br>3. Pregnant or breastfeeding.<br>4. Involvement in another research intervention study that confound with the aims of NoHoW (excluding local health interventions and weight management services).<br>5. Inability to follow written material or telephone conversations in the English, Danish or Portuguese language (depending on the centre).<br>6. Diagnosed with an eating disorder (e.g. anorexia nervosa, bulimia nervosa, or purging disorder).<br>7. Diagnosed with any condition that may interfere with increasing mild to moderate physical activities and which is unstable (i.e. untreated or unable to be controlled by medication).

For peer review only

	8. Recent diagnosis with type 1 Diabetes.<br>9. Extensive planned travel, i.e. moving away or long-term travel abroad (e.g., more than four weeks)<br>10. Living in the same household as existing participant in the trial.
Study type	Interventional, 2x2 factorial randomised controlled trial.
Date of first enrolment	20 March 2017
Target sample size	1600
Recruitment status	Completed (31/03/2018), no longer recruiting.
Primary outcome(s)	Weight (kg)
Key secondary outcomes	1. Body composition: (e.g., fat mass and fat free mass) and waist/Hip ratio.<br>2. Biomarkers: blood pressure, heart rate, cortisol (hair samples), hbA1c, Cholesterol.<br>3. Behavioural: physical activity, sleep and dietary intake.<br>4. Psychological moderators and mediators of change for: self-regulation and motivation (e.g., action planning and coping, self-efficacy); CBS-emotion regulation (e.g., weight focused self-criticism, five dimensions of mindfulness); stress management (e.g., perceived stress scale, depression, anxiety and stress); and, Quality of life and wellbeing.<br>5. Process evaluation<br>6. Cost-effectiveness analysis

For peer review only - http://bmjopen.bmj.com/site/about/guidelines.xhtml

Table 2. Study recruitment and intervention schedule (SPIRIT 2013 guidelines).

	**Study Duration**				
	**Recruitment**	**Baseline**	**6 months**	**12 months**	**18 months**
**TIME POINT**	t_0	t_1	t_2	t_3	t_4
RECRUITMENT					
Screening	X				
Informed consent		X			
Randomisation and allocation		X			
INTERVENTION					
Fitbit device training		X			
Intake24 (food diary) training		X			
Toolkit training (after randomisation)		X			
Arms 2-4 active intervention		◆——	——◆		
Arms 2-4 follow-up			◆——	————	——◆
Arm 1 (control) intervention		◆——	————	————	——◆

For peer review only

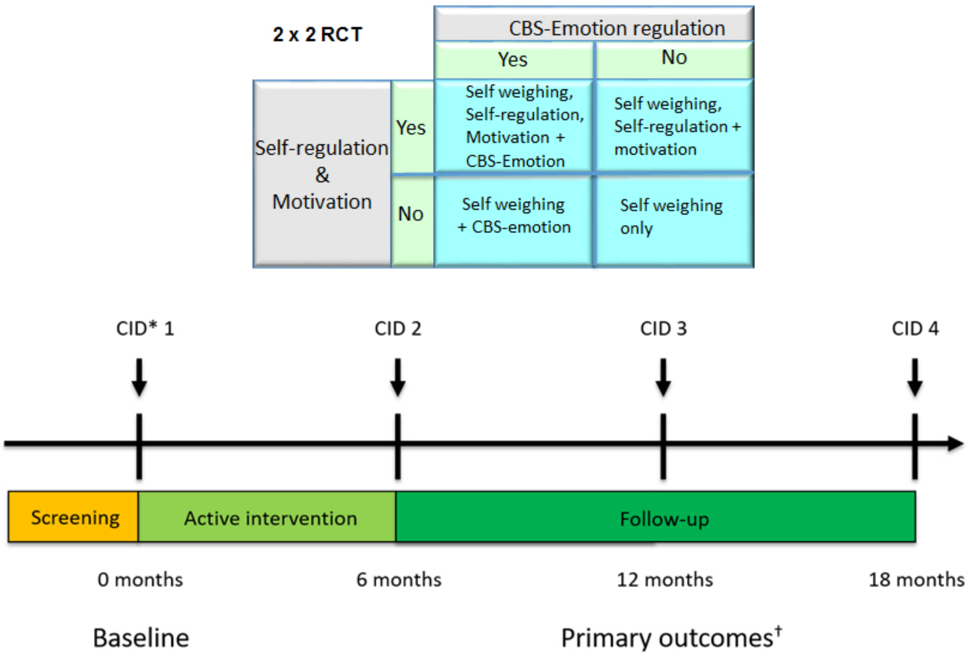

*CID, Clinical Investigation day.
†All measures are taken at CID 1-4 with the exception of the biomarkers which are taken at 0 and 12 months only. Process evaluation measures are taken at 1, 3, 6 and 12 months and focus groups at 6 months.

Figure 1. Trial design

220x178mm (96 x 96 DPI)

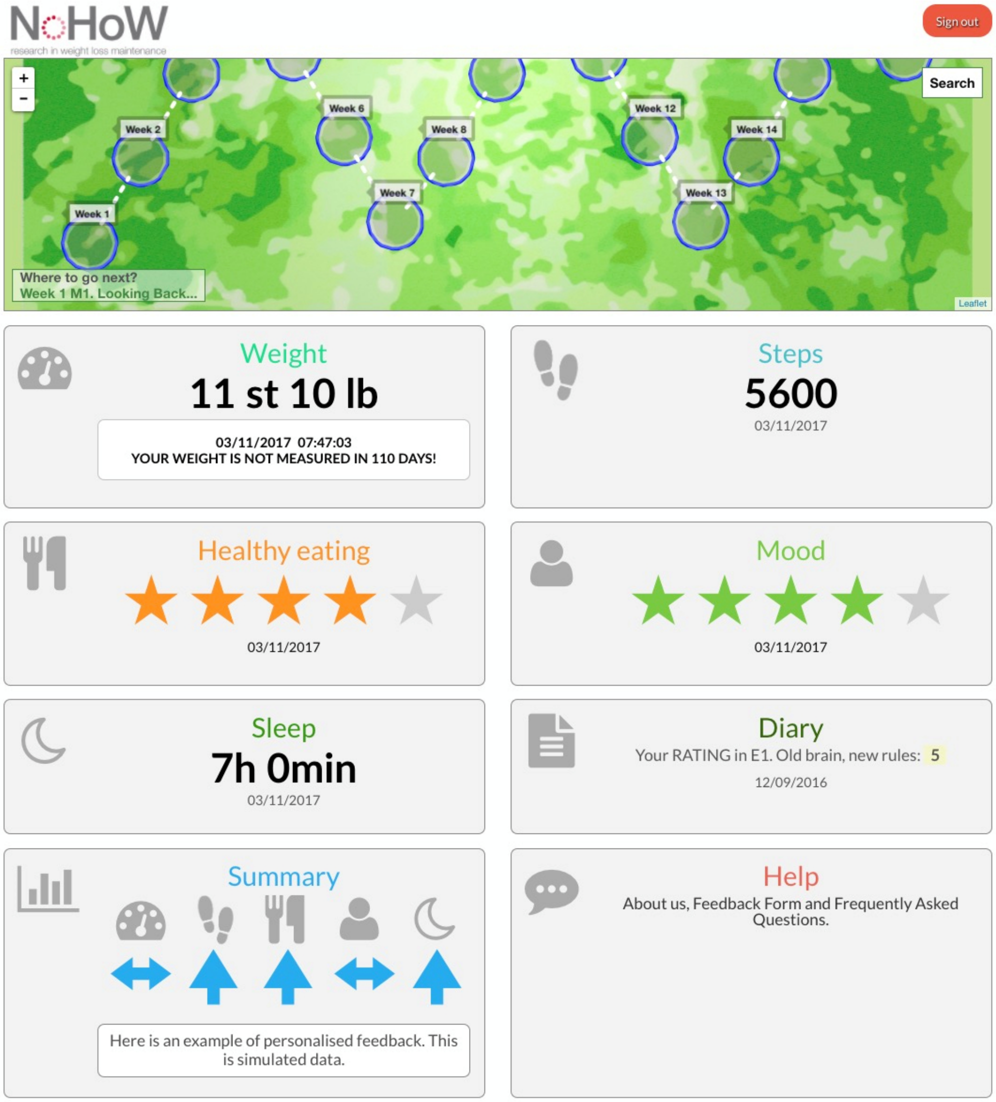

Figure 2. Toolkit example (arm 4).

375x418mm (72 x 72 DPI)

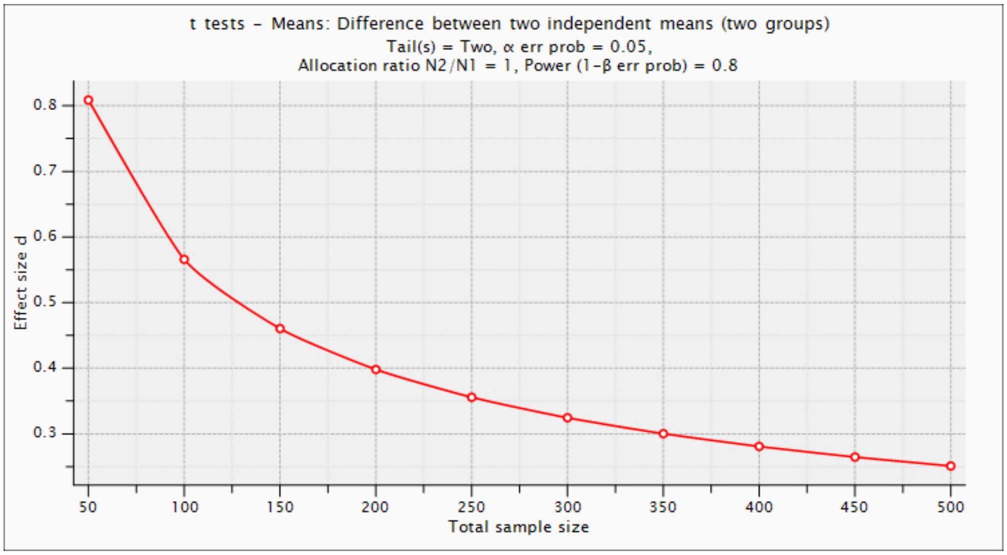

Figure 3. Plot of detectable effect size (Cohen's D).

197x108mm (96 x 96 DPI)

Supplementary file 1. Example consent form.

CONSENT FORM

NoHoW: Evidence-based ICT tools for weight loss maintenance

Please read the following statements and initial each box, as in order to continue in the study you must agree to the following statements

☐ I confirm that I have read and understood the participant information sheet (version 1, 27/02/2017), for the study named above and I have had the opportunity to ask questions and have had these answered satisfactorily.

☐ I consent for the research team to use the anonymised information obtained for research purposes (e.g., disseminating the results at conferences, in publications or for training purposes) and understand that the anonymised data will be kept for 20 years.

☐ I understand that information specific to this project may be looked at by responsible individuals from the research teams and will not be shared to anyone outside of the research teams.

☐ I understand that my participation is voluntary and that I am free to withdraw at any time, without giving any reason, without my medical care, or legal rights being affected.

☐ I agree to be contacted to be asked to fill in questionnaires and for measures to be taken initially at baseline, 6 months, 12 months and 18 months after the study has begun.

The following sections are optional- if you agree with a statement, please initial the box:

☐ I agree to take part in the study.

☐ I agree to provide a sample of a small section of hair, for the assessment of cortisol content, as a biomarker of longer term stress, initially at baseline and 12 months after.

For peer review only

Donation of blood for research is voluntary and you should not be placed under any pressures to do so. You do not have to agree to give a blood sample nor need to explain why you should choose not to donate. Any personal information provided by you in connection with the donation will be held in confidence. For reasons of safety, you should not donate if:

- You know, or think that you might be infected with Hepatitis B or Hepatitis C.
- You know, or think that you might be infected with HIV.
- You have a sexual partner who is infected with Hepatitis or HIV.
- You are unwell at the moment.
- You are anaemic or receiving treatment for anaemia or iron deficiency.

☐ I agree to provide a small blood sample by finger prick initially at baseline, and 12 months after.

☐ I wish to receive written feedback of my blood results which will include results of my full lipid profile (e.g., cholesterol), and HbA1c levels.

☐ Occasionally, results may fall out of the usual range which may be due to conditions such as high blood pressure, pre-diabetes, diabetes and high cholesterol. In these cases, we may wish to share the results of your measurements with yourself and your GP. I agree for the researchers to contact me and my GP

Name of participant: ………………………………………

Signature of participant: ……………………………………… Date: ……………

I confirm that the volunteer named above has been instructed of the nature and purpose of the study to be undertaken.

Signature of researcher:………………………………………………….. Date: ………………

The NoHoW Project has been approved by the School of Psychology Research Ethics Committee at the University of Leeds (17-0082; 27-Feb-2017). If you have any questions, please contact the principal Investigator, Professor James Stubbs, at r.j.stubbs@leeds.ac.uk.

For peer review only

Supplementary File 2. Study assessment schedule (SPIRIT 2013 guidelines).

		**Study Duration**				
		**Recruitment**	**Baseline**	**6 months**	**12 months**	**18 months**
**MEASURES**	**DESCRIPTION**	**t_0**	**t_1**	**t_2**	**t_3**	**t_4**
**DEMOGRAPHICS.**	Age, gender, occupation, education level, income, height, marital status and employment, smoking and alcohol history.	X	X			
**WEIGHT HISTORY.**	Self-report of current weight, weight 12 months ago, highest weight in last 12 months, highest weight, pregnancy, weight loss attempts, weight maintenance, relapse, weight loss methods, and parent's weight history.		X			
**MEDICAL HISTORY**	Name and dosage of medications, medical conditions, stability of conditions.	X	X	X	X	X
**DIGITAL HEALTH LITERACY.**	The eHEALS scale[84] assesses digital health literacy (i.e. knowledge of different health information on the internet and perceived confidence in one's ability to find or use such information to make health decisions). The scale consists of 8 items measured on a 5-point Likert scale ('1', Strongly disagree, to '5' Strongly agree).		X			
**ANTHROPOMETRICS**						
Weight.	Body weight (Seca 704s, SECA, Germany) in light clothing at 0,6, 12 and 18 months and at home throughout the duration of the trial via the Fitbit Aria smart scales ≥ twice weekly.		♦	─	─	♦

Height	Height (±0.1cm) is measured with participants barefoot, using a Seca 704s instrument (SECA, Germany).		X			
Body mass index	Body mass index (BMI) is calculated from weight (kg) and height (m) using the following equation: kg/m^2.	X	X	X	X	X
Waist hip ratio.	The waist measurement is taken against bare skin or light clothing at the top of the iliac crest and the hip measurement is taken at the maximum circumference over the buttocks using a tape measure. Average waist hip ratio measured by taking 2 fasted hip and waist circumference measures, or 3 if there is >1cm difference. The waist-hip ratio is calculated by dividing hip and waist circumference.		X	X	X	X
**BODY COMPOSITION**	Fasting bioimpedance (ImpediMed™ SFB7, Queensland, Australia) estimates total body water, extra- and intracellular fluid (l) and hence fat free mass (kg) and by subtraction fat mass (kg). It includes applying an electrical current at 256 frequencies ranging from 4 kHz to 1000 kHz, via a tetrapolar electrode configuration, following manufacturer's instructions. Assuming a constant hydration factor of 0.73 for fat free mass and that fat mass is hydrophobic, body water and weight can be used to estimate fat and fat free mass using a 2 compartment model.		X	X	X	X
**BIO-MARKERS**						
HbA1c.	Fasting capillary blood is analysed for HbA1c (mmol/mol, eAG and %) using the Afinion™ AS100 Analyzer (Alere, Stockport, UK). Sample cartridges are disposed of immediately after results are obtained from the machine.		X		X	
Full lipid profile.	Fasting capillary blood is analysed for full lipid profiles including total cholesterol (mmol/L), low density lipoprotein (LDL), high density lipoprotein (HDL), triglycerides, non-HDL and cholesterol/HDL (mmol/L) using the AfinionTM AS100 Analyzer (Alere). Sample cartridges are disposed of immediately after results are obtained from the machine.					
Hair samples.	Cortisol levels are measured by analysing 2cm of hair cut from close to the scalp. The sample is stored in foil inside a sealed plastic bag. User ID and hair information (e.g., the use of hair dye, hair product use and		X		X	

For peer review only

	hair wash frequency per week) is stored with the sample. Between 10 and 20 mg of hair from the 2 cm of the tuft closest to the scalp is accurately weighed, minced finely 1ml of methanol is added and the suspension incubated overnight at 50°C with a gentle shaking. The following day, the methanol is transferred into a clean tube and evaporated to dryness under nitrogen. The residue is reconstituted in 250-µl PBS buffer (pH 8.0). The cortisol concentration (pg/mg) in the resulting buffer solution is determined in duplicate using Enzyme-linked immunosorbent assay (ELISA).[48]					
Blood pressure.	An average of three fasting pulse, diastolic and systolic blood pressure measures are calculated and measured using the Microlife BP A2 (Gentle Technology, Microlife, Clearwater, FL, USA, Inc).		X	X	X	X
Heart rate.	Daily average resting heart rate is measured from minute-by-minute data collected throughout the duration of the trial via the Fitbit Charge 2.		◆	—	—	◆
**PHYSICAL ACTIVITY**						
Daily steps	Minute-by-minute steps are monitored by Fitbit Charge 2 for trial duration. The Charge 2 TM uses a wrist-worn tri-axial accelerometer, altimeter and vibration motor, which collects longitudinal movement data and estimates activity intensity, duration, number of steps, and distance travelled using proprietary algorithms (https://www.fitbit.com/charge2).		◆	—	—	◆
Intensity of physical activity (low, moderate and vigorous).	Time (minutes/day) spent in each intensity of activity is measured via self-report using the International Physical Activity Questionnaire (IPAQ) long-version[56] and is objectively measured using minute-by-minute activity monitored by Fitbit Charge 2 for trial duration.		X	X	X	X
Sitting behaviour.	Time (minutes/day) spent sitting is measured using the IPAQ.[56]		X	X	X	X
Activity choice.	The Activity Choice Index (ACI)[57] measures the ability to choose physical activity over less active or sedentary behaviours, such as using stairs or escalators. The scale consists of a 5-point Likert scale ('1', never, to '5' always).					

For peer review only

**SLEEP QUALITY AND QUANTITY**	Sleep quantity (hours/day) and quality are monitored by the Fitbit Charge 2 for the trial duration.		♦			♦
**DIETARY INTAKE AND EATING BEHAVIOUR**						
Dietary intake.	Four consecutive days of 24-hour dietary recalls, including one weekend day, using the web-based tool INTAKE24[58], which measures food intake and estimates daily energy (kcal) and nutrient intake from standardised food tables. Country specific food tables were inserted into the platform.		X	X	X	X
Cognitive restraint, disinhibition and hunger.	The Three Factor Eating Questionnaire (TFEQ)[59] is a 51-item, 3 factor measure that assesses cognitive restraint, disinhibition and hunger. The scale uses a true or false scale (items 1-36), a 4-point scale for items 37-49 and 51 ('1', rarely, to '4', always) and a 6-point scale for item 50 ('0', I eat whenever I want, to '5', constantly limiting food intake).		X	X	X	X
Controllability and automaticity.	Controllability and Automaticity of Eating Behaviour Questionnaire (CAEBQ) (unpublished scale) is a 24-item, 2 factor measure assessing eating control, grazing, binging and eating in the absence of hunger. Each subscale consists of 2-items assessed on a 5-point scale ('1', never, to '5', very often) and 4 items consisting of a 5-point Likert scale ('1' strongly disagree, to '5', strongly agree).		X	X	X	X
Eating in the absence of hunger.	Eating in The Absence of Hunger Scale (EAHS)[60] is a 28-item, single factor scale assessing the conditions when one is likely to eat in the absence of hunger. The 28 items assesses eating in response to emotional, physical, and environmental cues, using a 5-point scale ('1', never, to '5', always).		X	X	X	X
Reliance on hunger and satiety.	The reliance on hunger and satiety cues is a 6-item subscale of the Intuitive Eating Scale-2 (IES-2)[61] that measures the ability to guide eating behaviour based on one's internal hunger and satiety signals. The subscale uses a 5-point Likert scale ('1', strongly disagree, to '5', strongly agree).		X	X	X	X

Binge Eating.	The Binge Eating Scale (BES)[62] is a 16-item one-dimensional measure assessing the severity of binge eating symptoms. Each item consists of three to four statements from which respondents select one. Each option reflects a rating of severity ranging from 0 (no difficulties with binge eating) to 3 (severe binge eating symptoms).		X	X	X	X
Intuitive eating – 1.	Intuitive Eating Scale – 1 (IES-1)[53] is a 21-item trait measure to assess the ability to be aware and follow physical hunger and satiety cues to determine when, what and how much to eat. It includes three subscales: eating for physical rather than emotional reasons, unconditional permission to eat and reliance on hunger and satiety cues. IES-1 uses a 5-point Likert scale ('1' strongly disagree, to '5' strongly agree). The full scale is used at baseline only.		X			
Power of food.	Power of Food Scale[52] is a 10-item measure adapted from the original 15 item version which assesses the sensitivity to palatable foods within food-abundant environments. It includes three subscales: food availability, food present and food tasted. PFS uses a 5-point Likert scale ('1' do not agree at all, to '5', strongly agree).		X			
**WELL-BEING AND QUALITY OF LIFE**						
Quality of Life.	The EQ5D-5L[63] measures generic health related quality of life (HRQoL). It includes five dimensions (mobility, self-care, usual activities, pain/discomfort, and anxiety/depression) and has five levels of severity within each dimension: no problems (Level 1); slight; moderate; severe; and extreme problems (Level 5).		X	X	X	X
Wellbeing.	The 14-item single factor Warwick and Edinburgh Well-being Scale[64] measures mental wellbeing using 14 positively worded statements and uses a 5-point scale ('1', none of the time, to '5', all of the time).		X	X	X	X
**SELF-REGULATION, SELF-EFFICACY AND MOTIVATION**						

For peer review only

Action planning and coping planning	The Action Planning and Coping Planning Scales (APCP)[65] measures coping planning as a barrier-focused self-regulation strategy. The Action planning scale consists of 4 items and coping planning consists of 15 items both using a 5-point Likert scale ('1', Strongly disagree, to '5', Strongly agree').		X	X	X	X
Action control	The Action Control Scale[66] consists of 8 items and uses a 5 point Likert scale ('1', Strongly disagree, to '5', Strongly agree') that measures three distinct self-regulatory actions: self-monitoring, awareness of standards, and effort.		X	X	X	X
Basic psychological needs and frustration	The Basic Psychological Needs and Frustration Scale[67] has 16 items that uses a 7-point scale ('1', Strongly disagree to, '7', Strongly agree') to assess basic need satisfaction with 4 subscales: autonomy, competence, social support and relatedness. Items 2, 4, 6, 8, 12, and 16 were adapted to focus on weight management (e.g. 'I feel capable of managing my weight').		X	X	X	X
Goal content	The Goal Content for Weight Loss Maintenance Scale (GCWLMS)[68,69] was modified to focus on weight management rather than exercise behaviours (e.g. 'I manage my weight to…'). It consists of 16 items that uses a 7-point scale ('1', Strongly disagree, to '7', Strongly agree') to assess intrinsic and extrinsic goals.		X	X	X	X
Behavioural regulation in exercise	The Behavioural Regulation in Exercise Scale (BREQ-3)[71] includes 4 subscales that measure external, introjected, identified and intrinsic forms of regulation of exercise behaviour. It consists of 24 items and uses a 5-point scale ('0', Not true for me, to '4', Very true for me).		X	X	X	X
Regulation for eating behaviour	The Regulation for Eating Behaviour Scale (REBS)[71] is the shortened scale and consists of 18 items that assess autonomous and controlled motivation, and associated types of regulation (e.g. introjected regulation) for eating behaviour, using a 7-point scale ('1', Does not correspond at all, to '7', Corresponds exactly). One item from each sub-scale (items 3, 6, 10, 11, 18, 20) displayed unsatisfactory psychometric properties and were removed to create a shortened scale.		X	X	X	X
Self-efficacy	The Self-Efficacy for Weight Management Scale (SEWM)[72] has 12 items and uses a 100 point sliding scale ('0', not confident at all, to '100', extremely confident) to asses self-efficacy beliefs for exercise and eating.		X	X	X	X

For peer review only

Autonomy	The Index of Autonomous Functioning (IAF)[50] is a 15-item scale and uses a 5-point scale (‘1’, Not at all true, to ‘5’, Completely true) to assess trait autonomy across three theoretically derived subscales: authorship/self-congruence, interest-taking, and low susceptibility to control.		X			
Relapse Prevention	The Relapse Prevention Scale (RPS)[54] is a newly developed scale for trait-level weight management and consists of 14 items that measures three sub-domains: approach-based plans/goals, avoidance-based plans/goals, and cognitive factors. The scales uses 5-point scale (‘1’, Very unlikely, to ‘5’, Very likely).		X			
Life goals and aspirations	The Aspirations Index (AI)[51] is an 11-item scale that measures the degree to which people value seven broad goal contents: wealth, fame, image, personal growth, relationships, community contribution, and health. The scale uses a 9-point scale (‘1’, Not at all important, to ‘9’, Extremely important).		X			
Regulation for weight management.	The Regulation for Weight Management Scale (RWMS)[71] consists of 10 items and measures the reasons that underpin individual’s decisions to manage their weight (e.g. “Managing my weight is the part of the way I have chosen to live my life”). The scales uses a 7-point Likert scale (‘1’, Does not correspond at all, to ‘7’, Corresponds exactly) and was adapted from the Regulation of Eating Behaviour scale (REBS) to focus specifically on weight management.		X	X	X	X
**EMOTION REGULATION**						
Self-criticism and reassurance	The Weight Focused Self-Criticism/Self-Reassuring Scale (WFSCRS)[73] is a 22-item scale that assesses one’s thoughts and feelings about themselves during a perceived failure. Three subscales consist of self-criticising, (inadequate self and hated self) and tendencies to reassure the self (reassured self). The scale uses a 4-point scale (‘0’, Not at all like me, ‘4’, Extremely like me) and was adapted to focus on weight, body shape, and eating.		X	X	X	X
External shame	The Weight-Focused External Shame Scale (WFESS)[74] is a one-dimensional measure with 18 items that measure external shame (e.g., thinking that others negatively evaluate the self) and uses a 4-point scale (‘0’, Never, to ‘4’, Almost always).		X	X	X	X

For peer review only

Compassion	The Compassionate Attributes and Actions Scales (CAAS)[76] consist of three scales that measure self-compassion, compassion for others, and compassion from others. In this study, we omit the compassion for others scale as it is unrelated to the intervention logic models. Each scale consists of 6 items and uses a 10-point scale ('1', Never; to, '10', Always).		X	X	X	X
Body Image Acceptance	The Body Image Acceptance and Action Questionnaire (BIAAQ)[77] is a 12-item one-dimensional scale that measures body image-related psychological flexibility and the extent to which an individual accepts their beliefs and feelings about their body without attempting to change them while pursuing chosen values. The scale uses a 7-point scale ('1' Never true, to '7' Always true).		X	X	X	X
Engaged response (valued living and life-fulfilment)	The Engaged Living Scale (ELS)[78] consists of 16 items and measures two factors associated with psychological flexibility in line with Acceptance and Commitment Therapy: values living and life fulfilment. The scale uses a 5-point Likert Scale ('1', Completely disagree, to '5', Completely agree).		X	X	X	X
De-centering	The Experiences Questionnaire (EQ_DC)[79] has 11 items that assess three facets of the de-centering construct, including: (1) the ability to view the self as not synonymous with thoughts and feelings; (2) the ability to not habitually react to negative experiences ; and, (3) the capacity for self-compassion. The scale provides a total score of decentering and uses a 5-item scale ('1', Never, to '5', Always).		X	X	X	X
Mindful attention and awareness.	The Mindful Attention Awareness Scale (MAAS)[79] is a 15-item one-dimensional scale designed to assess dispositional mindfulness, that is, a receptive state of mind informed by a sensitive awareness of what is occurring in the present moment. The scales uses a 6-point scale ('1' Almost always, to '6' Almost never).		X	X	X	X
Emotion regulation.	The Difficulties in Emotion Regulation Scale (DERS)[80] is a one-dimensional scale that assesses the difficulty to regulate emotional responses by using adaptive emotion regulation strategies in the presence of negative emotions so that it does not lead to loss of control over behaviour or goal attainment. The scale consists of 16 items and uses a 5-point scale ('1', Almost always, to '5', Almost never) and is adapted for weight loss maintenance.		X	X	X	X

For peer review only

**STRESS MANAGEMENT**						
Perceived stress.	The Perceived Stress Scale (PSS)[81] measures the degree to which people perceive their lives as stressful (unpredictable, uncontrollable and overloaded) in the last month. The scale is one-dimensional and consists of 4 items and uses a 5-point scale (‘0’, Never, to ‘4’, Very often).		X	X	X	X
Depression and anxiety.	The Depression and Anxiety Scale (DASS)[82] short version consists of 21 items and measures three related negative emotional states individuals experienced over the last week, through 3 subscales with 7 items each: depression, anxiety and stress. The scales uses a 4 point scale (‘0’, Did not apply to me at all, to ‘3’, Applied to me very much).		X	X	X	X
**PROCESS EVALUATION AND USER EXPERIENCE**						
Initial toolkit impressions.	Initial impressions of the NoHoW Toolkit are captured at 1 month post-baseline and is adapted from the Technology Acceptance Model for Mobile Services (TAMM).[83] The survey consists of 14 items and uses a 5-point Likert scale (‘1’, Strongly disagree, to ‘5’, Strongly agree) to measure ease of adoption, ease of use, values and trust. Two additional items using a 11-point scale (‘0’, Not at all likely, to ‘10’, Extremely likely) assess how likely one would consider using the service in the future or recommend to a friend. Users are asked to rate the toolkit overall out of 10 and an open text box is provided for additional comments.		X			
User experience.	User experience is assessed at 3, 6 and 12 months using the TAMM[83] that assesses ease of adoption, ease of use, value, trust, and general usability using 25 items on a 5-point Likert scale (‘1’, Strongly disagree, to ‘5’, Strongly agree). Users are asked to rate the degree of importance, convenience, enjoyment, satisfaction, and motivation for 14 components of the Toolkit using a 5 point Likert scale (‘1’ Not at all, to ‘5’, Very). Two additional items using a 11-point scale (‘0’, Not at all likely, to ‘10’, Extremely likely) assess how likely			X	X	

For peer review only

	one would consider using the service in the future or recommend to a friend. Users are asked to rate the toolkit overall out of 10 and an open text box is provided for additional comments. Three items also assess toolkit usage: (1) 'On average, how frequently do you use the toolkit'; (2) 'What option describes your toolkit use behaviour best?'; and, (3) 'What motivated you to continue/why did you quit using the toolkit?'.					
Meta-data	The data-hub will collect meta-data for individual-level toolkit interactions including engagement and dose, such as the pattern of Toolkit and module use, intensity of use (e.g., timing, frequency of logins and duration of module/sub-module use), usage and content patterns (e.g., percentage of tasks performed and pages visited). Data are analysed using Matlab (MathWorks, Inc., Natick, Massachusetts, USA).		X	X	X	X

For peer review only

Running head: *NoHoW weight loss maintenance trial*

The NoHoW protocol: A multicenter 2x2 factorial randomized controlled trial investigating an evidence-based digital toolkit for weight loss maintenance in European adults.

Sarah E Scott[1], Cristiana Duarte[1], Jorge Encantado[2], Elizabeth H Evans[3], Marja Harjumaa[4] Berit L Heitmann[5-7], Graham Horgan[8], Sofus C Larsen[5], Marta M Marques[2, 11], Elina Matilla[4], Marcela Matos[9], Marie-Louise Mikkelsen[5], António L Palmeira[2], Beth Pearson[1], Lauren Ramsey[1], Kirby Sainsbury[3], Inês Santos[2], Falko F Sniehotta[3], Carol Stalker[10], Pedro J Teixeira[2], R James Stubbs[1*].

[1] School of Psychology, Faculty of Medicine and Health, University of Leeds, Leeds, United Kingdom.

[2] Faculdade de Motricidade Humana, Universidade de Lisboa, Lisboa, Portugal.

[3] Institute of Health & Society, Faculty of Medical Sciences, Newcastle University, Newcastle, United Kingdom.

[4]VTT Technical Research Centre of Finland, Oulu and Tampere, Finland.

[5]Research Unit for Dietary Studies, The Parker Institute, Bispebjerg and Frederiksberg Hospital, The Capital Region, Denmark.

[6]Department of Public Health, Section for General Practice, University of Copenhagen, Denmark

[7]Boden Institute of Obesity, Nutrition, Exercise & Eating Disorders, The University of Sydney, Sydney, Australia.

[8] Biomathematics & Statistics Scotland (James Hutton Institute), Aberdeen, United Kingdom.

[9] CINEICC, University of Coimbra, Coimbra, Portugal.

[10]College of Life and Natural Sciences, University of Derby, Derby, United Kingdom.

[11]Trinity Centre for Practice and Healthcare Innovation & ADAPT Centre, Trinity College Dublin, Dublin, Ireland

The work should be attributed to the School of Psychology, Faculty of Medicine and Health, University of Leeds, Leeds, United Kingdom.

Corresponding author: *Professor James Stubbs, School of Psychology, Faculty of Medicine and health, University of Leeds, Leeds, United Kingdom. E-mail: r.j.stubbs@leeds.ac.uk.

Word count: (excl. tables, abstract and references): 3999

Reporting checklist for protocol of a clinical trial.

Based on the SPIRIT guidelines.

Instructions to authors

Complete this checklist by entering the page numbers from your manuscript where readers will find each of the items listed below.

Your article may not currently address all the items on the checklist. Please modify your text to include the missing information. If you are certain that an item does not apply, please write "n/a" and provide a short explanation.

Upload your completed checklist as an extra file when you submit to a journal.

In your methods section, say that you used the SPIRIT reporting guidelines, and cite them as:

Chan A-W, Tetzlaff JM, Altman DG, Laupacis A, Gøtzsche PC, Krleža-Jerić K, Hróbjartsson A, Mann H, Dickersin K, Berlin J, Doré C, Parulekar W, Summerskill W, Groves T, Schulz K, Sox H, Rockhold FW, Rennie D, Moher D. SPIRIT 2013 Statement: Defining standard protocol items for clinical trials. Ann Intern Med. 2013;158(3):200-207

For peer review only

		Reporting Item	Page Number
Title	#1	Descriptive title identifying the study design, population, interventions, and, if applicable, trial acronym	1
Trial registration	#2a	Trial identifier and registry name. If not yet registered, name of intended registry	2, Table 1

Trial registration: data set	#2b	All items from the World Health Organization Trial Registration Data Set	Table 1 (page 7)
Protocol version	#3	Date and version identifier	7
Funding	#4	Sources and types of financial, material, and other support	Table 1, 21
Roles and responsibilities: contributorship	#5a	Names, affiliations, and roles of protocol contributors	21
Roles and responsibilities: sponsor contact information	#5b	Name and contact information for the trial sponsor	Table 1 (page 7)
Roles and responsibilities: sponsor and funder	#5c	Role of study sponsor and funders, if any, in study design; collection, management, analysis, and interpretation of data; writing of the report; and the decision to submit the report for publication, including whether they will have ultimate authority over any of these activities	N/A None
Roles and responsibilities: committees	#5d	Composition, roles, and responsibilities of the coordinating centre, steering committee, endpoint adjudication	7, 17, 21. Data monitoring committee is replaced with JHI, GH, data management

For peer review only

		committee, data management team, and other individuals or groups overseeing the trial, if applicable (see Item 21a for data monitoring committee)	and trial management meetings.
Background and rationale	#6a	Description of research question and justification for undertaking the trial, including summary of relevant studies (published and unpublished) examining benefits and harms for each intervention	3-6
Background and rationale: choice of comparators	#6b	Explanation for choice of comparators	3-6
Objectives	#7	Specific objectives or hypotheses	5-6
Trial design	#8	Description of trial design including type of trial (eg, parallel group, crossover, factorial, single group), allocation ratio, and framework (eg, superiority, equivalence, non-inferiority, exploratory)	6-7, 9 (for allocation)
Study setting	#9	Description of study settings (eg, community clinic, academic hospital) and list of countries where data will be	6-7

For peer review only

		collected. Reference to where list of study sites can be obtained	
Eligibility criteria	#10	Inclusion and exclusion criteria for participants. If applicable, eligibility criteria for study centres and individuals who will perform the interventions (eg, surgeons, psychotherapists)	7, Table 1. 9 (interventions conducted by trial staff)
Interventions: description	#11a	Interventions for each group with sufficient detail to allow replication, including how and when they will be administered	9-11
Interventions: modifications	#11b	Criteria for discontinuing or modifying allocated interventions for a given trial participant (eg, drug dose change in response to harms, participant request, or improving / worsening disease)	20
Interventions: adherance	#11c	Strategies to improve adherence to intervention protocols, and any procedures for monitoring adherence (eg, drug tablet return; laboratory tests)	11 (intervention adherence), 12 (participant retention to trial protocol)
Interventions: concomitant care	#11d	Relevant concomitant care and interventions that are permitted or prohibited during the trial	7, Table 1.

For peer review only

Outcomes	#12	Primary, secondary, and other outcomes, including the specific measurement variable (eg, systolic blood pressure), analysis metric (eg, change from baseline, final value, time to event), method of aggregation (eg, median, proportion), and time point for each outcome. Explanation of the clinical relevance of chosen efficacy and harm outcomes is strongly recommended	12-17, Supplementary file 2.
Participant timeline	#13	Time schedule of enrolment, interventions (including any run-ins and washouts), assessments, and visits for participants. A schematic diagram is highly recommended (see Figure)	Table 2, Supplementary file 2, Figure 1. Pp 7-11.
Sample size	#14	Estimated number of participants needed to achieve study objectives and how it was determined, including clinical and statistical assumptions supporting any sample size calculations	20
Recruitment	#15	Strategies for achieving adequate participant enrolment to reach target sample size	8

For peer review only

Allocation: sequence generation	#16a	Method of generating the allocation sequence (eg, computer-generated random numbers), and list of any factors for stratification. To reduce predictability of a random sequence, details of any planned restriction (eg, blocking) should be provided in a separate document that is unavailable to those who enrol participants or assign interventions	9
Allocation concealment mechanism	#16b	Mechanism of implementing the allocation sequence (eg, central telephone; sequentially numbered, opaque, sealed envelopes), describing any steps to conceal the sequence until interventions are assigned	9
Allocation: implementation	#16c	Who will generate the allocation sequence, who will enrol participants, and who will assign participants to interventions	9
Blinding (masking)	#17a	Who will be blinded after assignment to interventions (eg, trial participants, care providers, outcome assessors, data analysts), and how	9

For peer review only

Blinding (masking): emergency unblinding	#17b	If blinded, circumstances under which unblinding is permissible, and procedure for revealing a participant's allocated intervention during the trial	9
Data collection plan	#18a	Plans for assessment and collection of outcome, baseline, and other trial data, including any related processes to promote data quality (eg, duplicate measurements, training of assessors) and a description of study instruments (eg, questionnaires, laboratory tests) along with their reliability and validity, if known. Reference to where data collection forms can be found, if not in the protocol	12-17 and suppl. File 2.
Data collection plan: retention	#18b	Plans to promote participant retention and complete follow-up, including list of any outcome data to be collected for participants who discontinue or deviate from intervention protocols	12
Data management	#19	Plans for data entry, coding, security, and storage, including any related processes to promote data quality (eg, double data entry; range checks for data values). Reference to where	17-18

For peer review only

		details of data management procedures can be found, if not in the protocol	
Statistics: outcomes	#20a	Statistical methods for analysing primary and secondary outcomes. Reference to where other details of the statistical analysis plan can be found, if not in the protocol	18-20
Statistics: additional analyses	#20b	Methods for any additional analyses (eg, subgroup and adjusted analyses)	18-20
Statistics: analysis population and missing data	#20c	Definition of analysis population relating to protocol non-adherence (eg, as randomised analysis), and any statistical methods to handle missing data (eg, multiple imputation)	18-20
Data monitoring: formal committee	#21a	Composition of data monitoring committee (DMC); summary of its role and reporting structure; statement of whether it is independent from the sponsor and competing interests; and reference to where further details about its charter can be found, if not in the protocol. Alternatively, an explanation of why a DMC is not needed	17-18, there is no data monitoring committee, agreed by the Trial Steering Committee and is replaced by James Hutton Institute who developed the data-hub, led by GH.

For peer review only

Data monitoring: interim analysis	#21b	Description of any interim analyses and stopping guidelines, including who will have access to these interim results and make the final decision to terminate the trial	No interim analyses were used as stopping guidelines. The stop/go decision was determined by whether recruitment targets were met. These targets were achieved, as recorded on the trial registration.
Harms	#22	Plans for collecting, assessing, reporting, and managing solicited and spontaneously reported adverse events and other unintended effects of trial interventions or trial conduct	20-21
Auditing	#23	Frequency and procedures for auditing trial conduct, if any, and whether the process will be independent from investigators and the sponsor	N/A
Research ethics approval	#24	Plans for seeking research ethics committee / institutional review board (REC / IRB) approval	20-21
Protocol amendments	#25	Plans for communicating important protocol modifications (eg, changes to eligibility criteria, outcomes, analyses) to relevant parties (eg, investigators,	20-21

For peer review only

		REC / IRBs, trial participants, trial registries, journals, regulators)	
Consent or assent	#26a	Who will obtain informed consent or assent from potential trial participants or authorised surrogates, and how (see Item 32)	8
Consent or assent: ancillary studies	#26b	Additional consent provisions for collection and use of participant data and biological specimens in ancillary studies, if applicable	N/A
Confidentiality	#27	How personal information about potential and enrolled participants will be collected, shared, and maintained in order to protect confidentiality before, during, and after the trial	17-18
Declaration of interests	#28	Financial and other competing interests for principal investigators for the overall trial and each study site	21-22
Data access	#29	Statement of who will have access to the final trial dataset, and disclosure of contractual agreements that limit such access for investigators	22
Ancillary and post trial care	#30	Provisions, if any, for ancillary and post-trial care, and for compensation to	N/A we do not expect participants to be harmed, but

For peer review only

		those who suffer harm from trial participation	interventions will be discontinued from the trial if participants deem it detrimental to their well-being (p20).
Dissemination policy: trial results	#31a	Plans for investigators and sponsor to communicate trial results to participants, healthcare professionals, the public, and other relevant groups (eg, via publication, reporting in results databases, or other data sharing arrangements), including any publication restrictions	21
Dissemination policy: authorship	#31b	Authorship eligibility guidelines and any intended use of professional writers	21
Dissemination policy: reproducible research	#31c	Plans, if any, for granting public access to the full protocol, participant-level dataset, and statistical code	22
Informed consent materials	#32	Model consent form and other related documentation given to participants and authorised surrogates	Suppl file 1.
Biological specimens	#33	Plans for collection, laboratory evaluation, and storage of biological	Suppl file 2 (Blood samples are analysed by the Afinion

For peer review only

		specimens for genetic or molecular analysis in the current trial and for future use in ancillary studies, if applicable	Analyser and disposed of straight away).

The SPIRIT checklist is distributed under the terms of the Creative Commons Attribution License CC-BY-ND 3.0. This checklist can be completed online using https://www.goodreports.org/, a tool made by the EQUATOR Network in collaboration with Penelope.ai

For peer review only

BMJ Open

The NoHoW protocol: A multicenter 2x2 factorial randomized controlled trial investigating an evidence-based digital toolkit for weight loss maintenance in European adults.

Journal:	*BMJ Open*
Manuscript ID	bmjopen-2019-029425.R1
Article Type:	Protocol
Date Submitted by the Author:	05-Aug-2019
Complete List of Authors:	Scott, Sarah; University of Leeds, School of Psychology<br>Duarte, Cristiana; University of Leeds, School of Psychology<br>Encantado, Jorge; Universidade de Lisboa, Faculdade de Motricidade Humana<br>Evans, Elizabeth; Newcastle University, Institute of Health & Society<br>Harjumaa, Marja; VTT Technical Research Centre of Finland<br>Heitmann, Berit; Bispebjerg and Frederiksberg Hospital, The Parker Institut, Research Unit for Dietary Studies; University of Copenhagen, Department of Public Health<br>Horgan, Graham; Biomathematics and Statistics Scotland<br>Larsen, Sofus; Bispebjerg and Frederiksberg Hospital, The Parker Institute, Research Unit for Dietary Studies<br>Marques, Marta; Universidade de Lisboa, Faculdade de Motricidade Humana; University of Dublin Trinity College, Trinity Centre for Practice and Healthcare Innovation & ADAPT Centre<br>Mattila, Elina; VTT Technical Research Centre of Finland<br>Matos, Marcela; Universidade de Coimbra, CINEICC<br>Mikkelsen, Marie-Louise ; Bispebjerg and Frederiksberg Hospital, The Parker Institute, Research Unit for Dietary Studies<br>Palmeira, António; Universidade de Lisboa, Faculdade de Motricidade Humana<br>Pearson, Beth; University of Leeds, School of Psychology<br>Ramsey, Lauren; University of Leeds, School of Psychology<br>Sainsbury, Kirby; Newcastle University, Institute of Health and Society<br>Santos, Inês; Universidade de Lisboa, Faculdade de Motricidade Humana<br>Sniehotta, Falko; Newcastle University, Institute of Health & Society<br>Stalker, Carol; University of Derby, College of Life and Natural Sciences<br>Teixeira, PJ; Universidade de Lisboa, Faculdade de Motricidade Humana<br>Stubbs, James; University of Leeds, School of Psychology
**Primary Subject Heading**:	Public health
Secondary Subject Heading:	Patient-centred medicine
Keywords:	Weight loss maintenance, Digital health, Behaviour change, Self-regulation, Emotion regulation, Motivation

For peer review only - http://bmjopen.bmj.com/site/about/guidelines.xhtml

SCHOLARONE™
Manuscripts

NoHoW weight loss maintenance trial

The NoHoW protocol: A multicenter 2x2 factorial randomized controlled trial investigating an evidence-based digital toolkit for weight loss maintenance in European adults

Sarah E Scott[1], Cristiana Duarte[1], Jorge Encantado[2], Elizabeth H Evans[3], Marja Harjumaa[4] Berit L Heitmann[5], Graham Horgan[6], Sofus C Larsen[5], Marta M Marques[7], Elina Matilla[4], Marcela Matos[8], Marie-Louise Mikkelsen[5], António L Palmeira[2], Beth Pearson[1], Lauren Ramsey[1], Kirby Sainsbury[3], Inês Santos[2], Falko F Sniehotta[3], Carol Stalker[9], Pedro J Teixeira[2], R James Stubbs[1]*.

1 School of Psychology, Faculty of Medicine and Health, University of Leeds, Leeds, United Kingdom.

2 Faculdade de Motricidade Humana, Universidade de Lisboa, Lisboa, Portugal.

3 Institute of Health & Society, Faculty of Medical Sciences, Newcastle University, Newcastle, United Kingdom.

4 VTT Technical Research Centre of Finland, Oulu and Tampere, Finland.

5 Research Unit for Dietary Studies, The Parker Institute, Bispebjerg and Frederiksberg Hospital, The Capital Region, Denmark.

6 Biomathematics & Statistics Scotland (James Hutton Institute), Aberdeen, United Kingdom.

7 Trinity Centre for Practice and Healthcare Innovation & ADAPT Centre, Trinity College Dublin, Dublin, Ireland

8 CINEICC, University of Coimbra, Coimbra, Portugal.

9 College of Life and Natural Sciences, University of Derby, Derby, United Kingdom.

The work should be attributed to the School of Psychology, Faculty of Medicine and Health, University of Leeds, Leeds, United Kingdom.

Corresponding author: *Professor James Stubbs, School of Psychology, Faculty of Medicine and health, University of Leeds, Leeds, United Kingdom. E-mail: r.j.stubbs@leeds.ac.uk.

Word count: (excl. tables, abstract and references): 4685

For peer review only

ABSTRACT

Introduction: Obesity and associated diseases place a severe burden on healthcare systems. Behavioral interventions for weight loss (WL) are successful in the short-term but often result in weight regain over time. Self-regulation of eating and activity behaviors significantly enhance weight loss maintenance (WLM) and may be effectively augmented by contextual-behavioral approaches to emotion regulation. The NoHoW trial tests the efficacy of a theoretically informed, evidence-based digital toolkit using a mobile enabled website, activity trackers and Wi-Fi scales for WLM aiming to target: (i) self-regulation and motivation, and (ii) emotion regulation in adults who achieved clinically significant (≥5%) WL in the previous 12 months (initial BMI ≥25 kg/m^2).

Methods and analysis: The study is an 18 month, 3-center, 2 x 2 factorial single-blind, randomized controlled trial, which recruited 1627 participants achieving ≥5% WL between March 2017 to March 2018. Participants are randomly allocated to one of four arms: (i) self-monitoring only (self-weighing and activity tracker), (ii) self-regulation and motivation, (iii) emotion regulation, or (iv) combined self-regulation, motivation and emotion regulation. Participants attend four clinical investigation days at 0, 6, 12 and 18 months and are instructed to use the digital toolkit for 18 weeks during the first 6 months and at their discretion for the remaining 12 months. The primary outcome is change in weight (kg) at 12 months from baseline. Secondary outcomes are: body composition (e.g., bio-impedance analysis), health biomarkers (HbA1c, lipids, blood pressure, hair cortisol), dietary intake, physical activity, sleep, motivational, self-regulatory, emotion-regulatory moderators/mediators of WLM, engagement, user experience, acceptability and cost-effectiveness of the interventions.

For peer review only - http://bmjopen.bmj.com/site/about/guidelines.xhtml

Ethics and dissemination: Ethical approval was granted by institutional ethics committees at the Universities of Leeds (17-0082; 27-Feb-2017), Lisbon (17/2016; 20-Feb-2017) and Capital Region of Denmark (H-16030495, 8-Mar-2017). Results will be published in scientific journals.

Trial registration number: ISRCTN88405328

ARTICLE SUMMARY

Strengths and limitations of this study

- *Strengths*
- The first project to develop and evaluate (via RCT) an ICT toolkit combining continuous tracking of physical activity and body weight with theoretically informed, evidence-based digital interventions targeting self-regulation & motivation, and emotion regulation in a 2 x 2 design, to target long-term weight management.
- The 2x2 factorial design is a step forward from a conventional two-armed trial to determine the effect of self-regulation/motivation, emotion regulation interventions and their interactions on weight and health outcomes.
- Analyses of primary outcomes will test the hypotheses that self-regulation of energy balance behaviours improves longer-term weight outcomes and that emotion regulation strategies help prevent weight relapse. Moderator analysis will determine if participant psychological/behavioral profiles predict response to self-regulation or emotion-regulation

For peer review only

For peer review only - http://bmjopen.bmj.com/site/about/guidelines.xhtml

interventions. Mediation analyses will examine mechanisms of action by which self-regulation and emotion regulation-based approaches affect energy balance behaviours, weight and health.

•

• *Limitations*

• Participants are blinded to study allocation, but they can access similar interventions from other real-world sources.

• The active control arm includes self-monitoring of physical activity and weight but the inclusion of the Fitbit® devices across all arms potentially contaminates or diminishes the effect of self-regulatory components. Social support was excluded as a potential contaminant of the digital intervention.

INTRODUCTION

Overweight, obesity and associated diseases are key societal challenges. Obesity prevalence accounts for 3–8% of health costs and 10–13% of deaths in Europe.[1] This translates into €59 billion a year in direct, and an estimated €118 to €236 billion in indirect, health care costs.[2] These costs are set to rise in parallel with obesity prevalence.[3] Approximately 40% of the adult population in Europe report engaging in at least one weight control attempt in the previous 12 months.[4] Existing community-based programs support initial WL but are subject to high attrition or weight regain, limiting longer-term effectiveness.[5 6] The obesogenic environment and asymmetry of human energy balance (EB) regulation facilitates weight gain, while society criticizes people suffering from overweight and obesity, leading to stress and negative emotions, which can undermine WLM attempts.[7 8] Effective support for WLM or the prevention of weight regain needs to address sustained changes in EB behaviors (dietary,

For peer review only

physical activity and weight control) and additional emotional/psychosocial challenges that may undermine planned healthy behaviors.

Self-regulation and motivation

Core features of effective WLM interventions include behavior change techniques in line with self-regulation theories such as: 1) *self-monitoring* of weight and behavior; 2) *goal-setting*: agreement of clear weight targets/trigger points for weight control efforts; 3) feedback on behavior and weight; 4) *action plans* for weight control through dietary and physical activity behaviors and, 5) *plans* to cope with *risk factors* for weight regain and relapse prevention (e.g., problem solving).[9-15] Further, self-determined autonomous motivation has been consistently associated with change in EB behaviors for obesity management.[16 17] Interventions rooted in Self-Determination Theory may offer a promising approach to sustained EB and weight control.[12 17] A recent systematic review exploring psychological mediators of sustained effects in diet and lifestyle obesity interventions found that higher levels of autonomous motivation, self-efficacy, self-regulation skills, flexible eating restraint, and positive body image were mediators of medium to long-term weight control.[18] The combination of motivational and volitional processes of EB behaviors can contribute to effective WLM; that is, it appears those who are adequately motivated and who implement intentions tend to both initiate and maintain such changes.[19-21] However, physical activity and dietary interventions based on current behavior change theories characteristically achieve significant, but small and heterogeneous effects.[22] Additional psychological processes, such as emotion regulation, can potentially have incremental beneficial effects on the behavioral changes that promote WLM.[23]

For peer review only - http://bmjopen.bmj.com/site/about/guidelines.xhtml

Emotion regulation

Individuals with obesity or those trying to lose weight can experience increased stress, depression, anxiety and weight-related stigma[24 25] which may undermine weight control attempts. Repeated attempts at WL followed by weight regain can have a negative emotional impact, leading to self-critical thoughts and negative emotions. For some, 'comfort eating' may be a means of coping with these negative experiences, potentially derailing strategies of planned behavior.[26-28] Relapse prevention models include contextual behavioral (CB) based emotion regulation strategies such as compassion, acceptance and mindfulness.[29-31] Evidence suggests that mindfulness and acceptance of undesired internal experiences (e.g., negative emotions or self-criticism) and compassion for the self and others may help some individuals adopt adaptive behaviors that are congruent with their weight goals or values.[32-36] Mindfulness-based interventions show promise in changing obesity-related eating behaviors (e.g., binge eating and emotional eating), lowering depression levels and improving WLM.[37] Augmenting current self-regulatory approaches with CB emotion regulatory approaches could lead to more effective WLM.[32-36]

Digital behavior change interventions

Recently, there has been interest in digital interventions to promote and maintain health.[38] Digital solutions, such as smartphone applications and wearables, can be effective in supporting WLM if they are evidence-based, that apply a choice of behavior change techniques with individualized feedback,[22 39 40] encourage the use of self-regulatory techniques (e.g., self-monitoring) and develop intrinsic motivation.[41] Digital solutions are potentially cost-effective

For peer review only

For peer review only - http://bmjopen.bmj.com/site/about/guidelines.xhtml

and scalable to large populations, which could engage citizens in healthcare innovations that are convenient and effective for weight management in the face of limited public budgets.[42] To date, most commonly available weight management applications miss key evidence-based approaches for longer-term behavior change.[43 44]

Study objectives and hypotheses

The effectiveness of long-term evidence-based digital interventions and strategies to support WLM is limited.[45] The study's primary objectives are to evaluate whether using a new digital toolkit is effective for WLM by improving: (i) self-regulation and motivation, (ii) emotion regulation, or (iii) these factors in combination, compared to control (generic toolkit content, regular self-weighing and Fitbit® use) in 1627 participants at three European centers. We hypothesize that the combined intervention will be more effective for WLM compared to the self-regulatory or emotion regulatory interventions alone, which will also be more effective than the control group.

Secondary objectives are to determine how the intervention affects health markers (e.g., levels of glycated haemoglobin [HbA1c]) and body composition. The study will examine: (i) the intervention impact on physical activity, sleep, dietary intake, depression, anxiety, stress, quality of life and wellbeing; (ii) mediators of WLM, such as self-regulation (e.g., planning capacity), motivation (e.g., autonomous motivation) and emotion regulation processes (e.g., self-compassion) (iii) baseline moderators (e.g., gender, BMI) to identify participants who are more responsive to motivational and behavioral self-regulatory or emotion regulatory approaches for WLM; (iv) quantitative and qualitative assessment of user-experience,

For peer review only - http://bmjopen.bmj.com/site/about/guidelines.xhtml

acceptability, engagement and drop-out; (v) intervention cost-effectiveness; and (vi) to determine the efficacy of individualized feedback from data on physical activity self-tracking.

METHODS

Trial design and setting

The NoHoW trial is a 3-center (University of Leeds [UK], The Parker Institute [Denmark], and University of Lisbon [Portugal]) 2x2 factorial, randomized, single-blind, controlled trial testing the proof-of-concept of a digital toolkit for WLM. The study duration is 18-months with a follow-up at 6, 12 and 18-months post-baseline (Figure 1). The study is conducted between March 2017 and September 2019 in academic research institutes. In total, 1627 participants (~542 per center) were enrolled and randomized into one of four intervention arms: (1) active control arm (consisting of generic toolkit content, self-weighing and activity trackers, only); (2) self-regulation and motivation arm; (3) emotion regulation arm; and, (4) combined motivation/self-regulation and emotion regulation arm (Figure 1). The protocol is harmonized across trial centers using Good Clinical Practice (GCP) guidance, research-grade translation/back-translation of trial materials, two training workshops and weekly Trial Management meetings. The trial registration data set is displayed in Table 1. The final protocol (V2.1 20/09/2017) was approved by the Trial Steering Committee (TSC) and adheres to the SPIRIT guidelines.[46]

FIGURE 1 NEAR HERE Legend: **Figure 1.** Trial design

INSERT TABLE 1 HERE

For peer review only

For peer review only - http://bmjopen.bmj.com/site/about/guidelines.xhtml

Participants

Table 1 describes inclusion/exclusion criteria for the trial. Prior to randomization, potential participants are asked to provide documented verification (by a health professional, WL counsellor/friend, WL program record booklet, diary or smartphone app or before/after photographs[47]) that they have achieved a clinically significant WL of >5% during the previous 12 months.

Study recruitment

The study recruitment and intervention schedule is outlined in Table 2. Center-specific recruitment strategies were adopted for 12 months (March 2017- March 2018) and included: commercial WL program (UK, Slimming World); the Copenhagen Municipality weight management services, Dieticians from the Danish Association for Dieticians and commercial slimming companies (e.g., Sense, Henrik Duer and Per Nielsen); registered clinical dieticians/nutritionists who provide weight management services in Lisbon; leisure centers; and local/national media coverage and advertisements. All participants were directed to country-specific recruitment websites (http://uk.nohow.eu; http://dk.nohow.eu; http://nohow.fmh.ulisboa.pt) and completed an online eligibility screener using Qualtrics™. The eligibility screener categorized respondents as eligible, potentially eligible (wait-list) and ineligible. Eligible individuals were contacted for a telephone screening interview, provided with study information, medical history questions and the Physical Activity Readiness Questionnaire.[48] Eligible participants were invited to a Clinical Investigation Day (CID) where informed consent was obtained by research staff before randomization (Supplementary file 1).

For peer review only

For peer review only - http://bmjopen.bmj.com/site/about/guidelines.xhtml

Participants are re-consented before each subsequent visit and excluded if they become ineligible.

Public involvement

Members of the public that matched the study's inclusion criteria at the three trial centres were consulted via a survey to inform the recruitment strategies, trial conduct, questionnaires, assessment of burden and reviewing or testing the intervention content at all trial centers. During the toolkit development stage, the public were involved in two ways. First, a panel of 10 people, matching the trial criteria, tested the toolkit version 1 written in English for one month in the UK. A mixed methods approach was used for the analysis of the data assessing technical maturity and experiences, through questionnaires (quantitative data), interviews (qualitative data), TK usage log data, and data from wireless scales and activity trackers (Fitbit). These analyses will be published in a separate paper. The results were used to inform Toolkit version 2. Toolkit version 2 was then tested for 18 months using a panel of 30 people (10 in each centre) starting three months before the launch of the trial. Trial staff regularly collaborated with the panel to identify technological glitches that could be corrected early on so not to impact on the main trial participant.

***INSERT TABLE 2 HERE ***

Randomization and blinding

Participants were randomly allocated to treatment arms by researchers using an online trial administration portal. Adaptive stratified sampling using minimisation[49] was embedded in the portal using the R-program (R Foundation for Statistical Computing, Vienna, Austria) by the

For peer review only - http://bmjopen.bmj.com/site/about/guidelines.xhtml

study statistician GH. This minimizes differences in age, weight lost in the previous 12 months and current BMI between treatment arms and stratified by gender and center. Participants were blinded to study allocation. The research team were not blinded due to the need to train participants in arm-specific toolkit versions. The statistician (GH) is independent from data collection but not blinded during analyses due to inclusion of the arm allocation number. Independent researchers analyzing hair cortisol will be blinded to group allocation. No preliminary or ancillary analysis of outcome data between intervention arms will be conducted prior to data-lock (12 months).

Interventions

The toolkit is a mobile-enabled website, delivered as a portfolio of embedded tracking technologies, mini-apps, and web resources (Figure 2) accessible via computer, tablet and mobile phone. The tracking technologies consist of an activity and sleep tracker (Fitbit Charge 2™) and smart wireless body weight scale (Fitbit Aria™). Participants were asked to wear the Fitbit Charge 2 ™ at all times and to weigh themselves ≥ twice weekly by research staff. The logic models for each arm will be presented in a subsequent publication outlining the toolkit development and theoretical content. All arms include instructions for self-weighing and use of activity trackers. Arms 2 and 4 includes individualized weekly feedback about weight trajectories. Guidance for complex interventions was followed during the toolkit development specifying the intervention logic models and theory-driven behavior change techniques,[50] which will be detailed in a separate publication.

FIGURE 2 HERE Legend: **Figure 2**. Toolkit example (arm 4).

Arm 1 (Active control, self-monitoring, only)

For peer review only - http://bmjopen.bmj.com/site/about/guidelines.xhtml

The active control arm includes a toolkit dashboard containing weight, steps and sleep graphs using data from the Fitbit® devices, healthy eating and mood data (self-scored on a 5-point scale), a diary to populate with notes from these five tiles, and a help tile. The content is generic and does not relate to any theoretically informed, evidence-based behavior change approaches.

Arms 2-4.

After randomization, participants received standardized training from researchers during the first CID, specific to their arm allocation. Trial arms 2-4 include the same components as arm 1 but participants also receive a personal virtual road map (the 'NoHoW Journey') with intermediate destinations represented in that map by circles, each representing evidence-based theoretically-informed thematic modules targeting self-regulation and motivation (arm 2), emotion regulation (arm 3), and combined self-regulation and emotion regulation (arm 4). Arm 4 includes the same number of thematic modules as arms 2 and 3. Each weekly module opens into sub-modules. The active intervention involves 18 weekly sessions delivered throughout a six-month period. Users are encouraged to complete ≥1 sub-module per week following the sequence in the road map, consisting of videos/audios, interactive graphs, text, images, questionnaires and mini-apps. Weekly arm-specific emails are sent during the active six-month intervention phase, which provide WLM information, suggestions, and reminders to visit specific content. The toolkit automatically sends an email notifying participants to slow down if they complete three sessions ahead of schedule. After six months, they are encouraged to re-visit the toolkit for a further 12 months at their discretion.

Individualised feedback is a component of the self-regulation and motivation arms (arms 2 and 4) automatically provided by the toolkit in response to the individual's weight

For peer review only

For peer review only - http://bmjopen.bmj.com/site/about/guidelines.xhtml

266 trajectory. Feedback is generated from the data extracted from three sources: (1) weight from the Fitbit Aria™ scales; (2) activity and sleep from the Fitbit Charge 2™; and, (3) user engagement with the toolkit, including day of the week. After daily usage patterns are analysed, feedback is displayed in the toolkit consisting of one of a set of short statements (e.g., "your weight management appears better when you are more active").

Adherence

The toolkit sends automated weekly emails to participants to encourage engagement, prompt log-in, and recommends sessions to complete (arms 2-4 only). The control group receives the same number of emails but with links to generic content (e.g., topical diet/health information). Intervention adherence is continuously monitored by collecting information on the number of days since users last logged into the toolkit and the number of days since the last synchronization between the Fitbit® devices and data-hub. If no activity is detected after 21, 28 and 35 days, three attempts are made to contact participants by email. After 42 days of no activity, researchers telephone participants to resolve issues. Reasons for no activity are recorded.

Assessments

The primary outcome is change in weight (kg) between baseline and 12 months (primary end-point). Follow-up measures for WLM are completed at month 18 for comparability with other longitudinal studies.[22] All physical measurements are taken after a 10-12 hour overnight fast by trained research staff. Measures are taken at all four time points (baseline, 6, 12 and 18 months) except HbA1c, lipids and hair cortisol (baseline and 12 months only) and process

For peer review only - http://bmjopen.bmj.com/site/about/guidelines.xhtml

287 evaluation measures (1, 3, 6 and 12 months only). The study schedule and information for all
288 measures is in supplementary file 2.

289

290 Participant retention

291 Randomized participants are contacted at least two weeks before each visit and sent an
292 email/text reminder. If participants deviate from planned assessments, three attempts are made
293 to contact them by phone, email and a final written letter across one month. At least weight
294 (primary outcome) is obtained if possible.

295

296 Primary outcome

297 *Change in weight (kg) at 12 months from baseline*

298 Body weight (±0.1kg) is measured using a Seca 704s instrument (SECA, Germany) in
299 participants wearing light clothing. Participants are asked to weigh themselves at least twice
300 weekly, in the morning after voiding and before eating using the Fitbit Aria^{TM} scales for 18
301 months.

302

303 Secondary outcome measures

304 *Body composition and height*

For peer review only - http://bmjopen.bmj.com/site/about/guidelines.xhtml

305 Multi-frequency whole Bioimpedance spectroscopy (BIS) is measured by ImpediMed™ SFB7, Queensland, Australia), which measure impedance over a spectrum of frequencies for the estimation of body composition. Hanai mixture theory equations and standard resistivity constants[51] are used to calculate fluid volumes to estimate total body water. Height (±0.1cm) is measured with participants barefoot, using a Seca 704s instrument (SECA, Germany). See supplementary file 2.

311

312 *Waist Hip Ratio*

313 Waist and hip measurements are taken according to the World Health Organization's (WHO) guidance. A tape measure is used to record the hip and waist circumference to the nearest centimeter.[52] The waist-hip ratio is calculated by dividing hip and waist circumference.

316

317 *Biomarkers*

318 *HbA1c and Cholesterol*

319 Fasted capillary blood samples are collected to determine HbA1c (mmol/mol, %), estimated average glucose (eAG, mol/L) and full lipid profiles, including total cholesterol, low density lipoprotein (LDL), high density lipoprotein (HDL), triglycerides, non-HDL and cholesterol/HDL (mmol/L) assayed using a bench-top analyzer (Alere Afinion™ AS100 Analyzer, Alere, Stockport, UK).[53]

324

For peer review only - http://bmjopen.bmj.com/site/about/guidelines.xhtml

Blood Pressure heart rate

Systolic and diastolic blood pressure (BP) and resting heart rate (HR) (Microlife BP A2 Basic, Gentle Technology, Microlife, Clearwater, FL, USA, Inc.) are measured three times with the participant at rest in the sitting position. The mean value is recorded.

Hair Cortisol

Hair samples are collected by cutting a small tuft of hair close to the scalp to measure the stress hormone, cortisol and analyzed following a previously described protocol[54] (supplementary file 2).

Physical activity

Physical activity is measured by the self-reported International Physical Activity Questionnaire (IPAQ)[55] and Activity Choice Index (ACI).[56] Minute-by-minute physical activity data and heart rate are measured by the Fitbit Charge 2™ for the study duration.

Sleep quality and quantity

The Fitbit Charge 2™ estimates sleep quantity (hours/minutes) and quality (stages of sleep). Data are collected throughout the trial.

For peer review only

For peer review only - http://bmjopen.bmj.com/site/about/guidelines.xhtml

Dietary intake and eating behavior

Four consecutive 24-hour-web-based dietary recalls, including at least one weekend day, are collected within seven days of each visit using INTAKE 24.[57] Psychometric measures of eating behavior are assessed at each visit using the Three Factor Eating Questionnaire-51,[58] the Problematic Eating Patterns Scale (unpublished scale), Eating in the Absence of Hunger Scale,[59] Intuitive Eating Scale-2[60] and the Binge Eating Scale.[61]

Well-being and Quality of Life

Quality of life and wellbeing are assessed using the EQ5D-5L[62] and the Warwick-Edinburgh Well-Being Scale[63] respectively.

Self-regulation

Self-regulation is measured using the Action Planning and Coping Planning Scales,[64] and Action Control Scale.[65]

Motivation

Satisfaction of psychological needs are assessed using the Basic Psychological Needs and Frustrations Scale.[66] Quality of goal contents is assessed using the Goal Content for Weight Loss Maintenance Scale.[67 68] Autonomous and controlled motivation are assessed using the

For peer review only - http://bmjopen.bmj.com/site/about/guidelines.xhtml

362 Behavioral Regulation in Exercise Questionnaire (BREQ-3),[69] Regulation of Eating Behavior Scale (REBS),[70] and Regulations for Weight Management Scale (adapted from the REBS).[70]

364 *Self-efficacy*

365 Self-efficacy is assessed using the Self-Efficacy for Weight Maintenance Scale.[71]

366

367 *Emotion regulation and stress management*

368 CB-emotion regulation and stress management constructs are measured using the Weight Focused Self-Criticism/Self-Reassurance Scale,[72] Weight Focused External Shame Scale,[73 74] the Compassion Attributes and Actions Scales,[75] Body Image Acceptance and Action Questionnaire,[76] Engaged Living Scale,[77] Mindful Attention Awareness Scale,[78] adapted Difficulties in Emotion Regulation Scale,[79] and Experiences Scales (decentering sub-scale only).[80] Perceived stress is measured by the Perceived Stress Scale.[81] Anxiety and depression are measured using the Depression Anxiety Stress Scale.[82]

375

376 Demographics, weight history and participant traits.

377 A profiling questionnaire, adapted from the DiOGenes study,[83] is administered at baseline only to measure trait-specific moderators of WLM including demographics: age, gender, country, education, marital status, employment, and income. Also, smoking and alcohol use and number of times giving birth are recorded. Weight history includes: height, current and highest weight in the last 12 months, successful and unsuccessful WL attempts and strategies, and

For peer review only - http://bmjopen.bmj.com/site/about/guidelines.xhtml

psychological impact of living in a food abundant environment (the Power of Food Scale[84]). Self-determination traits are assessed using the Index of Autonomy Functioning[85] and Aspirations Index,[86] and self-regulation of appetite, hunger and satiety cues by the Intuitive Eating Scale-1.[87] Relapse prevention capability is measured using an adapted Relapse Prevention Scale.[88]

Process evaluation and user experience

Questionnaires adapted from the Technology Acceptance Model for Mobile Services (TAMM)[89] are administered by email to assess reasons for engagement/drop-out and user-experience (e.g., perceived ease-of-use and adoption, perceived value, trust, acceptability, and usability). Digital literacy is assessed at one month using the eHealth Literacy Scale (eHEALS).[90] Twelve focus groups are conducted (four in each country) at six months, by independent researchers, to thematically explore contextual factors (e.g., influence of wider social networks), user experience, and the toolkit use context in the three countries.[91] Meta-data on reach (participant characteristics), dose, fidelity and engagement with the toolkit is extracted from log files (Supplementary file 2). Methods and analyses with be detailed in separate publications.

Data management

A NoHoW data-hub was developed by the James Hutton Institute (Edinburgh) and led by GH, which collates, monitors and stores anonymized data securely from each center. Data from

For peer review only - http://bmjopen.bmj.com/site/about/guidelines.xhtml

each center is entered into trial management software (Easy Trial: www.easytrial.net). Quality checks are performed by all centers and reviewed during weekly Trial Management meetings. Separate publications will detail: (i) the technologies to host the toolkit, and (ii) the ICT architecture linking the toolkit, trial administration portal, trial management software and NoHoW data-hub.

Data Analysis

The 2 × 2 factorial design will be used in a model to investigate links between outcomes, and the extent of mediation, or moderation by covariates. For continuous outcomes, linear regression models will be used. The effects of different treatment arms will be presented with 95% confidence intervals of coefficients and p-values. For analyses that do not meet model assumptions, outcomes will be transformed prior to inferential comparisons. For categorical outcomes, logistic regression or categorical response models such as ordinal or multinomial regression will be used. Treatment effects will be presented as odd ratios, 95% confidence intervals, and p-values. Missing values will be imputed using multiple as part of an intention to treat (ITT) analysis.

To determine the mechanisms that underlie intervention efficacy, linear models are used to test whether the treatment (independent variable) causes a change in the potential mechanisms of change (mediators), which in turn, cause a change in the primary outcomes (dependent variables). Models also investigate the effects of covariates that may moderate the effect of the interventions. Although a repeated measures / mixed model might potentially detect treatment effects not apparent when 6 and 12 month outcomes are considered separately, our choice was

For peer review only

For peer review only - http://bmjopen.bmj.com/site/about/guidelines.xhtml

driven by the primary role of the 12 month outcome. To be considered worthwhile, a weight loss or maintenance effect of treatment must be detectable at 12 months, whatever might happen during the time interval between then and baseline. Because of this, we chose to define the primary outcome and its analysis at 12 months alone.

For the primary outcome of weight, we intend to present results from an analyses of completers, of compliant participants and of the ITT population. Baseline and last observation carried forward are likely to be optimistic for dropouts, implying full weight loss maintenance. So, for these dropouts, we will examine analyses which assume regain of all or a range of proportions of the weight lost since highest weight (in the 12 months before enrolment, for which all participants have provided evidence). Again, assuming return to baseline may be optimistic, but we do not have values at the time of greatest weight. Accordingly, we plan multiple imputation based on the imputed weight changes described above, using also centre and gender. This is not available for other health outcomes, such as HbA1c or blood pressure.

Mediation is assessed where change in weight, as represented by a categorical end-point (maintenance vs. weight regain) is the dependent variable, intervention group is the predictor variable, and self-regulatory, emotion regulatory and other psychosocial variables are mediators. Hybrid models, combining latent and observed variables, may be specified to ensure sufficient power to conduct the analysis and to stay within the limits of sample size requirements. Model fit will be assessed using the Chi-square goodness-of-fit test, Bollen's incremental fit index (IFI 0.95), and the standardized root mean residual (SRMR G 0.08) values. Tests of mediation will be conducted using a resampling method. To test the

For peer review only

For peer review only - http://bmjopen.bmj.com/site/about/guidelines.xhtml

significance of specific indirect effects of multiple mediators (total and specific for each mediator, using both normal theory and bootstrap procedures), a tool developed by Selig and Preacher[92] will be used.

Subgroup Analysis

Interactions between condition factors and baseline participant groupings (center, gender, age, BMI at entry, % WL at entry) will be prioritized as *a priori* sub group analyses in the primary and secondary outcomes. Age, BMI and weight will be analyzed as categorical variables, to avoid assumptions of linearity (or the complexity of fitting polynomial terms) and for ease of presentation. These pre-planned analyses will be conducted by the inclusion of appropriate interaction terms in the regression models. Where interactions are significant, results for each subgroup will be reported. This will mitigate the problems of multiple testing due to many subgroup comparisons. We recognize that statistical power in subgroups will vary with sample size and provide a plot of detectable effect size versus sample size for reference (Figure 3).

FIGURE 3 NEAR HERE Legend: **Figure 3**. Plot of detectable effect size (Cohen's D)

Cost-effectiveness analyses

Cost-effectiveness analysis will be conducted to model costs and intervention effects for a lifetime horizon. An existing decision-analytic model will be used to estimate BMI category transitions, incidence of secondary diseases (e.g., Type II diabetes), quality of life and healthcare costs.[93] Quality-adjusted life-years (QALYs) and costs will allow calculation of Incremental Cost-effectiveness Ratios (ICERs) and estimates of cost-utility. Extensive

For peer review only - http://bmjopen.bmj.com/site/about/guidelines.xhtml

sensitivity analyses will test the modelling assumptions. Exploration of scenarios relating *inter alia* to the cost and effects (including sustainability) of the intervention. Secondary cost-effectiveness analysis will explore WLM outcomes (e.g., cost per Kg maintained per year). The model will be adapted separately for each country.

Power calculations and sample size estimation

Power calculations were based on the primary outcome (weight change). To detect a difference between treatment arms of >1.5kg body weight gives a (Cohen's) d value of 0.25.[22] For 80% power, comparing >two groups requires a sample size of 250 per trial arm. To detect an effect size of 0.25 SD units for HbA1c, 245 participants in each trial arm gives 80% power, at 5% significance. Assuming 38% drop out,[83] a sample size of 1600 (533 per center) is needed to achieve a sample of 1002 (334 per center, ~250 per trial arm) participants at 12 months.

Ethical considerations and dissemination plans

The trial was registered with the ISRCTN registry (ISRCTN88405328). Ethical approval was granted by each institutional ethics committee before study commencement at each center. The protocol complies with relevant EC legislation, international conventions and declarations relating to ethical research practices.[94] Interventions will be discontinued if they are reported detrimental. Protocol deviations, violations, and serious adverse events or adverse events are recorded by trial staff and monitored by the Principal Investigator (JS) and TSC.[95] Any protocol amendments that impact the study conduct are approved by each institutional ethics committee

For peer review only - http://bmjopen.bmj.com/site/about/guidelines.xhtml

and communicated to participants. Trial results will be published in peer-reviewed scientific journals. Baseline data will be analyzed after the end of recruitment (March 2018) and outcome analyses after one-year data is collected (March 2019). Authorship will be determined by the guidelines set out by the International Committee of Medical Journal Editors.

Potential outcomes and impact

This is the first project to develop and formally evaluate (via RCT) a new ICT toolkit combining continuous tracking of physical activity and body weight with theoretically informed, evidence-based digital interventions targeting self-regulation & motivation, and emotion regulation in a 2 x 2 design, to target long-term weight management.

The NoHoW toolkit comprises evidence-based behavior change techniques and emotion regulation strategies, which are hypothesized mechanisms of action that may impact energy balance behaviours and longer-term weight outcomes. Analyses of primary outcomes will enable us to test the hypotheses that self-regulation of energy balance behaviours improves longer-term weight outcomes and that emotion regulation strategies help prevent weight relapse. Secondary outcomes of the trial moderator effects will enable us to determine if different psychological and behavioral profiles of participants predict response to self-regulation or emotion-regulation interventions. Mediation analyses will enable us to determine the mechanisms of action by which self-regulation and emotion regulation-based behavior change approaches affect energy balance behaviours and longer-term weight and health. Secondary analyses will inform more personalized digital interventions that better match intervention content and delivery to individual user needs, potentially helping to improve user engagement, health and wellbeing of participants.

For peer review only

For peer review only - http://bmjopen.bmj.com/site/about/guidelines.xhtml

Limitations

Participants are blinded to study allocation, but they can access similar interventions from other real-world sources. The 2 x 2 design involved an active control. A 2 x 2 plus 1 design including a no-intervention control would have enabled evaluation of self-tracking using Fitbit® devices, versus no intervention.

The active control arm includes self-monitoring of physical activity and weight but the inclusion of the Fitbit® devices across all arms potentially contaminates or diminishes the effect of self-regulatory components.

There is a potential conflict inherent in the design of trial that seeks to allow a certain degree of personalisation of intervention content use and maintain the integrity of trial arms. For this reason the degree of personalization was constrained to choice in the use of available self-regulatory or contextual emotion regulatory content within respective trial arms.

Social support was deliberately not part of this intervention but it is known that degree of social support can be important for weight management.

Acknowledgements

The authors thank David Meads, Marlene Silva, Cláudia Ferreira, Paul Sacher, Heather Patrick, Ian Caterson, John Blundell, all members of the NoHoW Consortium and the independent members of the NoHoW trial steering committee (Sharon Simpson, Paul Aveyard, Lucy Yardley and Annette Kjær Ersbøll).

For peer review only

For peer review only - http://bmjopen.bmj.com/site/about/guidelines.xhtml

Author Contributions: RJS, BLH, PT, FFS, ALP and GH conceived the study. RJS is the principle investigator; BLH is the grant coordinator; and SES and CD are the trial managers. SCL, ALP and SES are site coordinators. GH is the trial statistician and leads data management. MH and EM are responsible for the technological development and management of the NoHoW digital toolkit. ALP, MMM, MM and CD developed the content of the arms of the digital toolkit. JE, IS, MLM, CS, BP and LR conducted the trial activities at each respective trial sites. EHE and KS were involved in the development of some of the trial outcome measures. SES, CD and RJS drafted the manuscript and all authors revised and approved the final version. All authors made substantial contributions to the conceptualization of the study design and conduct of the protocol.

Disclosure of funding: This project has received funding from the European Union's Horizon 2020 research and innovation programme under grant agreement No 643309. The material presented and views expressed here are the responsibility of the author(s) only. The EU Commission takes no responsibility for any use made of the information set out. MMM is funded by the Marie-Sklodowska-Curie (EDGE) Fellowship programme [grant agreement No. 713567]

Competing interests: RJS consults for Slimming World through the University of Leeds. MMM and GH also conducted consultancy work with Slimming World. Slimming World were a partner of the project to aid recruitment within the UK but will not be involved in the analysis and interpretation of the trial.

Provenance and peer review: Not commissioned; externally peer-reviewed.

For peer review only

For peer review only - http://bmjopen.bmj.com/site/about/guidelines.xhtml

Data sharing statement: Subject to intellectual property (IP) negotiations, data will be made available in an open access data repository.

REFERENCES

1. Hunt A, Ferguson J. Health costs in the European Union: how much is related to EDCS. Brussels, The Health and Environmental Alliance, 2014. https://www.env-health.org/IMG/pdf/18062014_final_health_costs_in_the_european_union_how_much_is_realted_to_edcs.pdf (accessed 04 Jan 2019).

2. World Health Organization. The challenge of obesity in the WHO European region and the strategies for response. Copenhagen, Denmark Europe, 2007. http://www.euro.who.int/__data/assets/pdf_file/0008/98243/E89858.pdf (accessed 04 Jan 2019)

3. Wang YC, McPherson K, Marsh T, et al. Health and economic burden of the projected obesity trends in the USA and the UK. Lancet 2011;378(9793):815-25.

4. Santos I, Sniehotta F, Marques M, et al. Prevalence of personal weight control attempts in adults: a systematic review and meta-analysis. Obes Rev 2017;18(1):32-50.

5. Franz MJ, VanWormer JJ, Crain AL, et al. Weight-loss outcomes: a systematic review and meta-analysis of weight-loss clinical trials with a minimum 1-year follow-up. J Am Diet Assoc 2007;107(10):1755-67.

For peer review only - http://bmjopen.bmj.com/site/about/guidelines.xhtml

6. Brownell KD, Marlatt GA, Lichtenstein E, et al. Understanding and preventing relapse. Am Psychol 1986;41:765-82.

7. Stubbs RJ, Gail C, Whybrow S, et al. The evolutionary inevitability of obesity in modern society: implications for behavioral solutions to weight control in the general population. In: Martinez MP, Robinson H, eds. Obesity and Weight Management: Challenges, Practices and Health Implications. New York: Novo Publishers 2012.

8. Sainsbury K, Evans EH, Pedersen S, et al. Attribution of weight regain to emotional reasons amongst European adults with overweight and obesity who regained weight following a weight loss attempt. Eat Weight Disord 2018;1-11 doi: 10.1007/s40519-018-0487-0 [published Online First: 16 February 2018]

9. Stubbs RJ, Lavin JH. The challenges of implementing behaviour changes that lead to sustained weight management. Nutr Bull 2013;38:5-22.

10. Dombrowski SU, Avenell A, Sniehott FF. Behavioural interventions for obese adults with additional risk factors for morbidity: systematic review of effects on behaviour, weight and disease risk factors. Obes Facts 2010;3(6):377-96.

11. Michie S, Abraham C, Whittington C, et al. Effective techniques in healthy eating and physical activity interventions: a meta-regression. Health Psychol 2009;28(6):690-701.

12. Teixeira PJ, Going SB, Sardinha LB, et al. A review of psychosocial pre-treatmant predictors of weight control. Obes Rev 2005;6:43-65.

For peer review only

For peer review only - http://bmjopen.bmj.com/site/about/guidelines.xhtml

13. Silva MN, Markland D, Carraca EV, et al. Exercise autonomous motivation predicts 3-yr weight loss in women. Med Sci Sports Exerc 2011;43(4):728-37.

14. Kwasnicka D., Presseau J., White M., et al. Does planning how to cope with anticipated barriers facilitate health-related behavior change? A systematic review. Health Psychol Rev 2013;7:129-45.

15. Dombrowski SU, Sniehotta FF, Avenell A, et al. Identifying active ingredients in complex behavioural interventions for obese adults with obesity-related co-morbidities or additional risk factors for co-morbidities: a systematic review. Health Psychol Rev 2012;6(1):7-32.

16. Silva MN, Markland D, Carraça EV, et al. Using self-determination theory to promote physical activity and weight control: A randomized controlled trial in women. J Behav Med 2011;33:110-22.

17. Teixeira PJ, Mata J, Williams GC, et al. Self-regulation, motivation, and psychosocial factors in weight management. J Obes 2012;2012: 582348.

18. Teixeira PJ, Carraça EV, Marques MM, et al. Successful behavior change in obesity interventions in adults: a systematic review of self-regulation mediators. BMC Med 2015;13(1):84.

19. Hagger MS, Chatzisarantis NL. An integrated behavior change model for physical activity. Exerc Sport Sci Rev 2014;42(2):62-69.

20. Teixeira PJ, Silva MN, Mata J, et al. Motivation, self-determination, and long-term weight control. Int J Behav Nutr Phys Act 2012;9:22.

For peer review only

For peer review only - http://bmjopen.bmj.com/site/about/guidelines.xhtml

610 21. Verstuyf J, Patrick H, Vansteenkiste M, et al. Motivational dynamics of eating regulation: a self-determination theory perspective. Int J Behav Nutr Phys Act 2012;9(1):21.

612 22. Dombrowski SU, Knittle K, Avenell A, et al. Long term maintenance of weight loss with non-surgical interventions in obese adults: systematic review and meta-analyses of randomised controlled trials. BMJ 2014;348:g2646.

615 23. O'Reilly GA, Cook L, Spruijt-Metz D, et al. Mindfulness-based interventions for obesity-related eating behaviours: a literature review. Obes Rev 2014;15(6):453-61.

617 24. Dallman MF, Pecoraro N, Akana SF, et al. Chronic stress and obesity: a new view of "comfort food". Proc Natl Acad Sci U S A 2003;100(20):11696-701.

619 25. Block JP, He Y, Zaslavsky AM, et al. Psychosocial stress and change in weight among US adults. Am J Epidemiol 2009;170(2):181-92.

621 26. Gibson EL. The psychobiology of comfort eating: implications for neuropharmacological interventions. Behav Pharmacol 2012;23(5-6):442-60.

623 27. Lebel JL, Lu J, Dube L. Weakened biological signals: highly-developed eating schemas amongst women are associated with maladaptive patterns of comfort food consumption. Physiol Behav 2008;94(3):384-92.

626 28. Pedersen S, Sniehotta FF, Sainsbury K, et al. The complexity of self-regulating food intake in weight loss maintenance. A qualitative study among short-and long-term weight loss maintainers. Soc Sci Med 2018;208:18-24.

For peer review only - http://bmjopen.bmj.com/site/about/guidelines.xhtml

629 29. Hendershot CS, Witkiewitz K, George WH, et al. Relapse prevention for addictive behaviors. Subst Abuse Treat Prev Policy 2011;6:17.

631 30. Bowen S, Chawla N, Witkiewitz K. Mindfulness-based relapse prevention for addictive behaviors. In: Baer RA, ed. Mindfulness-Based Treatment Approaches: A Clinician's Guide. 2nd ed. San Diego, CA: Elsevier Academic Press. 2014:141-57.

634 31. Duarte C, Stubbs J, Pinto-Gouveia J, et al. The impact of self-criticism and self-reassurance on weight-related affect and well-being in participants of a commercial weight management programme. Obes Facts 2017;10(2):65-75.

637 32. Lillis J, Hayes SC, Bunting K, et al. Teaching acceptance and mindfulness to improve the lives of the obese: A preliminary test of a theoretical model. Ann Behav 2009;37:58-69.

639 33. Forman EM, Butryn ML, Hoffman KL, et al. An open trial of an acceptance-based behavioral intervention for weight loss. Cogn Behav Pract 2008;16:223-35.

641 34. Forman EM, Butryn ML, Juarascio AS, et al. The mind your health project: a randomized controlled trial of an innovative behavioral treatment for obesity. Obesity 2013;21(6):1119-26.

643 35. Niemeier HM, Leahey T, Palm Reed K, et al. An acceptance-based behavioral intervention for weight loss: a pilot study. Behav Ther 2012;43(2):427-35.

645 36. Tapper K, Shaw C, Ilsley J, et al. Exploratory randomised controlled trial of a mindfulness-based weight loss intervention for women. Appetite 2009;52(2):396-404.

647 37. Ohsiek S, Williams M. Psychological factors influencing weight loss maintenance: an integrative literature review. J Am Assoc Nurse Pract 2011;23(11):592-601.

For peer review only - http://bmjopen.bmj.com/site/about/guidelines.xhtml

649 38. Yardley L, Choudhury T, Patrick K, et al. Current issues and future directions for research into digital behavior change interventions. Am Journal Prev Med 2016;51(5):814-15.

651 39. MacLean PS, Wing RR, Davidson T, et al. NIH working group report: innovative research to improve maintenance of weight loss. Obesity 2015;23(1):7-15.

653 40. Evans EH, Araújo-Soares V, Adamson A, et al. The NULevel trial of a scalable, technology-assisted weight loss maintenance intervention for obese adults after clinically significant weight loss: study protocol for a randomised controlled trial. Trials 2015;16(1):421.

656 41. Lentferink AJ, Oldenhuis HK, de Groot M, et al. Key Components in eHealth Interventions Combining Self-Tracking and Persuasive eCoaching to Promote a Healthier Lifestyle: A Scoping Review. J Med Internet Res 2017;19(8).

659 42. European Commission. eHealth Action Plan 2012-2020. 2012. http://ec.europa.eu/digital-agenda/en/news/ehealth-action-plan-2012-2020-innovative-healthcare-21st-century. (accessed 4 Jan 2019).

662 43. Pagoto S, Schneider K, Jojic M, et al. Evidence-based strategies in weight-loss mobile apps. Am Journal Prev Med 2013;45(5):576-82.

664 44. Breton ER, Fuemmeler BF, Abroms LC. Weight loss—there is an app for that! But does it adhere to evidence-informed practices? Transl Behav Med 2011;1(4):523-29.

666 45. Hutchesson M, Rollo M, Krukowski R, et al. eHealth interventions for the prevention and treatment of overweight and obesity in adults: a systematic review with meta-analysis. Obes Rev 2015;16(5):376-92.

For peer review only - http://bmjopen.bmj.com/site/about/guidelines.xhtml

669 46. Chan A-W, Tetzlaff JM, Altman DG, et al. SPIRIT 2013 statement: defining standard
670 protocol items for clinical trials. Ann Intern Med 2013;158(3):200-07.

671 47. Klem ML, Wing RR, McGuire MT, et al. A descriptive study of individuals successful at
672 long-term maintenance of substantial weight loss. Am J Clin Nutr 1997;66(2):239-46.

673 48. Thomas S, Reading J, Shephard RJ. Revision of the physical activity readiness
674 questionnaire (PAR-Q). Can J Sport Sci 1992;17(4):338-45.

675 49. Altman DG, Bland JM. Treatment allocation by minimisation. BMJ 2005;330(7495):843.

676 50. Craig P, Dieppe P, Macintyre S, et al. Developing and evaluating complex interventions:
677 the new Medical Research Council guidance. Int J Nurs Stud 2013;50(5):587-92.

678 51. De Lorenzo A, Andreoli A, Matthie J, et al. Predicting body cell mass with bioimpedance
679 by using theoretical methods: a technological review. J Appl Physiol 1997;82(5):1542-58.

680 52. World Health Organisation. Guide to physical measurements (Step 2) 2008.
681 https://www.who.int/ncds/surveillance/steps/Section%204%20Step%202%20Physical%20M
682 easurements.pdf (accessed 4 Jan 2019).

683 53. Jain A, Rao N, Sharifi M, et al. Evaluation of the point of care Afinion AS100 analyser in
684 a community setting. Ann Clin Biochem 2017;54(3):331-41.

685 54. Van Uum S, Sauve B, Fraser L, et al. Elevated content of cortisol in hair of patients with
686 severe chronic pain: a novel biomarker for stress. Stress 2008;11(6):483-88.

For peer review only

For peer review only - http://bmjopen.bmj.com/site/about/guidelines.xhtml

55. Craig CL, Marshall AL, Sjostrom M, et al. International physical activity questionnaire: 12-country reliability and validity. Med Sci Sports Exerc 2003;35(8):1381-95.

56. Mullen SP, Silva MN, Sardinha LB, et al. Initial validation of the Activity Choice Index among overweight women. Res Q Exer Sport 2016;87(2):174-81.

57. Simpson E, Bradley J, Poliakov I, et al. Iterative Development of an online dietary recall tool: INTAKE24. Nutrients 2017;9(2):118.

58. Stunkard AJ, Messick S. The three-factor eating questionnaire to measure dietary restraint, disinhibition and hunger. J Psychosom Res1985;29(1):71-83.

59. Arnold TA, Johnston CS, Lee CD, et al. Eating in the absence of hunger in college students. Appetite 2015;92:51-56.

60. Tylka TL, Kroon Van Diest AM. The Intuitive Eating Scale–2: Item refinement and psychometric evaluation with college women and men. J Couns Psychol 2013;60(1):137.

61. Gormally J, Black S, Daston S, et al. The assessment of binge eating severity among obese persons. Addict Behav 1982;7(1):47-55.

62. Herdman M, Gudex C, Lloyd A, et al. Development and preliminary testing of the new five-level version of EQ-5D (EQ-5D-5L). Qual Life Res 2011;20(10):1727-36.

63. Tennant R, Hiller L, Fishwick R, et al. The Warwick-Edinburgh mental well-being scale (WEMWBS): development and UK validation. Health Qual Life Outcomes 2007;5(1):63.

For peer review only

64. Sniehotta FF, Schwarzer R, Scholz U, et al. Action planning and coping planning for long-term lifestyle change: theory and assessment. Eur J Soc Psychol 2005;35(4):565-76.

65. Sniehotta FF, Scholz U, Schwarzer R. Bridging the intention–behaviour gap: Planning, self-efficacy, and action control in the adoption and maintenance of physical exercise. Psychol Health 2005;20(2):143-60.

66. Ng JY, Ntoumanis N, Thøgersen-Ntoumani C, et al. Predicting psychological needs and well-being of individuals engaging in weight management: The role of important others. Appl Psychol Health Well Being 2013;5(3):291-310.

67. Sebire SJ, Standage M, Vansteenkiste M. Development and validation of the goal content for exercise questionnaire. J Sport Exer Psychol 2008;30(4):353-77.

68. Markland D, Ingledew DK. The measurement of exercise motives: Factorial validity and invariance across gender of a revised Exercise Motivations Inventory. Br J Health Psychol 1997;2(4):361-76.

69. Markland D, Tobin V. A modification to the behavioural regulation in exercise questionnaire to include an assessment of amotivation. J Sport Exer Psychol 2004;26(2):191-96.

70. Pelletier LG, Dion SC, Slovinec-D'Angelo M, et al. Why do you regulate what you eat? Relationships between forms of regulation, eating behaviors, sustained dietary behavior change, and psychological adjustment. Motiv Emot 2004;28(3):245-77.

For peer review only

724 71. Palmeira AL, Teixeira PJ, Branco TL, et al. Predicting short-term weight loss using four leading health behavior change theories. Int J Behav Nutr Phys Act 2007;4(1):14.

726 72. Gilbert P, Clarke M, Hempel S, et al. Criticizing and reassuring oneself: An exploration of forms, styles and reasons in female students. Br J ClinPsychol 2004;43(1):31-50.

728 73. Goss K, Gilbert P, Allan S. An exploration of shame measures—I: The other as Shamer scale. Pers Indiv Differ 1994;17(5):713-17.

730 74. Allan S, Gilbert P, Goss K. An exploration of shame measures—II: Psychopathology. Pers Indiv Differ 1994;17(5):719-22.

732 75. Gilbert P, Catarino F, Duarte C, et al. The development of compassionate engagement and action scales for self and others. J Compassionate Health Care 2017;4(1):4.

734 76. Sandoz EK, Wilson KG, Merwin RM, et al. Assessment of body image flexibility: the body image-acceptance and action questionnaire. J Contextual Behav Sci 2013;2(1-2):39-48.

736 77. Trompetter HR, Ten Klooster PM, Schreurs KM, et al. Measuring values and committed action with the Engaged Living Scale (ELS): Psychometric evaluation in a nonclinical sample and a chronic pain sample. Psychol Assess 2013;25(4):1235.

739 78. Carlson LE, Brown KW. Validation of the Mindful Attention Awareness Scale in a cancer population. J Psychosom Res 2005;58(1):29-33.

741 79. Gratz KL, Roemer L. Multidimensional assessment of emotion regulation and dysregulation: Development, factor structure, and initial validation of the difficulties in emotion regulation scale. J Psychopathol Behav Assess 2004;26(1):41-54.

For peer review only - http://bmjopen.bmj.com/site/about/guidelines.xhtml

80. Fresco DM, Moore MT, van Dulmen MH, et al. Initial psychometric properties of the experiences questionnaire: validation of a self-report measure of decentering. Behav Ther 2007;38(3):234-46.

81. Cohen S, Kamarck T, Mermelstein R. A global measure of perceived stress. J Health Soc Behav 1983;24(4):385-96.

82. Antony MM, Bieling PJ, Cox BJ, et al. Psychometric properties of the 42-item and 21-item versions of the Depression Anxiety Stress Scales in clinical groups and a community sample. Psychol Assess 1998;10(2):176.

83. Larsen TM, Dalskov S, Van Baak M, et al. The Diet, Obesity and Genes (Diogenes) Dietary Study in eight European countries–a comprehensive design for long-term intervention. Obes Rev 2010;11(1):76-91.

84. Cappelleri JC, Bushmakin AG, Gerber RA, et al. Evaluating the Power of Food Scale in obese subjects and a general sample of individuals: development and measurement properties. Int J Obes 2009;33(8):913.

85. Weinstein N, Przybylski AK, Ryan RM. The index of autonomous functioning: Development of a scale of human autonomy. J Res Pers 2012;46(4):397-413.

86. Grouzet FM, Kasser T, Ahuvia A, et al. The structure of goal contents across 15 cultures. J Pers Soc Psychol 2005;89(5):800.

87. Tylka TL. Development and psychometric evaluation of a measure of intuitive eating. J Couns Psychol 2006;53(2):226.

For peer review only

For peer review only - http://bmjopen.bmj.com/site/about/guidelines.xhtml

88. Fuglestad PT, Rothman AJ, Jeffery RW, et al. Regulatory focus, proximity to goal weight, and weight loss maintenance. Am J Health Behav 2015;39(5):709-20.

89. Kaasinen E, Mattila E, Lammi H, et al. Technology acceptance model for mobile services as a design framework. In:J. Lumsden ed. Human-Computer Interaction and Innovation in Handheld, Mobile and Wearable Technologies. Hershey: IGI Global 2011:80-107.

90. Nguyen J, Moorhouse M, Curbow B, et al. Construct validity of the eHealth literacy scale (eHEALS) among two adult populations: a Rasch analysis. JMIR Public Health Surveill 2016;2(1).

91. Ritchie J, Spencer L, O'Connor W. Carrying out qualitative analysis. In: Richie J, Lewis J, eds. Qualitative Research Practice. London: Sage Publications 2004:219-62.

92. Preacher KJ, & Selig, J. P. Monte Carlo method for assessing multilevel Mediation: An interactive tool for creating confidence intervals for indirect effects in 1-1-1 multilevel models [Computer software]. Available from http://quantpsy.org/.

93. Meads DM, Hulme CT, Hall P, et al. The cost-effectiveness of primary care referral to a UK commercial weight loss programme. Clin Obes 2014;4(6):324-32.

94. General Assembly of the World Medical Association. World Medical Association Declaration of Helsinki: ethical principles for medical research involving human subjects. J Am Coll Dent 2014;81(3):14.

95. Medical Research Council. MRC guidelines for management of global health trials - Involving Clinical or Public Health Interventions. MRC 2017.

For peer review only

For peer review only - http://bmjopen.bmj.com/site/about/guidelines.xhtml

https://mrc.ukri.org/documents/pdf/guidelines-for-management-of-global-health-trials/ (accessed 4 Jan 2019).

For peer review only

For peer review only - http://bmjopen.bmj.com/site/about/guidelines.xhtml

Table 1 WHO Trial Registration Data Set

**Data category**	**Information**
Primary registry and trial identifying number	ISRCTN: 88405328
Date of registration in primary registry	16/12/2016
Secondary identifying numbers	H2020-EU.3.1 Grant agreement ID: 643309
Source(s) of monetary or material support	European Commission, Horizon 2020 - EU Framework Programme for Research and Innovation.
Primary sponsor	University of Leeds, Leeds, UK.
Secondary sponsor(s)	The Parker Institute, Copenhagen, Denmark.<br>University of Lisbon, Lisbon, Portugal.
Contact for public queries	Prof. James Stubbs (r.j.stubbs@leeds.ac.uk)<br>School of Psychology<br>Faculty of Medicine and Health<br>University of Leeds<br>Leeds<br>LS2 9JT<br>United Kingdom<br>+44 (0)113 343 3476
Contact for scientific queries	Prof. James Stubbs (r.j.stubbs@leeds.ac.uk)
Public title	NoHoW: Evidence-based ICT tools for weight loss maintenance.
Scientific title	A 2 x 2 randomised controlled trial to evaluate the effectiveness of evidence-based information and communications technology behaviour change tools for weight loss maintenance in overweight/obese adults after clinically significant weight loss.
Countries of recruitment	1. University of Leeds (UK),<br>2. The Parker Institute (Denmark),<br>3. University of Lisbon (Portugal).

For peer review only - http://bmjopen.bmj.com/site/about/guidelines.xhtml

NoHoW weight loss maintenance trial

Health condition(s) or problem(s) studied	Adults aged 18 or older who have intentionally lost at least 5% of their body weight in the last 12 months, and initially had a BMI of 25 or more.
Intervention(s)	Participants are assigned to one of four interventions:<br>1. Self-monitoring only (self-weighing and activity tracker),<br>2. Self-regulation and motivation,<br>3. Contextual-behavioural Science-based Emotion Regulation (CBS-emotion regulation),<br>4. Combined self-regulation, motivation and CBS-emotion regulation.
Key inclusion and exclusion criteria	Inclusion criteria:<br>1. Aged 18 years or older.<br>2. BMI (prior to weight loss) of ≥25 kg/m2.<br>3. Verification of ≥5% of weight loss in the last 12 months and remain 5% below their highest weight.<br>4. Ability to use a smartphone and have access to smart phone, tablet or computer with internet access and Wi-Fi.<br>5. Ability to use standing scales for weight measurements and must not be over 150kg (the scale limit 158kg)<br><br>Exclusion Criteria:<br>1. Inability to give informed consent.<br>2. Lost weight due to illness or surgical procedures, including bariatric procedures.<br>3. Pregnant or breastfeeding.<br>4. Involvement in another research intervention study that confound with the aims of NoHoW (excluding local health interventions and weight management services).<br>5. Inability to follow written material or telephone conversations in the English, Danish or Portuguese language (depending on the centre).<br>6. Diagnosed with an eating disorder (e.g. anorexia nervosa, bulimia nervosa, or purging disorder).<br>7. Diagnosed with any condition that may interfere with increasing mild to moderate physical activities and which is unstable (i.e. untreated or unable to be controlled by medication).

For peer review only
For peer review only - http://bmjopen.bmj.com/site/about/guidelines.xhtml

	8. Recent diagnosis with type 1 Diabetes.
	9. Extensive planned travel, i.e. moving away or long-term travel abroad (e.g., more than four weeks)
	10. Living in the same household as existing participant in the trial.
Study type	Interventional, 2x2 factorial randomised controlled trial.
Date of first enrolment	20 March 2017
Target sample size	1600
Recruitment status	Completed (31/03/2018), no longer recruiting.
Primary outcome(s)	Weight (kg)
Key secondary outcomes	1. Body composition: (e.g., fat mass and fat free mass) and waist/Hip ratio.<br>2. Biomarkers: blood pressure, heart rate, cortisol (hair samples), hbA1c, Cholesterol.<br>3. Behavioural: physical activity, sleep and dietary intake.<br>4. Psychological moderators and mediators of change for: self-regulation and motivation (e.g., action planning and coping, self-efficacy); CBS-emotion regulation (e.g., weight focused self-criticism, five dimensions of mindfulness); stress management (e.g., perceived stress scale, depression, anxiety and stress); and, Quality of life and wellbeing.<br>5. Process evaluation<br>6. Cost-effectiveness analysis

For peer review only - http://bmjopen.bmj.com/site/about/guidelines.xhtml

Table 2. Study recruitment and intervention schedule (SPIRIT 2013 guidelines).

	**Study Duration**				
	**Recruitment**	**Baseline**	**6 months**	**12 months**	**18 months**
**TIME POINT**	t_0	t_1	t_2	t_3	t_4
RECRUITMENT					
Screening	X				
Informed consent		X			
Randomisation and allocation		X			
INTERVENTION					
Fitbit device training		X			
Intake24 (food diary) training		X			
Toolkit training (after randomisation)		X			
Arms 2-4 active intervention		♦——	——♦		
Arms 2-4 follow-up			♦——	———	——♦
Arm 1 (control) intervention		♦——	———	———	——♦

For peer review only

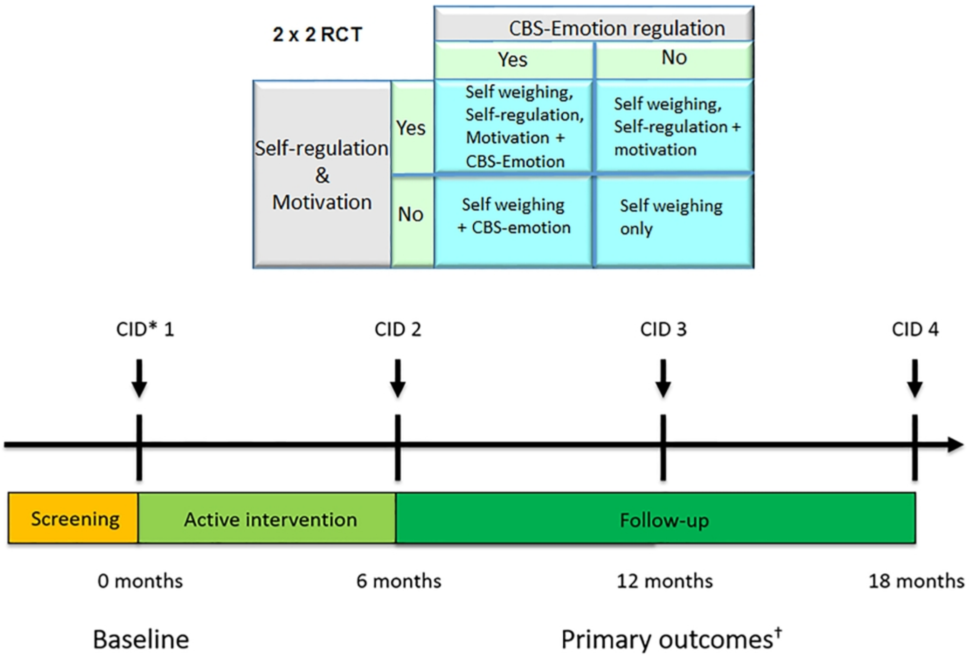

*CID, Clinical Investigation day.
†All measures are taken at CID 1-4 with the exception of the biomarkers which are taken at 0 and 12 months only. Process evaluation measures are taken at 1, 3, 6 and 12 months and focus groups at 6 months.

Figure 1. Trial design

220x178mm (300 x 300 DPI)

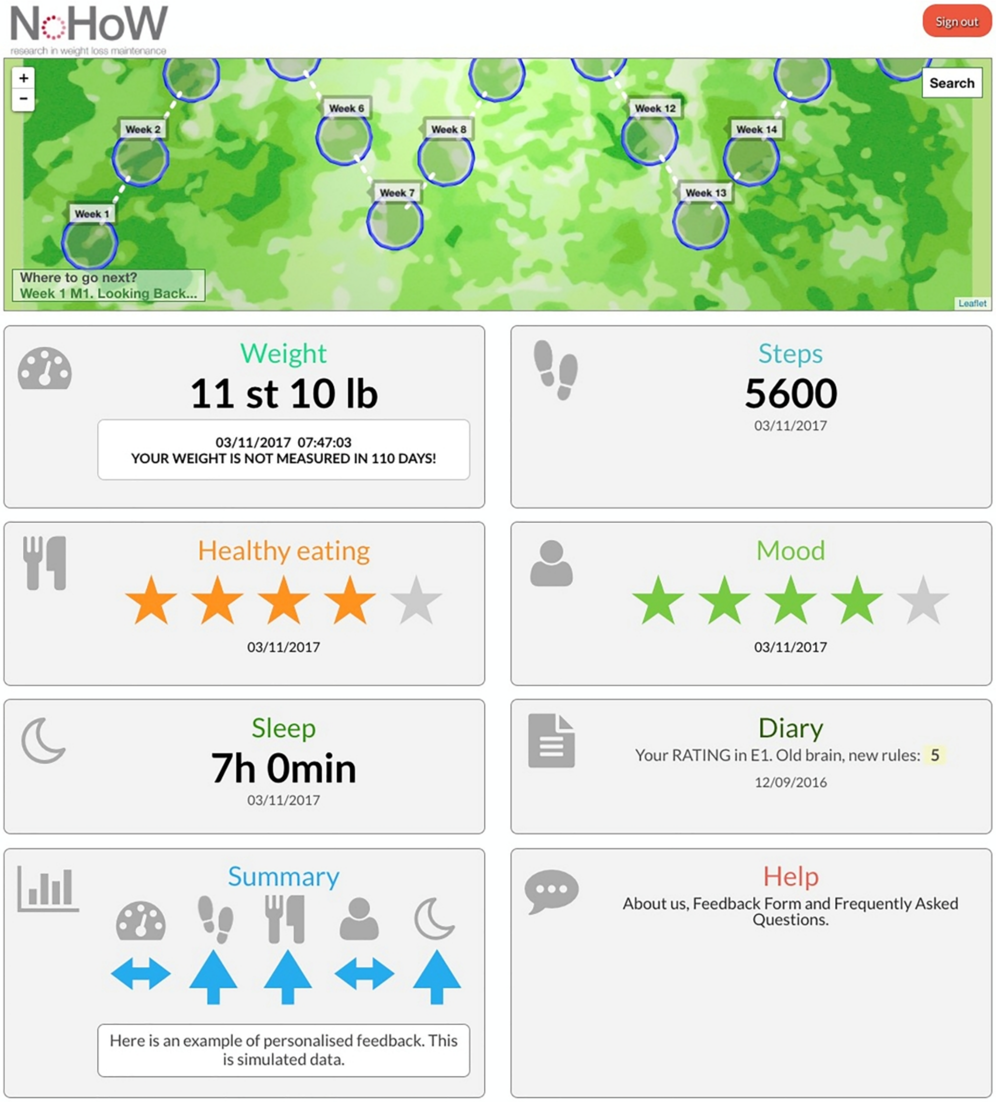

Figure 2. Toolkit example (arm 4).

375x417mm (300 x 300 DPI)

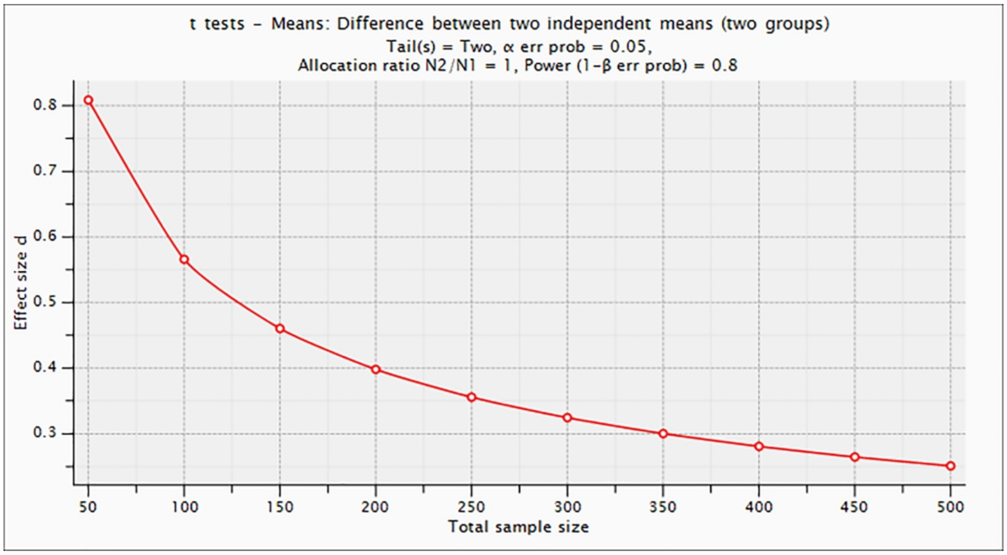

Figure 3. Plot of detectable effect size (Cohen's D)

197x108mm (300 x 300 DPI)

Supplementary file 1. Example consent form.

CONSENT FORM

NoHoW: Evidence-based ICT tools for weight loss maintenance

Please read the following statements and initial each box, as in order to continue in the study you must agree to the following statements

☐ I confirm that I have read and understood the participant information sheet (version 1, 27/02/2017), for the study named above and I have had the opportunity to ask questions and have had these answered satisfactorily.

☐ I consent for the research team to use the anonymised information obtained for research purposes (e.g., disseminating the results at conferences, in publications or for training purposes) and understand that the anonymised data will be kept for 20 years.

☐ I understand that information specific to this project may be looked at by responsible individuals from the research teams and will not be shared to anyone outside of the research teams.

☐ I understand that my participation is voluntary and that I am free to withdraw at any time, without giving any reason, without my medical care, or legal rights being affected.

☐ I agree to be contacted to be asked to fill in questionnaires and for measures to be taken initially at baseline, 6 months, 12 months and 18 months after the study has begun.

The following sections are optional- if you agree with a statement, please initial the box:

☐ I agree to take part in the study.

☐ I agree to provide a sample of a small section of hair, for the assessment of cortisol content, as a biomarker of longer term stress, initially at baseline and 12 months after.

For peer review only

Donation of blood for research is voluntary and you should not be placed under any pressures to do so. You do not have to agree to give a blood sample nor need to explain why you should choose not to donate. Any personal information provided by you in connection with the donation will be held in confidence. For reasons of safety, you should not donate if:

- You know, or think that you might be infected with Hepatitis B or Hepatitis C.
- You know, or think that you might be infected with HIV.
- You have a sexual partner who is infected with Hepatitis or HIV.
- You are unwell at the moment.
- You are anaemic or receiving treatment for anaemia or iron deficiency.

☐ I agree to provide a small blood sample by finger prick initially at baseline, and 12 months after.

☐ I wish to receive written feedback of my blood results which will include results of my full lipid profile (e.g., cholesterol), and HbA1c levels.

☐ Occasionally, results may fall out of the usual range which may be due to conditions such as high blood pressure, pre-diabetes, diabetes and high cholesterol. In these cases, we may wish to share the results of your measurements with yourself and your GP. I agree for the researchers to contact me and my GP

Name of participant: ……………………………………………

Signature of participant: ………………………………………… Date: ……………

I confirm that the volunteer named above has been instructed of the nature and purpose of the study to be undertaken.

Signature of researcher:……………………………………………………….. Date: ………………

The NoHoW Project has been approved by the School of Psychology Research Ethics Committee at the University of Leeds (17-0082; 27-Feb-2017). If you have any questions, please contact the principal Investigator, Professor James Stubbs, at r.j.stubbs@leeds.ac.uk.

For peer review only

Supplementary File 2. Study assessment schedule (SPIRIT 2013 guidelines).

		Study Duration				
		Recruitment	Baseline	6 months	12 months	18 months
**MEASURES**	**DESCRIPTION**	**t_0**	**t_1**	**t_2**	**t_3**	**t_4**
**DEMOGRAPHICS.**	Age, gender, occupation, education level, income, height, marital status and employment, smoking and alcohol history.	X	X			
**WEIGHT HISTORY.**	Self-report of current weight, weight 12 months ago, highest weight in last 12 months, highest weight, pregnancy, weight loss attempts, weight maintenance, relapse, weight loss methods, and parent's weight history.		X			
**MEDICAL HISTORY**	Name and dosage of medications, medical conditions, stability of conditions.	X	X	X	X	X
**DIGITAL HEALTH LITERACY.**	The eHEALS scale[84] assesses digital health literacy (i.e. knowledge of different health information on the internet and perceived confidence in one's ability to find or use such information to make health decisions). The scale consists of 8 items measured on a 5-point Likert scale ('1', Strongly disagree, to '5' Strongly agree).		X			
**ANTHROPOMETRICS**						
Weight.	Body weight (Seca 704s, SECA, Germany) in light clothing at 0,6, 12 and 18 months and at home throughout the duration of the trial via the Fitbit Aria smart scales ≥ twice weekly.		◆	—	—	◆

For peer review only

Height	Height (±0.1cm) is measured with participants barefoot, using a Seca 704s instrument (SECA, Germany).		X			
Body mass index	Body mass index (BMI) is calculated from weight (kg) and height (m) using the following equation: kg/m^2.	X	X	X	X	X
Waist hip ratio.	The waist measurement is taken against bare skin or light clothing at the top of the iliac crest and the hip measurement is taken at the maximum circumference over the buttocks using a tape measure. Average waist hip ratio measured by taking 2 fasted hip and waist circumference measures, or 3 if there is >1cm difference. The waist-hip ratio is calculated by dividing hip and waist circumference.		X	X	X	X
**BODY COMPOSITION**	Fasting bioimpedance (ImpediMed^{TM} SFB7, Queensland, Australia) estimates total body water, extra- and intracellular fluid (l) and hence fat free mass (kg) and by subtraction fat mass (kg). It includes applying an electrical current at 256 frequencies ranging from 4 kHz to 1000 kHz, via a tetrapolar electrode configuration, following manufacturer's instructions. Assuming a constant hydration factor of 0.73 for fat free mass and that fat mass is hydrophobic, body water and weight can be used to estimate fat and fat free mass using a 2 compartment model.		X	X	X	X
**BIO-MARKERS**						
HbA1c.	Fasting capillary blood is analysed for HbA1c (mmol/mol, eAG and %) using the Afinion^{TM} AS100 Analyzer (Alere, Stockport, UK). Sample cartridges are disposed of immediately after results are obtained from the machine.		X		X	
Full lipid profile.	Fasting capillary blood is analysed for full lipid profiles including total cholesterol (mmol/L), low density lipoprotein (LDL), high density lipoprotein (HDL), triglycerides, non-HDL and cholesterol/HDL (mmol/L) using the AfinionTM AS100 Analyzer (Alere). Sample cartridges are disposed of immediately after results are obtained from the machine.					
Hair samples.	Cortisol levels are measured by analysing 2cm of hair cut from close to the scalp. The sample is stored in		X		X	

For peer review only

	foil inside a sealed plastic bag. User ID and hair information (e.g., the use of hair dye, hair product use and hair wash frequency per week) is stored with the sample. Between 10 and 20 mg of hair from the 2 cm of the tuft closest to the scalp is accurately weighed, minced finely 1ml of methanol is added and the suspension incubated overnight at 50°C with a gentle shaking. The following day, the methanol is transferred into a clean tube and evaporated to dryness under nitrogen. The residue is reconstituted in 250-µl PBS buffer (pH 8.0). The cortisol concentration (pg/mg) in the resulting buffer solution is determined in duplicate using Enzyme-linked immunosorbent assay (ELISA).[48]					
Blood pressure.	An average of three fasting pulse, diastolic and systolic blood pressure measures are calculated and measured using the Microlife BP A2 (Gentle Technology, Microlife, Clearwater, FL, USA, Inc).		X	X	X	X
Heart rate.	Daily average resting heart rate is measured from minute-by-minute data collected throughout the duration of the trial via the Fitbit Charge 2.		◆	———	———	◆
**PHYSICAL ACTIVITY**						
Daily steps	Minute-by-minute steps are monitored by Fitbit Charge 2 for trial duration. The Charge 2 TM uses a wrist-worn tri-axial accelerometer, altimeter and vibration motor, which collects longitudinal movement data and estimates activity intensity, duration, number of steps, and distance travelled using proprietary algorithms (https://www.fitbit.com/charge2).		◆	———	———	◆
Intensity of physical activity (low, moderate and vigorous).	Time (minutes/day) spent in each intensity of activity is measured via self-report using the International Physical Activity Questionnaire (IPAQ) long-version[56] and is objectively measured using minute-by-minute activity monitored by Fitbit Charge 2 for trial duration.		X	X	X	X
Sitting behaviour.	Time (minutes/day) spent sitting is measured using the IPAQ.[56]		X	X	X	X
Activity choice.	The Activity Choice Index (ACI)[57] measures the ability to choose physical activity over less active or sedentary behaviours, such as using stairs or escalators. The scale consists of a 5-point Likert scale ('1',					

For peer review only

	never, to '5' always).					
**SLEEP QUALITY AND QUANTITY**	Sleep quantity (hours/day) and quality are monitored by the Fitbit Charge 2 for the trial duration.		◆	—	—	◆
**DIETARY INTAKE AND EATING BEHAVIOUR**						
Dietary intake.	Four consecutive days of 24-hour dietary recalls, including one weekend day, using the web-based tool INTAKE24[58], which measures food intake and estimates daily energy (kcal) and nutrient intake from standardised food tables. Country specific food tables were inserted into the platform.		X	X	X	X
Cognitive restraint, disinhibition and hunger.	The Three Factor Eating Questionnaire (TFEQ)[59] is a 51-item, 3 factor measure that assesses cognitive restraint, disinhibition and hunger. The scale uses a true or false scale (items 1-36), a 4-point scale for items 37-49 and 51 ('1', rarely, to '4', always) and a 6-point scale for item 50 ('0', I eat whenever I want, to '5', constantly limiting food intake).		X	X	X	X
Controllability and automaticity.	Controllability and Automaticity of Eating Behaviour Questionnaire (CAEBQ) (unpublished scale) is a 24-item, 2 factor measure assessing eating control, grazing, binging and eating in the absence of hunger. Each subscale consists of 2-items assessed on a 5-point scale ('1', never, to '5', very often) and 4 items consisting of a 5-point Likert scale ('1' strongly disagree, to '5', strongly agree).		X	X	X	X
Eating in the absence of hunger.	Eating in The Absence of Hunger Scale (EAHS)[60] is a 28-item, single factor scale assessing the conditions when one is likely to eat in the absence of hunger. The 28 items assesses eating in response to emotional, physical, and environmental cues, using a 5-point scale ('1', never, to '5', always).		X	X	X	X
Reliance on hunger and satiety.	The reliance on hunger and satiety cues is a 6-item subscale of the Intuitive Eating Scale-2 (IES-2)[61] that measures the ability to guide eating behaviour based on one's internal hunger and satiety signals. The subscale uses a 5-point Likert scale ('1', strongly disagree, to '5', strongly agree).		X	X	X	X

For peer review only

Binge Eating.	The Binge Eating Scale (BES)[62] is a 16-item one-dimensional measure assessing the severity of binge eating symptoms. Each item consists of three to four statements from which respondents select one. Each option reflects a rating of severity ranging from 0 (no difficulties with binge eating) to 3 (severe binge eating symptoms).		X	X	X	X
Intuitive eating – 1.	Intuitive Eating Scale – 1 (IES-1)[53] is a 21-item trait measure to assess the ability to be aware and follow physical hunger and satiety cues to determine when, what and how much to eat. It includes three subscales: eating for physical rather than emotional reasons, unconditional permission to eat and reliance on hunger and satiety cues. IES-1 uses a 5-point Likert scale ('1' strongly disagree, to '5' strongly agree). The full scale is used at baseline only.		X			
Power of food.	Power of Food Scale[52] is a 10-item measure adapted from the original 15 item version which assesses the sensitivity to palatable foods within food-abundant environments. It includes three subscales: food availability, food present and food tasted. PFS uses a 5-point Likert scale ('1' do not agree at all, to '5', strongly agree).		X			
**WELL-BEING AND QUALITY OF LIFE**						
Quality of Life.	The EQ5D-5L[63] measures generic health related quality of life (HRQoL). It includes five dimensions (mobility, self-care, usual activities, pain/discomfort, and anxiety/depression) and has five levels of severity within each dimension: no problems (Level 1); slight; moderate; severe; and extreme problems (Level 5).		X	X	X	X
Wellbeing.	The 14-item single factor Warwick and Edinburgh Well-being Scale[64] measures mental wellbeing using 14 positively worded statements and uses a 5-point scale ('1', none of the time, to '5', all of the time).		X	X	X	X
**SELF-REGULATION, SELF-EFFICACY AND**						

**MOTIVATION**						
Action planning and coping planning	The Action Planning and Coping Planning Scales (APCP)[65] measures coping planning as a barrier-focused self-regulation strategy. The Action planning scale consists of 4 items and coping planning consists of 15 items both using a 5-point Likert scale ('1', Strongly disagree, to '5', Strongly agree').		X	X	X	X
Action control	The Action Control Scale[66] consists of 8 items and uses a 5 point Likert scale ('1', Strongly disagree, to '5', Strongly agree') that measures three distinct self-regulatory actions: self-monitoring, awareness of standards, and effort.		X	X	X	X
Basic psychological needs and frustration	The Basic Psychological Needs and Frustration Scale[67] has 16 items that uses a 7-point scale ('1', Strongly disagree to, '7', Strongly agree') to assess basic need satisfaction with 4 subscales: autonomy, competence, social support and relatedness. Items 2, 4, 6, 8, 12, and 16 were adapted to focus on weight management (e.g. 'I feel capable of managing my weight').		X	X	X	X
Goal content	The Goal Content for Weight Loss Maintenance Scale (GCWLMS)[68,69] was modified to focus on weight management rather than exercise behaviours (e.g. 'I manage my weight to…'). It consists of 16 items that uses a 7-point scale ('1', Strongly disagree, to '7', Strongly agree') to assess intrinsic and extrinsic goals.		X	X	X	X
Behavioural regulation in exercise	The Behavioural Regulation in Exercise Scale (BREQ-3)[71] includes 4 subscales that measure external, introjected, identified and intrinsic forms of regulation of exercise behaviour. It consists of 24 items and uses a 5-point scale ('0', Not true for me, to '4', Very true for me).		X	X	X	X
Regulation for eating behaviour	The Regulation for Eating Behaviour Scale (REBS)[71] is the shortened scale and consists of 18 items that assess autonomous and controlled motivation, and associated types of regulation (e.g. introjected regulation) for eating behaviour, using a 7-point scale ('1', Does not correspond at all, to '7', Corresponds exactly). One item from each sub-scale (items 3, 6, 10, 11, 18, 20) displayed unsatisfactory psychometric properties and were removed to create a shortened scale.		X	X	X	X
Self-efficacy	The Self-Efficacy for Weight Management Scale (SEWM)[72] has 12 items and uses a 100 point sliding		X	X	X	X

For peer review only

	scale ('0', not confident at all, to '100', extremely confident) to asses self-efficacy beliefs for exercise and eating.					
Autonomy	The Index of Autonomous Functioning (IAF)[50] is a 15-item scale and uses a 5-point scale ('1', Not at all true, to '5', Completely true) to assess trait autonomy across three theoretically derived subscales: authorship/self-congruence, interest-taking, and low susceptibility to control.		X			
Relapse Prevention	The Relapse Prevention Scale (RPS)[54] is a newly developed scale for trait-level weight management and consists of 14 items that measures three sub-domains: approach-based plans/goals, avoidance-based plans/goals, and cognitive factors. The scales uses 5-point scale ('1', Very unlikely, to '5', Very likely).		X			
Life goals and aspirations	The Aspirations Index (AI)[51] is an 11-item scale that measures the degree to which people value seven broad goal contents: wealth, fame, image, personal growth, relationships, community contribution, and health. The scale uses a 9-point scale ('1', Not at all important, to '9', Extremely important).		X			
Regulation for weight management.	The Regulation for Weight Management Scale (RWMS)[71] consists of 10 items and measures the reasons that underpin individual's decisions to manage their weight (e.g. "Managing my weight is the part of the way I have chosen to live my life"). The scales uses a 7-point Likert scale ('1', Does not correspond at all, to '7', Corresponds exactly) and was adapted from the Regulation of Eating Behaviour scale (REBS) to focus specifically on weight management.		X	X	X	X
**EMOTION REGULATION**						
Self-criticism and reassurance	The Weight Focused Self-Criticism/Self-Reassuring Scale (WFSCRS)[73] is a 22-item scale that assesses one's thoughts and feelings about themselves during a perceived failure. Three subscales consist of self-criticising, (inadequate self and hated self) and tendencies to reassure the self (reassured self). The scale uses a 4-point scale ('0', Not at all like me, '4', Extremely like me) and was adapted to focus on weight, body shape, and eating.		X	X	X	X

For peer review only - http://bmjopen.bmj.com/site/about/guidelines.xhtml

External shame	The Weight-Focused External Shame Scale (WFESS)[74] is a one-dimensional measure with 18 items that measure external shame (e.g., thinking that others negatively evaluate the self) and uses a 4-point scale ('0', Never, to '4', Almost always).		X	X	X	X
Compassion	The Compassionate Attributes and Actions Scales (CAAS)[76] consist of three scales that measure self-compassion, compassion for others, and compassion from others. In this study, we omit the compassion for others scale as it is unrelated to the intervention logic models. Each scale consists of 6 items and uses a 10-point scale ('1', Never; to, '10', Always).		X	X	X	X
Body Image Acceptance	The Body Image Acceptance and Action Questionnaire (BIAAQ)[77] is a 12-item one-dimensional scale that measures body image-related psychological flexibility and the extent to which an individual accepts their beliefs and feelings about their body without attempting to change them while pursuing chosen values. The scale uses a 7-point scale ('1' Never true, to '7' Always true).		X	X	X	X
Engaged response (valued living and life-fulfilment)	The Engaged Living Scale (ELS)[78] consists of 16 items and measures two factors associated with psychological flexibility in line with Acceptance and Commitment Therapy: values living and life fulfilment. The scale uses a 5-point Likert Scale ('1', Completely disagree, to '5', Completely agree).		X	X	X	X
De-centering	The Experiences Questionnaire (EQ_DC)[79] has 11 items that assess three facets of the de-centering construct, including: (1) the ability to view the self as not synonymous with thoughts and feelings; (2) the ability to not habitually react to negative experiences ; and, (3) the capacity for self-compassion. The scale provides a total score of decentering and uses a 5-item scale ('1', Never, to '5', Always).		X	X	X	X
Mindful attention and awareness.	The Mindful Attention Awareness Scale (MAAS)[79] is a 15-item one-dimensional scale designed to assess dispositional mindfulness, that is, a receptive state of mind informed by a sensitive awareness of what is occurring in the present moment. The scales uses a 6-point scale ('1' Almost always, to '6' Almost never).		X	X	X	X
Emotion regulation.	The Difficulties in Emotion Regulation Scale (DERS)[80] is a one-dimensional scale that assesses the difficulty to regulate emotional responses by using adaptive emotion regulation strategies in the presence of negative emotions so that it does not lead to loss of control over behaviour or goal attainment. The scale consists of 16 items and uses a 5-point scale ('1', Almost always, to '5', Almost never) and is adapted for		X	X	X	X

For peer review only

	weight loss maintenance.					
**STRESS MANAGEMENT**						
Perceived stress.	The Perceived Stress Scale (PSS)[81] measures the degree to which people perceive their lives as stressful (unpredictable, uncontrollable and overloaded) in the last month. The scale is one-dimensional and consists of 4 items and uses a 5-point scale (‘0’, Never, to ‘4’, Very often).		X	X	X	X
Depression and anxiety.	The Depression and Anxiety Scale (DASS)[82] short version consists of 21 items and measures three related negative emotional states individuals experienced over the last week, through 3 subscales with 7 items each: depression, anxiety and stress. The scales uses a 4 point scale (‘0’, Did not apply to me at all, to ‘3’, Applied to me very much).		X	X	X	X
**PROCESS EVALUATION AND USER EXPERIENCE**						
Initial toolkit impressions.	Initial impressions of the NoHoW Toolkit are captured at 1 month post-baseline and is adapted from the Technology Acceptance Model for Mobile Services (TAMM).[83] The survey consists of 14 items and uses a 5-point Likert scale (‘1’, Strongly disagree, to ‘5’, Strongly agree) to measure ease of adoption, ease of use, values and trust. Two additional items using a 11-point scale (‘0’, Not at all likely, to ‘10’, Extremely likely) assess how likely one would consider using the service in the future or recommend to a friend. Users are asked to rate the toolkit overall out of 10 and an open text box is provided for additional comments.		X			
User experience.	User experience is assessed at 3, 6 and 12 months using the TAMM[83] that assesses ease of adoption, ease of use, value, trust, and general usability using 25 items on a 5-point Likert scale (‘1’, Strongly disagree, to ‘5’, Strongly agree). Users are asked to rate the degree of importance, convenience, enjoyment,			X	X	

For peer review only

	satisfaction, and motivation for 14 components of the Toolkit using a 5 point Likert scale ('1' Not at all, to '5', Very). Two additional items using a 11-point scale ('0', Not at all likely, to '10', Extremely likely) assess how likely one would consider using the service in the future or recommend to a friend. Users are asked to rate the toolkit overall out of 10 and an open text box is provided for additional comments. Three items also assess toolkit usage: (1) 'On average, how frequently do you use the toolkit'; (2) 'What option describes your toolkit use behaviour best?'; and, (3) 'What motivated you to continue/why did you quit using the toolkit?'.					
Meta-data	The data-hub will collect meta-data for individual-level toolkit interactions including engagement and dose, such as the pattern of Toolkit and module use, intensity of use (e.g., timing, frequency of logins and duration of module/sub-module use), usage and content patterns (e.g., percentage of tasks performed and pages visited). Data are analysed using Matlab (MathWorks, Inc., Natick, Massachusetts, USA).		X	X	X	X

For peer review only

Reporting checklist for protocol of a clinical trial.

Based on the SPIRIT guidelines.

Instructions to authors

Complete this checklist by entering the page numbers from your manuscript where readers will find each of the items listed below.

Your article may not currently address all the items on the checklist. Please modify your text to include the missing information. If you are certain that an item does not apply, please write "n/a" and provide a short explanation.

Upload your completed checklist as an extra file when you submit to a journal.

In your methods section, say that you used the SPIRIT reporting guidelines, and cite them as:

Chan A-W, Tetzlaff JM, Altman DG, Laupacis A, Gøtzsche PC, Krleža-Jerić K, Hróbjartsson A, Mann H, Dickersin K, Berlin J, Doré C, Parulekar W, Summerskill W, Groves T, Schulz K, Sox H, Rockhold FW, Rennie D, Moher D. SPIRIT 2013 Statement: Defining standard protocol items for clinical trials. Ann Intern Med. 2013;158(3):200-207

		Reporting Item	Page Number
Title	#1	Descriptive title identifying the study design, population, interventions, and, if applicable, trial acronym	1
Trial registration	#2a	Trial identifier and registry name. If not yet registered, name of intended registry	2, Table 1

For peer review only

Trial registration: data set	#2b	All items from the World Health Organization Trial Registration Data Set	Table 1 (page 7)
Protocol version	#3	Date and version identifier	7
Funding	#4	Sources and types of financial, material, and other support	Table 1, 21
Roles and responsibilities: contributorship	#5a	Names, affiliations, and roles of protocol contributors	21
Roles and responsibilities: sponsor contact information	#5b	Name and contact information for the trial sponsor	Table 1 (page 7)
Roles and responsibilities: sponsor and funder	#5c	Role of study sponsor and funders, if any, in study design; collection, management, analysis, and interpretation of data; writing of the report; and the decision to submit the report for publication, including whether they will have ultimate authority over any of these activities	N/A None
Roles and responsibilities: committees	#5d	Composition, roles, and responsibilities of the coordinating centre, steering committee, endpoint adjudication	7, 17, 21. Data monitoring committee is replaced with JHI, GH, data management

For peer review only

		committee, data management team, and other individuals or groups overseeing the trial, if applicable (see Item 21a for data monitoring committee)	and trial management meetings.
Background and rationale	#6a	Description of research question and justification for undertaking the trial, including summary of relevant studies (published and unpublished) examining benefits and harms for each intervention	3-6
Background and rationale: choice of comparators	#6b	Explanation for choice of comparators	3-6
Objectives	#7	Specific objectives or hypotheses	5-6
Trial design	#8	Description of trial design including type of trial (eg, parallel group, crossover, factorial, single group), allocation ratio, and framework (eg, superiority, equivalence, non-inferiority, exploratory)	6-7, 9 (for allocation)
Study setting	#9	Description of study settings (eg, community clinic, academic hospital) and list of countries where data will be	6-7

For peer review only

		collected. Reference to where list of study sites can be obtained	
Eligibility criteria	#10	Inclusion and exclusion criteria for participants. If applicable, eligibility criteria for study centres and individuals who will perform the interventions (eg, surgeons, psychotherapists)	7, Table 1. 9 (interventions conducted by trial staff)
Interventions: description	#11a	Interventions for each group with sufficient detail to allow replication, including how and when they will be administered	9-11
Interventions: modifications	#11b	Criteria for discontinuing or modifying allocated interventions for a given trial participant (eg, drug dose change in response to harms, participant request, or improving / worsening disease)	20
Interventions: adherance	#11c	Strategies to improve adherence to intervention protocols, and any procedures for monitoring adherence (eg, drug tablet return; laboratory tests)	11 (intervention adherence), 12 (participant retention to trial protocol)
Interventions: concomitant care	#11d	Relevant concomitant care and interventions that are permitted or prohibited during the trial	7, Table 1.

For peer review only

Outcomes	#12	Primary, secondary, and other outcomes, including the specific measurement variable (eg, systolic blood pressure), analysis metric (eg, change from baseline, final value, time to event), method of aggregation (eg, median, proportion), and time point for each outcome. Explanation of the clinical relevance of chosen efficacy and harm outcomes is strongly recommended	12-17, Supplementary file 2.
Participant timeline	#13	Time schedule of enrolment, interventions (including any run-ins and washouts), assessments, and visits for participants. A schematic diagram is highly recommended (see Figure)	Table 2, Supplementary file 2, Figure 1. Pp 7-11.
Sample size	#14	Estimated number of participants needed to achieve study objectives and how it was determined, including clinical and statistical assumptions supporting any sample size calculations	20
Recruitment	#15	Strategies for achieving adequate participant enrolment to reach target sample size	8

For peer review only

Allocation: sequence generation	#16a	Method of generating the allocation sequence (eg, computer-generated random numbers), and list of any factors for stratification. To reduce predictability of a random sequence, details of any planned restriction (eg, blocking) should be provided in a separate document that is unavailable to those who enrol participants or assign interventions	9
Allocation concealment mechanism	#16b	Mechanism of implementing the allocation sequence (eg, central telephone; sequentially numbered, opaque, sealed envelopes), describing any steps to conceal the sequence until interventions are assigned	9
Allocation: implementation	#16c	Who will generate the allocation sequence, who will enrol participants, and who will assign participants to interventions	9
Blinding (masking)	#17a	Who will be blinded after assignment to interventions (eg, trial participants, care providers, outcome assessors, data analysts), and how	9

For peer review only

Blinding (masking): emergency unblinding	#17b	If blinded, circumstances under which unblinding is permissible, and procedure for revealing a participant's allocated intervention during the trial	9
Data collection plan	#18a	Plans for assessment and collection of outcome, baseline, and other trial data, including any related processes to promote data quality (eg, duplicate measurements, training of assessors) and a description of study instruments (eg, questionnaires, laboratory tests) along with their reliability and validity, if known. Reference to where data collection forms can be found, if not in the protocol	12-17 and suppl. File 2.
Data collection plan: retention	#18b	Plans to promote participant retention and complete follow-up, including list of any outcome data to be collected for participants who discontinue or deviate from intervention protocols	12
Data management	#19	Plans for data entry, coding, security, and storage, including any related processes to promote data quality (eg, double data entry; range checks for data values). Reference to where	17-18

For peer review only

		details of data management procedures can be found, if not in the protocol	
Statistics: outcomes	#20a	Statistical methods for analysing primary and secondary outcomes. Reference to where other details of the statistical analysis plan can be found, if not in the protocol	18-20
Statistics: additional analyses	#20b	Methods for any additional analyses (eg, subgroup and adjusted analyses)	18-20
Statistics: analysis population and missing data	#20c	Definition of analysis population relating to protocol non-adherence (eg, as randomised analysis), and any statistical methods to handle missing data (eg, multiple imputation)	18-20
Data monitoring: formal committee	#21a	Composition of data monitoring committee (DMC); summary of its role and reporting structure; statement of whether it is independent from the sponsor and competing interests; and reference to where further details about its charter can be found, if not in the protocol. Alternatively, an explanation of why a DMC is not needed	17-18, there is no data monitoring committee, agreed by the Trial Steering Committee and is replaced by James Hutton Institute who developed the data-hub, led by GH.

For peer review only

Data monitoring: interim analysis	#21b	Description of any interim analyses and stopping guidelines, including who will have access to these interim results and make the final decision to terminate the trial	No interim analyses were used as stopping guidelines. The stop/go decision was determined by whether recruitment targets were met. These targets were achieved, as recorded on the trial registration.
Harms	#22	Plans for collecting, assessing, reporting, and managing solicited and spontaneously reported adverse events and other unintended effects of trial interventions or trial conduct	20-21
Auditing	#23	Frequency and procedures for auditing trial conduct, if any, and whether the process will be independent from investigators and the sponsor	N/A
Research ethics approval	#24	Plans for seeking research ethics committee / institutional review board (REC / IRB) approval	20-21
Protocol amendments	#25	Plans for communicating important protocol modifications (eg, changes to eligibility criteria, outcomes, analyses) to relevant parties (eg, investigators,	20-21

For peer review only

		REC / IRBs, trial participants, trial registries, journals, regulators)	
Consent or assent	#26a	Who will obtain informed consent or assent from potential trial participants or authorised surrogates, and how (see Item 32)	8
Consent or assent: ancillary studies	#26b	Additional consent provisions for collection and use of participant data and biological specimens in ancillary studies, if applicable	N/A
Confidentiality	#27	How personal information about potential and enrolled participants will be collected, shared, and maintained in order to protect confidentiality before, during, and after the trial	17-18
Declaration of interests	#28	Financial and other competing interests for principal investigators for the overall trial and each study site	21-22
Data access	#29	Statement of who will have access to the final trial dataset, and disclosure of contractual agreements that limit such access for investigators	22
Ancillary and post trial care	#30	Provisions, if any, for ancillary and post-trial care, and for compensation to	N/A we do not expect participants to be harmed, but

For peer review only

		those who suffer harm from trial participation	interventions will be discontinued from the trial if participants deem it detrimental to their well-being (p20).
Dissemination policy: trial results	#31a	Plans for investigators and sponsor to communicate trial results to participants, healthcare professionals, the public, and other relevant groups (eg, via publication, reporting in results databases, or other data sharing arrangements), including any publication restrictions	21
Dissemination policy: authorship	#31b	Authorship eligibility guidelines and any intended use of professional writers	21
Dissemination policy: reproducible research	#31c	Plans, if any, for granting public access to the full protocol, participant-level dataset, and statistical code	22
Informed consent materials	#32	Model consent form and other related documentation given to participants and authorised surrogates	Suppl file 1.
Biological specimens	#33	Plans for collection, laboratory evaluation, and storage of biological	Suppl file 2 (Blood samples are analysed by the Afinion

For peer review only

		specimens for genetic or molecular analysis in the current trial and for future use in ancillary studies, if applicable	Analyser and disposed of straight away).

The SPIRIT checklist is distributed under the terms of the Creative Commons Attribution License CC-BY-ND 3.0. This checklist can be completed online using https://www.goodreports.org/, a tool made by the EQUATOR Network in collaboration with Penelope.ai

For peer review only
